# Cell-associated galectin 9 interacts with cytotoxic T cells confers resistance to tumor killing in nasopharyngeal carcinoma through autophagy activation

**DOI:** 10.1038/s41423-024-01253-8

**Published:** 2025-02-05

**Authors:** Ngar-Woon Kam, Cho Yiu Lau, Jeffrey Yan Ho Lau, Xin Dai, Yusi Liang, Syrus Pak Hei Lai, Michael King Yung Chung, Valen Zhuoyou Yu, Wenting Qiu, Mengsu Yang, Corey Smith, Rajiv Khanna, Kwan Ming Ng, Wei Dai, Chi Ming Che, Victor Ho-Fun Lee, Dora L. W. Kwong

**Affiliations:** 1 Department of Clinical Oncology, Centre of Cancer Medicine, School of Clinical Medicine, LKS Faculty of Medicine, The University of Hong Kong, Hong Kong, China; 2 Laboratory of Synthetic Chemistry and Chemical Biology Limited, Hong Kong, China; 3 LKS Faculty of Medicine, The University of Hong Kong, Hong Kong, China; 4 Department of Chemistry, The Hong Kong University of Science and Technology, Hong Kong, China; 5 Department of Biomedical Sciences and Tung Biomedical Sciences Centre, City University of Hong Kong, Hong Kong, China; 6 QIMR Centre for Immunotherapy and Vaccine Development and Department of Immunology, QIMR Berghofer Medical Research Institute, Brisbane, QLD Australia; 7 Department of Chemistry, Faculty of Science, The University of Hong Kong, Hong Kong, China; 8 Clinical Oncology Center, The University of Hong Kong-Shenzhen Hospital, Shenzhen, China

**Keywords:** Galectin 9, Cytotoxic T cells, Cell death, Tumor microenvironment, Immunotherapy, Nasopharyngeal carcinoma, Tumour immunology, Cell death and immune response, Cancer microenvironment

## Abstract

Immune effector cells, including cytotoxic T lymphocytes (CTLs) play essential roles in eliminating cancer cells. However, their functionality is often compromised, even when they infiltrate the tumor microenvironment (TME) or are transferred to cancer patients adoptively. In this study, we focused on galectin 9 (G9), an inhibitory ligand that we observed to be predominately positioned on the plasma membrane and readily interacts with CD8 + CTL in the TME of nasopharyngeal carcinoma (NPC). We discovered that cell-cell contact between activated effector CTLs and target tumor cells (TarTC) with G9 overexpression led to cellular death defects. Despite the formation of CTL–TarTC conjugates, there is no impact on the cell number nor viability of CTL, and the release of cytolytic content and associated activity were not completely abrogated. Instead, this interaction promoted autophagy and restricted necrosis in the TarTC. Furthermore, reducing G9 expression in tumor cells enhanced the suppressive effect on tumor growth upon adoptive transfer of activated effector CTL. Additionally, inhibiting autophagy effectively controlled tumor growth in cases of G9 overexpression. Therefore, we highlight the contribution of G9 in facilitating the resistance of NPC to CTL-mediated killing by inducing a selection-cell death state in tumor cells, characterized by increased autophagy and decreased necrosis.

## Introduction

Cytotoxic T lymphocyte (CTLs) are immune cells that play a vital role in killing tumors but are rendered dysfunctional after entering the hostile tumor microenvironment (TME). This tumor resistance to cell death also contributes to the tumor’s ability to resist treatment. Nasopharyngeal carcinoma (NPC) is known for expressing high levels of tumor-specific Epstein Barr Virus (EBV) antigens [1], making it an excellent model for evaluating the clinical potential of adoptive T-cell therapy (ACT) using EBV-specific CTLs (EBVSTs) [2]. While there is strong evidence of the antitumor activity of these CTLs in NPC, response rates vary between reported clinical trials [3–5]. Additional immunotherapies (IMT), such as immune checkpoint blockade (ICB), have been used to counter tumor-mediated immune suppression via targeting inhibitory ligands and receptors on tumor cells or T cells, respectively [6]. However, ICB alone has low response rate in NPC. While it is clear that tumor cell killing is reduced in the presence of an immunosuppressive TME, it is still uncertain as to what cellular events in target tumor cell (TarTC) that lead to their resistance to CTL-mediated killing and how such impairment is regulated by the established mediators of tumor-mediated immune suppression.

The killing of TarTC by CTLs requires carefully orchestrated steps that involve transient cell–cell interaction and paracrine release of cytolytic molecules directed toward TarTC [7]. Although individual CTLs have the ability to bind and attack multiple TarTC sequentially [8], the high efficacy of CTL-mediated serial killing is rarely observed in patients undergoing ACT [9]. In addition to evidence supporting the predominant short-lived interactions between TarTC and CTLs, non-productive CTL interactions could also hinder TarTC eradication [10, 11]. Higher local CTL densities have been linked to successful CTL effector function, increasing the likelihood of effective killing through multiple CTL-tumor cell interactions [12]. However, despite the physiological significance of CTL-tumor cell-cell contacts, the mechanism by which an “inefficient” CTL contacts, influenced by TarTC, contribute to the failure of eradication remains unclear. Unveiling the crosstalk between TarTC and CTL within TME is crucial for understanding how tumor-intrinsic vulnerabilities connect to immune evasion mechanisms and response to IMT.

Galectins (Gs) are a group of mammalian carbohydrate-binding proteins which have attracted much attention in the context of tumor immunity and IMT due to their ability to regulate inflammatory processes. Galectin-9 (G9) is a unique member of tandem-repeat-type subfamily [13]. Similar to other galectins, the mechanisms by which G9 exerts its immunomodulation are multifaceted [14]. Moreover, G9 interacts with multiple co-inhibitory receptors on T-cell surfaces, including T-cell immunoglobulin and mucin domain 3 (Tim3), programmed cell death protein 1 (PD-1), 4-1BB, V-domain Ig-containing suppressor of T cell activation (VISTA), CD44 and DR3. As a result of its selective T-cell interaction, G9 is crucial in modulating T-cell activities [15, 16] leading to its potential ability to regulate tumor surveillance [17]. Published studies demonstrated that G9-mediated lysosomal damage may facilitate lysosome-mediated autophagy [18] and increase the activity of AMP-activated protein kinase (AMPK) [19]. Autophagy, a critical process for cellular homeostasis, is not only involved in immune evasion in established tumors [20] but also has immunological functions that involve complex interactions with immune cells in the TME [21]. These interactions, in turn, affect the susceptibility of tumors to CTL-mediated cell lysis [22–24] and reduce their susceptibility to NK cell-mediated lysis. Such responses can be restored through autophagy inhibition [25]. G9 has been consistently detected in NPC cell lines, xenografted tumors, and clinical specimens, suggesting its involvement in the pathogenesis of NPC [26]. Thus, we hypothesized that NPC-associated G9 also plays a role in modulating the autophagic pathway in tumors and governing the CTL-mediated cytotoxicity.

We report for the first time that intimate contact between CD8 + T cells and neighboring tumor cells expressing membranous G9 within NPC tissues creates a protective shield against tumor destruction. Our work reveals that increased G9 expression in NPC cells led to a stronger cell-cell contact between TarTC and CTL. This G9-induced cell interaction drives a selection of cell-death modes characterized by elevated autophagy and limited necrosis, establishing a defense mechanism against CTL-induced tumor cell death. Furthermore, transferring in vitro–activated CTL into NPC–bearing mice showed enhanced tumor control when G9 expression was reduced or autophagy was suppressed. Our study uncovers a unique cellular process of tumor-induced immune suppression characterized by the “ineffective” maintenance of CTL–TarTC connections mediated by the inhibitory molecule G9.

## Results

### Clinical predictive value of the tumor-CD8+ immune microenvironment in G9-positive NPC

**Fig. 1:**
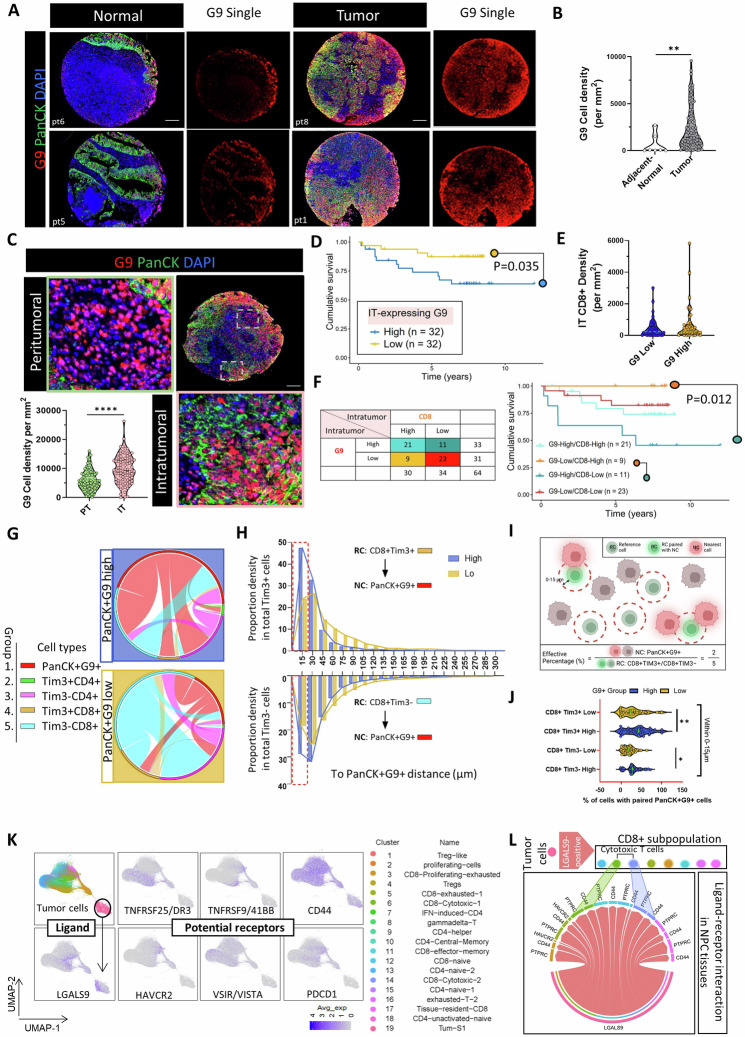
Distinct distribution of G9-expression profile and CD8^+^ T cell identifies patients with poor prognosis in NPC. **A** Representative NPC tissue specimens stained for pan-cytokeratin-5 (PanCK; green), G9 (red), and nuclei (DAPI; blue). Scale bar: 100 μm. **B** Density of G9^+^ cells as counts/mm^2^ in tumor samples (*n* = 92) and adjacent normal tissues (*n* = 7). **C** Representative image of intratumor (IT; red) and peritumor (PT; green) regions of NPC tumors. Density of G9^+^ cells as cell density/mm^2^ in IT and PT region (*n* = 87, 5/92 samples were excluded due to bad quality of staining). Scale bar: 100 μm. **D** Kaplan–Meier overall survival analysis based on IT-expressing G9 expression. **E** Density of CD8+ cells in the IT region of G9 high and low tumors. **F** IT-associated CD8 T cell and G9 expression of cancer patients (median as cut-off). Kaplan–Meier overall survival analysis based on IT-associated CD8 T cell and G9 expression. **G** Circos plots displayed the cell types of the nearest neighbor for each given cell excluding their own phenotype. The bands connecting the phenotypes indicate the cell interactions and the thickness of each bridge represents the number of cell relationships. The arc represents the total number of interactions of indicated cell phenotype. **H** Proportion of reference cells (RC) within a certain nearest distance (0–300 µm) between RC and nearest cells (NC: G9-expressing tumor cells; PanCK+G9+). **I** Illustration of the spatial analysis involving tumor cells and immune cells. **J** Percentage of CD8 T-cell populations (Tim3^+^ or Tim3^−^) paired with PanCK+G9+ cells within 15 µm. Dashed green line represent median. **K** UMAP plot of pooled infiltrating T cells and tumor cells from NPC tumors (GSE150430, GSE162025, and GSE150825). **L** Interaction between ligands (LGALS9-positve tumor cells) and indicated receptors (*p* < 0.05) expressed on CD8+ subpopulations. Avg = average. Data points are shown ± SEM. *p < 0.05, **p < 0.01, ***p < 0.001. (Mann–Whitney test for (**B**, **E**, **J**), and Kaplan–Meier curves were analyzed using two-tailed log-rank test for (**D**, **F**))

Multiplex immunohistochemical (mIHC) staining based on tyramide signal amplification (TSA) and quantitative analyses showed increased expression levels of G9 in tumor tissues from a cohort of 92 NPC patients (Fig. 1A, B). This upregulation was confirmed in two independent cohorts by comparing NPC and non-cancerous nasopharyngeal tissues at the mRNA level from public data (Supplementary Fig 1A). We next assessed whether G9 was expressed in a spatially coordinated manner using a marker for epithelial cells, Pan-cytokeratin (PanCK). Intratumoral [IT; defined by PanCK-positive (PanCK+) epithelial tumor cells)] and peritumoral tissue regions [(PT; defined by PanCK-negative (PanCK-) stromal cells; Fig. 1C and Supplementary Fig 1B]). G9^+^ cells were found in both IT and PT regions of tumor tissue, where the former region showed ~1.7-fold higher expression of G9. To assess the clinical relevance of our findings, we performed Kaplan‒Meier survival analysis of the G9-content in the IT tumor region. The overall survival rate (OSR) at 5 years was 68.75% (22/32) for patients with G9-high expression versus 81.25% (26/32) for those with G9-low expression (Fig. 1D; median G9 positivity as cutoff point; Log-rank *p* = 0.035), suggesting that the upregulation of G9 expression in tumor cells is associated with poor prognosis. We also analyzed the clinical predictive value of the tumor-immune microenvironment in G9-positive NPC. For this purpose, we used location-assisted CD8 + T-cell stratification based on G9 high/low expression in tumor cells (median as cutoff point). We observed that the number of CD8 located in the IT region was not impacted by the level of G9 expression on tumor cells (Fig. 1E), but those patients with CD8-low localization in the IT region and G9-high tumors had a significantly lower OSR at 5 years (63.64%; 7/11; turquoise line) compared to those with CD8-high localization in the IT region and G9-low tumors (100%; 9/9; *p* = 0.012; orange line; Fig. 1F). Of note, even in cases where CD8-high infiltration was present in the IT region but localized in G9-high tumors, patients’ survival, although not statistically significant, still exhibited a lower OSR at 5 years compared to those with G9-low tumors (cyan vs. orange line, respectively). This highlights that the outcomes of NPC patients may be associated with T cells that reside in a specialized tumor niche rich in G9 expression. A similar, albeit weaker, relationship was observed for CD8-low localization in the PT region (*p* = 0.056; Supplementary Fig 1C median as cutoff point). Likewise, the frequency of CD8 + T cells in the IT region of G9-high tumors was significantly higher than in G9-low tumors (IT: *p* = 0.00582; Chi-square test). However, there was no significant difference in the frequency of CD8 + T cells in the PT region between G9-high and G9-low tumors (*p* = 0.3152, Chi-square test). These findings suggest that CD8^+^ cells that are intermingled with tumor nest rather than being located in the tumor stroma hold clinical significance in the tumor mass.

These data collectively suggested that the level of G9 expression on tumor nest could have a significant impact with tumor-infiltrating CD8 + T cells in the same region.

### Spatial organization of tumor and T cells associated with G9-positivity in NPC

Next, we studied the spatial relationship between T-cell populations (CD4^+^ and CD8^+^ T cells), G9-expressing tumor cells (PanCK+G9 + ), and Tim3 expression on T cells. We found that the spatial distribution of CD8^+^ T cells (irrespective of Tim3 status) was influenced by the abundance of G9 in tumor cells, while the distribution of CD4^+^ T cells was not (Supplementary Fig 1D, E). Likewise, we observed a significant negative association between PanCK+G9+ and CD8+, but not CD4+, populations (CD8+ cells, *r* = −0.33; CD8+Tim3+ cells, *r* = −0.22; CD8+Tim3- cells, *r* = −0.3) (Supplementary Fig. 1F). Interestingly, although we observed a higher density of Tim3^+^ cells in the IT regions compared to the PT regions (Supplementary Fig 1G), majority of CD8^+^ infiltrates that intermingled with tumor cells were rarely positive for Tim3 in G9-highly positive tumors (Group 4 vs. Group 5 cells with Group 1 cells; Fig. 1G).

Notably, across all cell types tested, majority of T cell-tumor pairings (CD4+ vs. CD8 + ) were found within a radius of 0–15 µm (Fig. 1H and Supplementary Fig 1H). To further characterize the CTL–tumor cell contacts, we incorporate both cell proximity and quantity and introduce an “effective percentage” parameter as previously described [27] (Fig. 1I). A preselected distance range of 15 µm radius was utilized to identify CD8 + T-cell profiles capable of engaging in effective cell-to-cell interactions with G9+ tumor cells. Within this effective distance range, a higher proportion of Tim3- cells compared to Tim3+ cells (82.63% vs 17.37%) was observed. Furthermore, the effective percentage of CD8+ cells, irrespective of Tim3+ or Tim3-, was higher in G9-rich expressing tumor cells compared to low-G9 expressing tumor cells. However, within G9-highly expressing tumor cells, no difference in effective percentage was observed between CD8+Tim3+ and CD8+Tim3- cells (Fig. 1J).

In conclusion, these data indicate that CD8 + T cells tended to reside in close proximity within 15 µm distance to G9-expressing tumors, suggesting a higher probability of potential interaction, but this conjugation was likely to be independent of Tim3 expression.

### Immune-microenvironmental features in tumor tissues of G9-positive NPC

To further understand the heterogeneity of G9–receptor interactions on CD8 + T-cell subsets at the molecular level, three combined single-cell databases GSE150430 [28], GSE150825 [29], and GSE162025 [30] were analyzed and we calculated the probability of actual physical interaction of the cell pairs. After standard scRNAseq data analysis, eight clusters of CD8^+^ T cells were identified within tumor-infiltrating CD8 + T cells based on their expression of classical cell type markers and reference component analysis (Fig. 1K). We found that Tim3 (HAVCR2) was weakly expressed on all CD8^+^ T-cell populations in NPC tumors, including those with cytotoxic gene signatures (cluster 6 and 14). This finding was consistent with mIHC analyses and suggested that Tim3/G9 interaction may not be a prominent feature in NPC. Importantly, there were multiple ligand-receptor interactions within the same tumor-CD8 pairs, and among the recognized G9 interacting partners, PTPRC (CD45) that bears glycan chains, was likely to be the major counterpart (Fig. 1L).

Collectively, our result indicates there is CTL–G9+ tumor cell interaction in NPC tissues where this spatial juxtaposition likely involved the glycans on immune cell surface as the attachment factors.

### Physical interaction of CD8 + T cells with their target G9-expressing tumor cells

**Fig. 2:**
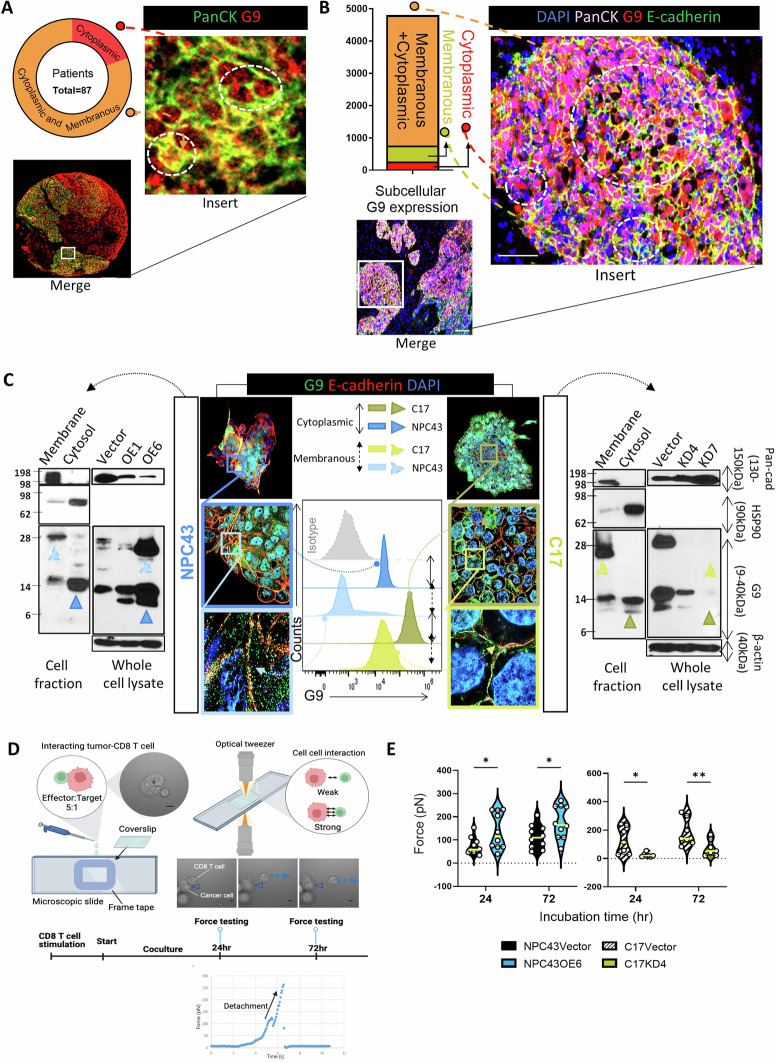
Physical interaction between membrane-bound G9 and CD8^+^ T cells. **A** Representative NPC tissue specimen showed membranous and cytoplasmic patterns for G9 (G9 in red; PanCK in green; double-positive as yellow). **B** Representative images of NPC tumors stained with antibodies against E-cadherin (green), G9 (red), epithelial tumor cells (pink) as well as the nuclear stain DAPI. Rectangles were enlarged as indicated in the images on the side. Colocalization of E-cadherin and G9 were indicated as yellow. Stacked bars illustrate its subcellular distribution (*n* = 6; NPC tissue samples (3 region-of-interest counted per slides). Bar: 100 μm. Magnified: 50 μm. **C** Immunostaining with E-cadherin (red) and G9 (green) and DAPI. Colored rectangles were enlarged as indicated in the images. Histograms and Western blot showed the membranous and cytoplasmic levels of G9 in vector control (NPC43 and C17), G9-overexpressed (OE; NPC43OE1 and NPC43OE6) and G9-knockdown (C17KD4 and C17KD7) cells. **D** Schematic representation of cell-cell interaction established between an NPC cell and purified CD8 + T-cell in a coculture system of a 5:1 effector-target ratio. Initially, the measured trapping force is zero (blue line) when the T-cell is pulled away and an increasing force is measured. When the applied force is high enough to break the bond between the two cells (blue dotted line), the measured force drops to zero. The cell-cell adhesion force was measured which is consistent with the expected adhesion force of a single T-cell. **E** Cell-cell adhesion forces measured for G9-knockdown or overexpressed NPC cells coincubated with T-cells at 24 and 72 h of coculture. Each violin represents the data for two set of 8-9 different samples of each cell type. The data were pooled from three (*n* = 3) independent experiments. *P < 0.05 and **P < 0.01 (two-way ANOVA for E). The data are presented as the mean ± SEM

It has been reported that G9 expressed in various cellular compartments, including the nucleus, cytoplasm, outer plasma membrane, and extracellular matrix [31, 32]. We sought to explore whether the CD8 T-cell’s proximity to G9-rich tumor beds is attributable to CD8 T cells contact with G9 expressed on the tumor cell plasma membrane. In this study, membranous form of G9 were frequently found in the NPC patient tissues which account for 80% (70/87) in our cohort (Fig. 2A). We next quantify the subcellular forms of G9 expression in patient tumor tissues. It was found that G9 often colocalized with E-cadherin, a membrane marker, accounting for 82.5% of total G9^+^ cancer cells that expressed both cytosolic and membranous form of G9, as shown by confocal microscopy (Fig. 2B). Very few (6.6%) G9 proteins were localized solely as cytoplasmic form. These findings suggest that most G9 is located on the plasma membrane in NPC tissue, which in turn increases the likelihood of engagement between G9 and local CD8 + T cells.

Then we further detected the subcellular localization of G9 expression of NPC cell lines. NPC43 and C17 were selected from a panel of patient-derived NPC cell lines according to their differential G9 expression levels (Supplementary Fig 2A). Staining of NPC cells with G9 and E-cadherin revealed not only its expression in the cytosol but also a significant distribution of G9 on the cell surface, particularly in C17 and to a lesser extent in NPC43. (Fig. 2C; middle panel). We next validated the subcellular distribution of G9 on tumor cells by performing a cell fractionation experiment, and the cytoplasmic and membrane fractions were separated and subjected to western blotting. The marker protein G9 detected as ~28 kDa membranous and ~14 kDa cytosolic form (Fig. 2C; side panels). Flow cytometry analysis further confirmed that 62.4% of NPC43 cells and 96.3% of C17 cells were cytosolic-associated G9, whereas 69.3% of C17 cells were membrane-associated G9 and the NPC43 cells contained only 5.48% expression in the same compartment (Fig. 2C; center panels).

On the assumption that T cell biology is modulated after physical contact with tumor cell-surface G9, we first explored the cell-cell adhesion force between G9-associated tumor-T cell pairs. To study the role of G9, we generated the stable G9- knocked down (KD) in C17 or G9-overexpression (OE) in NPC43, and defined as the target tumor cells (TarTC). We selected the best clone for subsequent gain-of-function (GOF: NPC43OE6) and loss-of-function (LOF: C17KD4) studies. We used an imaging modality on a culture platform which entailed coculturing of NPC TarTC and CD8 + T cells. Our method was based on the attachment of the two interacting cell types to opposing coverslips and then pulling them away during live-cell imaging, by which a cell-cell adhesion force was measured. Upon attachment to cancer cells, cell conjugates were observed within the 72 h duration of the experiment (Fig. 2D). During the coculture, T cell moved towards the cancer cell and contact was established. We observed that the force required to break the cellular contact in GOF system (NPC43OE6) was higher than those with G9 knockdown (C17KD4) and followed a time-dependent response (Fig. 2E). Similarly, a cell-cell adhesion assay confirmed that PKH26-labeled CD8^+^ cells had a higher tendency to adhere to PKH67-labeled tumor cells after overexpressing G9 in NPC (NPC43OE6) versus the knockdown group (C17KD4; Supplementary Fig 2B).

These observations collectively demonstrate that tumor surface-based G9 is a potential subcellular compartment in TarTC that provides a cellular interaction force at the point when the CTLs are attached.

### G9 deficiency promotes the cytotoxic functions of T cell

Considering that the presence of G9 may change how TarTC physically interacts with CTLs, we assess if this interaction could be replicated in vitro and, in turn, modulate the function of CTLs. We performed co-cultures using human peripheral blood with either NPC43OE6 or C17KD4 cells. For this purpose, PBMC from healthy donors was isolated and pre-cultured in vitro for 2 weeks using the IL2 pre-rapid expansion method (REP). This method allowed continuous T-cell proliferation and viability resulting in an expanded T-cell product (referred to as “ex-T cells”) comprising an average of 93.6% CD3^+^ T cells, of which 38.4% (mean) were CD8^+^ T cells while 20.7% (mean) were CD4^+^ T cells. This enriched ratio of CD8-to-CD4 population in ex-T cells was maintained after 5 days of cocultures (Supplementary Fig 3A). Ex-T cell activation with α-CD3/CD28 stimulation was co-cultured for 72 h with GOF or LOF TarTC. Contrary to previous reports showing that recombinant G9 can induce PBMC cell death [33], immune cell viability was not affected by G9 knockdown or overexpression compared with that of the control cells (Supplementary Fig. 3B, C). In addition, G9 knockdown or overexpression in NPC cells did not have major effect on immune cell proliferation (Supplementary Fig. 3D). These results suggest that G9 negatively regulates TME of NPC in a manner that is independent of immune cell death and proliferation.

**Fig. 3:**
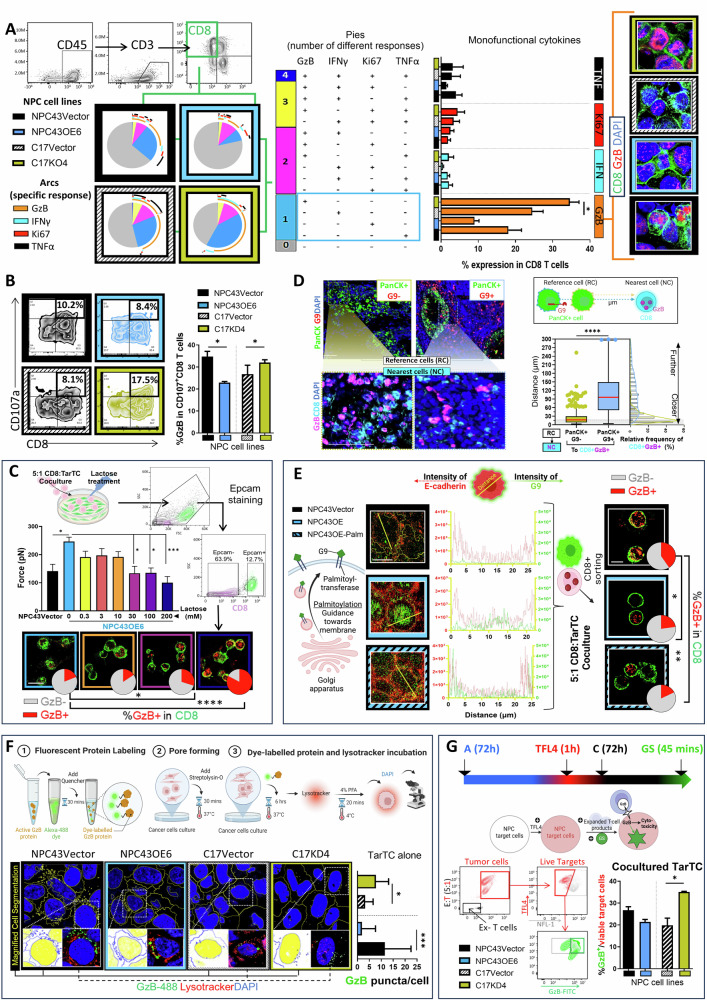
High G9 in tumors affects T-cell cytolytic activities. **A** Mono-(1) Poly- (>1) functional profile of gated CD8 response in G9-knockdown or overexpressed cocultures Pie charts representing the mono-poly-functional profile of gated CD8 response (left) in G9-knockdown or overexpressed cocultures. Size of each pie segment corresponds to the frequency of the corresponding cytokines (color-coded). Arcs depict cytokine makeup within pie slice. Bar graph (right) indicating the percentage of monofunctional profile studied (pie slice in light blue). Representative granzyme B (GzB) staining on cytospun cocultured CTL shown at the side. **B** Representative zebra plot (left) and quantitative plot (right) of CD107^+^CD8^+^ and GzB^+^CD107^+^CD8^+^ cells, respectively. **C** Schematic representation of the workflow for the co-culture system using an anti-EpCAM antibody to distinguish CD8 + T cells. Sorted cells were then cytospun on the side for GzB staining. Pie chart of GzB percentages with the corresponding representative fluorescence staining images. Scale bar: 20 μm. **D** Representative staining CD8 (turquoise), granzyme B (GzB; pink), and nuclei (DAPI, blue) in the tumor region of NPC tissues presented with PanCK+G9+ (*n* = 3) and PanCK+G9− (*n* = 3). The illustration depicts the distance of a cell (PanCK+G9+ and PanCK+G9−) to another cell type (CD8+GzB + ), defined as the distance between the reference cell (RC) and its nearest neighbor cell (NC) of another cell type. Histogram lines represent CD8+GzB+ paired with PanCK+G9+ cells within 300 µm, with shading indicating an effective distance of 15 µm. The percentage of NC at specified distances relative to RC is shown on the right. **E** A schematic showing palmitoylation modification allows G9 to translocate onto the cell surface (left column). Representative confocal fluorescence images of E-cadherin (red) and G9 (green) showing cells with (NPC43OE6-Palm; bottom) and without (NPC43OE6; middle) palmitoylation. NPC43Vector as control. The middle column, line profiles quantifying fluorescence signals from E-cadherin (red) and G9 (green) along the yellow lines in the representative image. Scale bar: 10 μm. The right column, sorted cells were then cytospun on the side for GzB staining with corresponding pie chart of GzB percentages. Scale bar: 5 μm. **F** Schematic illustration of tumor cells loaded with exogenous, activated GzB protein (10 μg) with pore-forming protein streptolysin-O. Endogenous GB puncta Green masks and magnified insets of representative regions staining (left) and quantitative data on GzB puncta in target NPC cells. Scale bar: 50 μm. **G** Schematic representation of GranToxiLux assay. Representative flow cytometry analysis of granzyme B activity (FITC + ) into the TFL4+ TarTC upon cocultures (left) and quantitative data on percentage of death TarTC (GzB-incorporated target cells) (right). The data were pooled from three (*n* = 3) independent experiments. *P < 0.05, **P < 0.01, ***P < 0.001, and ****P < 0.0001 (one-way ANOVA test for (**A**–**C**), (**E**–**G**); two-tailed unpaired Student’s *t*-test for (**D**)). Data points are shown ± SEM

Furthermore, there was no obvious difference in the polyfunctionality of CD8+ and CD4 + T cells (within gated CD8+ and CD4+ from total CD3 + PBMC) between GOF and LOF system compared to that in the control cells (Fig. 3A, left and middle panel and Supplementary Fig 4A). Of note, monofunctional analysis based on intracellular granzyme B released from CD8 + T cells (referred to as CTLs) was significantly increased in the LOF group versus the control group, as detected by flow cytometry and immunocytochemistry (Fig. 3A, right panel). Consistently, the mRNA level of GzB and other transcription factors (i.e., Tbx21 and Eomes) and chemokine receptors (i.e., CCR7), which were closely associated with CTL function [34–36], were more frequently displayed upon knockdown of G9 (Supplementary Fig 4B). Additionally, surface expression of CD107α, which indicates the release of cytotoxic granules [37], was significantly increased in CD8+ in live CD45+ cells in G9 knocking down tumors, while overexpression of G9 exerted the opposite effects (Fig. 3B).

### Tumor-associated G9 impaired T-cell cytotoxic response to tumors is predominantly preceded by cell-cell contact

To evaluate the impact of CTL–TarTC interactions on CTL effector functions, cocultures were performed in the presence of lactose to prevent unspecific binding of G9 to CTL during cell lysis. Lactose, known to disrupt cell surface glycan-based interactions, was utilized to inhibit the formation of CTL–TarTC interactions [38]. Using the cell-cell force interaction approach described earlier, we validated that the force required to disrupt cellular contact in the GOF system (NPC43OE6) decreased with increasing inhibitor doses, indicating lactose successfully dissociation of the cell contact between CTL and TarTC (Fig. 3C). Subsequently, the cocultured CTL underwent sorting and analysis via immunocytochemistry. We observed a notable difference in the percentage of GzB expression among the cocultured CTL under varying lactose treatments. GzB expression was predominantly weak in the CTL fraction treated with low doses of lactose, whereas it was significantly higher in the CTL fraction treated with higher doses of the inhibitor. This effect on impaired CTLs effector function was further supported by our findings from transwell assays, wherein the GOF system, degranulation, and GzB release from CTLs were enhanced when TarTC and CTLs were cultured separately compared to direct co-cultivation (Supplementary Fig. 4C). Therefore, CTL-associated GzB release by G9 critically depends on CTL–TarTC contacts.

In light of these effects on CTL functions, we next tested the physiological meaning of T cells physical contact in TME biology by investigating the spatial distribution of CTL-associated GzB (CTL-GzB) infiltration. Our analysis revealed that CTL-GzB were located in closer proximity to G9-low tumor beds, with a median nucleus-to-nucleus distance of 13.98 µm. In contrast, a significantly greater distance of 32.03 µm was observed between CTL-GzB and tumor cells in G9-high tumors (Fig. 3D and Supplementary Fig. 4D). As mentioned above, while we did not find a correlation between the location of dysfunctional subsets of CD8 + T cells (Tim3 + CD8+ and Tim3-CD8) and G9 expression levels in tumor cells (see Fig. 1H), the GzB-expressing CTLs likely to engage more physically with neighboring tumor cells in G9-low compared to G9-high tumor regions, indicating cell-associated G9 exerts inhibitory effect on the cytotoxicity potential of CTLs.

To test the hypothesis whether membranous G9 expression increased commensurately with CTL functions. We enhanced G9 membrane association in the tumor cells through protein palmitoylation. In Fig. 3E, we demonstrated that G9 is palmitoylated in NPC cells, facilitating its translocation to the membrane with increased interaction with E-cadherin. Interestingly, while overexpressing surface G9 in human NPC cancer cells (NPC43OE-Palm) led to a decrease in the percentage of GzB in the cocultured CTL as compared to NPC43Vector control cells, the GzB percentage remained similar to that observed with coculturing NPC43OE. This result suggests that an increase in expression levels of these cell-surface G9 on TarTC is sufficient to limit CTL effector function, indicating that differences in expression levels (relative expression levels) of G9 on TarTC, rather than their absolute expression levels, are crucial for this process.

To assess further the impact of the observed changes in CTL-associated GzB cells, we analyzed the intracellular GzB content of TarTC. Numerous studies have shown that CTL-associated GzB events can be mimicked in vitro by incubating GzB with cultured cells [39], we assessed the uptake of fluorescently labeled recombinant GzB by NPC cells in the presence of pore-forming protein streptolysin-O (Fig. 3F). The accumulation of recombinant GzB was drastically halted in NPC43OE6 cells, whereas an increased uptake of GzB was detected in G9 KO cells as compared to the control group (Fig. 3F). Furthermore, a GranToxiLux assay, which measures GzB activity in live cells (Fig. 3G), demonstrated a decrease in functionality in G9-overexpressing cells (NPC43OE6) without complete abrogation (Fig. 3G and Supplementary Fig. 4E). Conversely, in G9-knockdown cells, there was an increased functional activity of GzB as compared to control.

Further investigation is needed to determine if this impairment of GzB activities is associated with reduced GzB production in CTLs and decreased GzB uptake in TarTC. Collectively, this evidence suggests that surface G9 may compromise, albeit not entirely, the effector function of CTLs in NPC.

### G9 decreased CTL-mediated tumor cell necrotic death

**Fig. 4:**
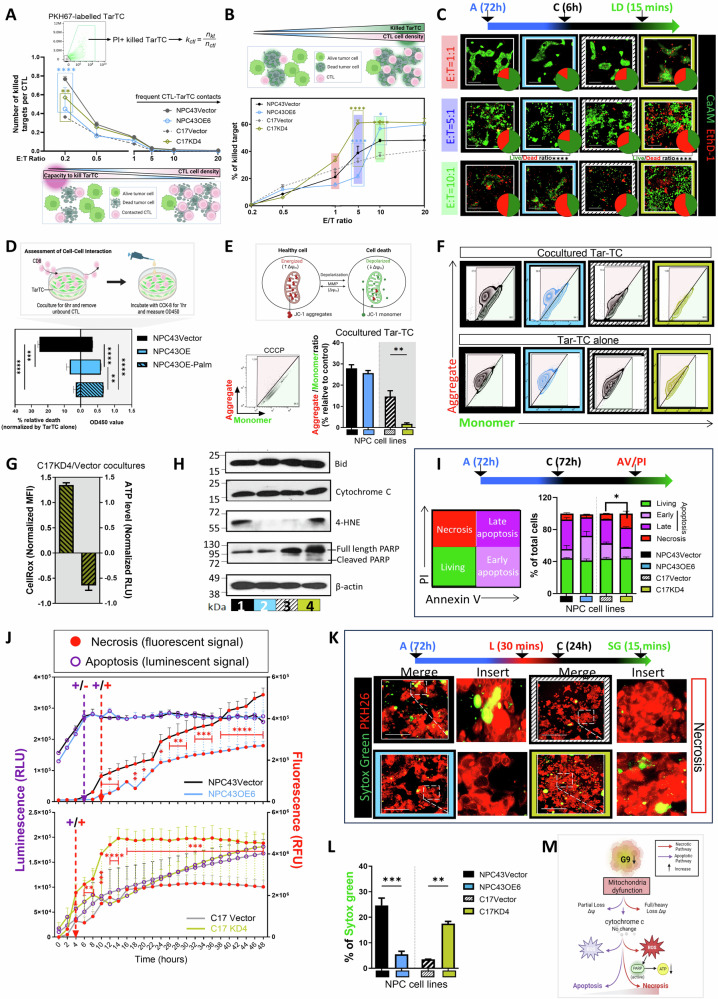
High G9 in tumor cells decreases susceptibility to T cell-mediated lysis. **A** Per capita CTL killing efficacy at the indicated effector-to-target (E:T) ratios. The top row: formula of per capita killing (kctl), where ηkt is the number of target cells killed and ηctl is the number of CTL. The bottom row, illustration depicts CTL – TarTC cell contact types and outcome. **B** Killing efficacy of PKH67-labeled TarTC and cocultured with antiCD3/28 activated Pre-rapid expansion T-cells product (Ex-CD8 + T cells) in dependence of effector-to-target (ET) ratio. Cytotoxicity was evaluated by flow cytometry. The top row, illustration depicts CTL – TarTC cell contact types and outcome. **C** Live/dead staining showed the difference between coculture groups at indicated ET ratio with the corresponding representative fluorescence staining images and pie chart depicting the percentages of live and dead cells. **D** Schematic diagram showing the CTL adhesion assay (top). Two sided bar plot showing relative death normalized to tumor cells assessed by CellTiter-Glo assay and the relative number (OD value) of CTL bound to TarTC quantified using CCK8 assay. **E** Schematic representation of mitochondrial membrane potential disruption assay (ΔΨ) using CCCP treatment as positive control (left) and quantitative data (right). **F** Representative flow cytometry data of JC-1 red-aggregates/green-monomer relative to positive control. **G** Fluorescence intensity of CellROX Green in MFI (mean fluorescence intensity) and ATP in RLU (relative light unit) in cocultured TarTC C17KD4 at 5:1 ET ratio relative to control C17Vector after normalization. **H** Western blot analysis in G9-knockdown or overexpressed NPC cells coincubated with antiCD3/28 activated ex-CD8 + T cells. **I** Flow cytometry analysis of Annexin V and propidium iodide (PI) signals in TarTC. **J** Luminescence (purple, open circle) associated with apoptosis and fluorescence associated (red, circle) with necrosis measured across 48 h of coculture. Data was collected under a bioluminescent, real-time format, involving two independent experiments triplicated for each condition. Events are marked increases in luminescence but not fluorescence (+/−), or increases in both luminescence and fluorescence (+/+). **K** Timeline of the procedure for Sytox green fluorescence staining. A: antiCD3/28 activation of ex-CD8 + T cells; L: PKH26 labeling NPC cells; C: coculture at 5:1 ET ratio; SG: Sytox green staining. Representative images and quantitative data (**L**) of necrotic sytox green-positive TarTC. **M** Simplified scheme of the proposed decision of cell fate between apoptosis and necrosis. In tumor cell presenting with low level of G9, partial or full disruption of ΔΨ may release cellular damage-associated molecule cytochrome C. Sustained high reactive oxygen species (ROS) and ATP depletion favor necrosis, whereas high ATP and low ROS favor apoptosis. The data were pooled from three (*n* = 3) independent experiments. *P < 0.05, **P < 0.01, and ***P < 0.001 (two-way ANOVA test for (**A**, **B**, and **J**); one-way ANOVA for (**D**, **E**, **I**, and **L**). Data points are shown ± SEM

We investigated how CTLs impact tumor growth based on G9 expression levels. The XTT proliferation assay and foci formation analysis showed that altering G9 expression, either through knockdown or overexpression, did not affect cell proliferation. This suggests that changes in G9 levels in TarTC did not impact CTL in controlling TarTC growth through cell proliferation (Supplementary Fig. 5A, B). We then designed an experimental protocol to study killing efficacy during overnight interaction at various effector (E)/target (T) ratios. CTLs were cocultured with PKH67-labeled TarTC (either vector control cells or GOF and LOF cells) and stained with propidium iodide (PI) to track dead TarTC. Cytotoxicity was measured by FACS analysis. In line with previous report [40], we first observed that the per capita CTL killing efficacy was higher at low E/T ratios compared to high E/T ratios. GOF cocultures had a lower number of targets killed per CTL compared to their vector control cells, while LOF cocultures showed the opposite trend (Fig. 4A). Additionally, we observed an increased killing ability of CTLs in G9-knockdown NPC cells, demonstrated by the increased numbers of dead tumor cells (Fig. 4B and Supplementary Fig. 5C) and the quantification of relative live/dead ratio (Fig. 4C and Supplementary Fig. 5D). Conversely, G9 overexpression had the opposite effect, suggesting that G9 may diminish CTL-mediated tumor growth possibly because of abolished cell death. Our analysis also provided evidence that the overall tumor death rate varied with CTL concentration based on the model used. Of note, while killing of TarTC was detectable even at very low E/T ratios (0.5), sustained conjugation (5 E/T ratio) led to G9-overexpressing TarTC (NPC43OE6 cells) exhibiting relative resistance to CTL attack compared to fewer conjugates (1 E/T ratio). Given the robust killing rate was achieved at 5 E/T ratio, we proceeded with a 5 E/T ratio co-culture for further experiments. Supportively, CCK8 assays indicated significantly more CTLs binding to G9-overexpressing tumor cells NPC43OE6 than to vector control cells where this increased binding associated with reduced viability in TarTC (Fig. 4D). Importantly, NPC43OE6-Palm exhibited a notably stronger binding effect than NPC43OE6, without any further significant reduction in viability observed. This serves as further confirmation that the interactions between CTLs and TarTC, initiated by G9 expression, play a crucial role in disrupting CTL-induced tumor cell death, regardless of the level of membranous G9 present.

Altogether, our findings imply effective TarTC cell death depends on frequent contact between CTL and tumor, consistent with previous reports [12].

It has been reported that alternations in mitochondrial membrane potential (ΔΨm) are integral to the cell life–death transition [41]. We, therefore, measured the ΔΨm, disruption of which is an event of both necrotic and apoptotic forms of cell death (Fig. 4E) and noted that the frequencies of JC1 aggregates (ratio of red to green) decreased by 8.6-fold in the cocultured knockdown TarTC samples, compared with those in control vector cells, although comparable aggregational level was displayed between overexpression and vector control samples (Fig. 4F). Importantly, compared with the cocultured NPC cells, cultured NPC cells alone did not show any appreciable difference in ΔΨ by G9 knockdown or overexpression, implying that cell death signals depend on the presence of CTL under our experimental settings. In addition, loss of ΔΨm and production of reactive oxygen species (ROS), as indicated by fluorescent probe of CellROX, showed a close correspondence in the G9-KD cells (Fig. 4G and Supplementary Fig. 5E).

Notably, depletion of intracellular ATP, which is a strict requirement for the execution of apoptosis, was detected in TarTC when G9 is knocked down (Fig. 4G). Among other differences, the switch from apoptosis to necrosis is the consequence of ATP depletion [42]. For this reason, experiments were carried out to compare cell death events under apoptosis and necrosis-inducing conditions. As is shown in the western blot, knocking down of G9 led to a considerable upregulation in the total level of 4-Hydroxynonenal (HNE; biomarker for oxidative stress) and PARP-1 activity (indicator of depleting ATP pools) without affecting the level of Bid and cytochrome c (indicators as pro-apoptosis and cellular damage, respectively; Fig. 4H). Further analysis through flow cytometry indicated that the necrosis rates (as indicated by the annexin-V- PI+ population) of LOF cells were significantly higher in comparison to the control group, while no obvious difference was shown in the apoptotic events (Fig. 4I and Supplementary Fig 5F). Next, we used RealtimeGlo system to evaluate the cell death kinetics of cocultured TarTC cells by monitoring the emergence of the apoptosis marker phosphatidylserine (PS; luminescent signal RLU) and the loss of membrane integrity (fluorescent signal, RFU) over a 48 h period (Fig. 4J). In the GOF system, we observed a rapid increase in PS exposure (indicative of apoptosis) at the beginning of experiment without commensurate increase in the secondary necrosis signal (+/−). By 10 h time point, a plateau was reached in the PS exposure channel, while a significant increase in secondary necrosis was initiated (+/+). Interestingly, NPC43OE6 and its vector control showed minimal necrosis within the first 10 h, whereas C17KD4 and its vector control exhibited necrosis alongside apoptosis as early as 4 h. Notably, necrotic death was reduced under G9 overexpression, while G9 knockdown had the opposite effect on necrosis. We also noted that the percentage of cell necrosis, rather than apoptosis, showed changes over the time-dependent profile. The modulation of necrosis was supported by confocal immunofluorescence microscopy, which highlighted the pronounced SYTOX green uptake in cells with low G9 expression (Fig. 4K, L).

Therefore, our findings revealed that in circumstances where cytochrome c is still released, there is a drop in ΔΨ, generation of ROS, and results in cell death in a fashion resembling necrosis (Fig. 4M). Of note, we observed that culturing NPC cells in the absence of CTL did not affect cell death or ROS production (Supplementary Fig. 5G,H, respectively). This suggests that the majority of induced cell death in NPC cells is associated with CTL contacts, and rare without interaction with CTLs.

Taken together, these data suggest that G9 acts as a controller, hindering tumor cells from undergoing CTL-mediated lysis while regulating cellular processes that contribute to necrosis.

### Cancer-associated G9 triggers autophagy during T-cell-mediated cytotoxicity

**Fig. 5:**
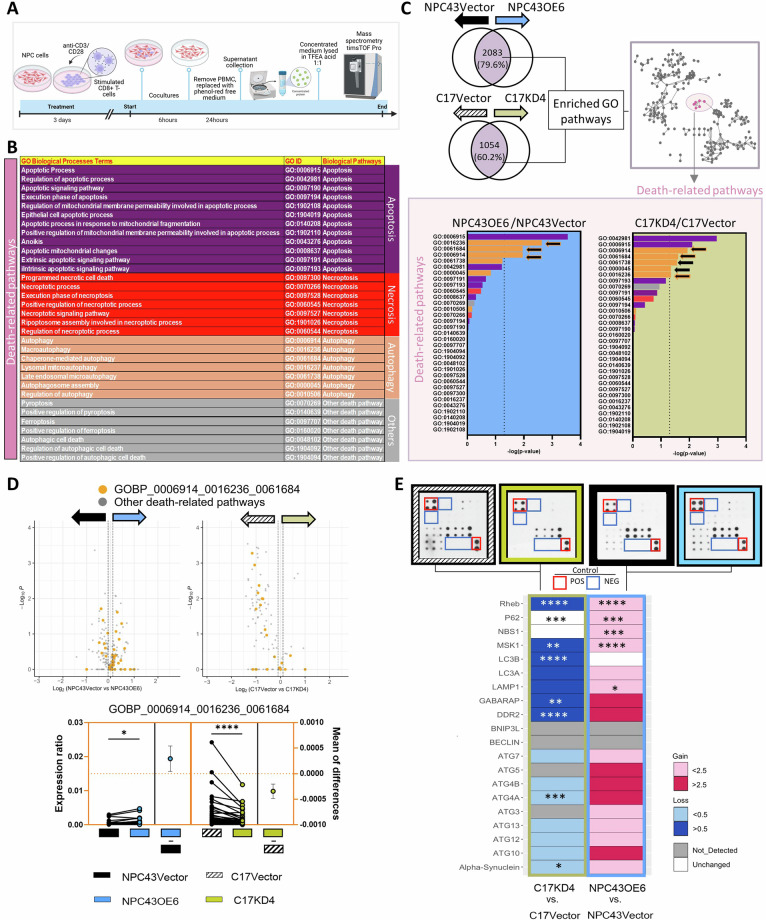
Cancer-associated G9 triggers autophagy-dependent suppression of necrotic cell death during T cell-mediated cytotoxicity. **A** Schematic diagram of proteomics analysis and functional analysis results of cocultured NPC cells. **B** The selected Gene Ontology (GO) terms biological process (BP) associated with associated with The Nomenclature Committee on Cell Death (NCCD). **C** Venn diagram of shared protein detected (above shaded) detected in the condition medium from G9-knockdowned or overexpressed coculture and control groups. Representative Cytoscape network plots of enriched GOBP analysis (right) and BP pathway enrichment analysis ranked based on p values (below). Black arrows indicate the significance of the pathway term related to autophagic process. Black arrows with orange outline indicate common significant BP in each condition. **D** Volcano plots (top) of indicated autophagy BP pathway (orange) and other death-related BP pathways and quantitative Gartner-Aldman plot (below) of expression ratio changes of proteins detected in the selected BP pathways. Data presented as protein expression in G9-knockdowned or overexpressed coculture relative to respective control groups. **E** Proteome Profiler of Human autophagy proteins in cocultured conditioned medium. Quantitation of relative fold induction of cytokines compared with Vector control cells was measured. *P < 0.05, **P < 0.01, ***P < 0.001, and ****P < 0.0001 (two-tailed parametric *t*-test for (**D**) and two-way ANOVA for (**E**))

We performed mass spectrometry (MS) to analyze protein level changes of dysfunctional cell death in the cocultured TarTC after G9 knockdown and overexpression (Fig. 5A). In our MS analysis, we deciphered immune Gene ontology (GO) biological processes (GOBP) associated with The Nomenclature Committee on Cell Death (NCCD) [43] which classified twelve cell death modes based on biochemical and cellular characteristics (Fig. 5B). A total of 6924 proteins were identified, with more than 3000 proteins being identified in every single replicate and an average of 3500 proteins being discovered in each condition. Among them, Venn diagram showed the overlapping proteins were chosen for further analysis (Fig. 5C). GO enrichment analysis results identified proteins that were associated with the NCCD and unexpectedly found that the top 5 significantly enriched cell-death signaling pathways were mainly related with autophagy-related GOBP [44]. We observed significant higher levels of protein classified under GO terms specifically related to the regulation of autophagy in cocultured G9-overexpressing cells compared to control cells. Conversely, knockdown of G9 exerted the opposite effects (Fig. 5D). We further screened key proteins involved in autophagy by a commercially available human autophagy array kit (Fig. 5E) and found that several players in the autophagy machinery such as p62, NBS1, MSK1 were relatively upregulated after G9 was overexpressed. We validated this finding by conducting immunofluorescent analysis, which revealed the formation of autophagosome through the redistribution of microtubule-associated protein 1A/1B-light chain 3 (LC3) from a diffuse distribution to a puncta pattern. We found numerous LC3 puncta in G9-overexpressed tumor cells as compared to vector control. More importantly, double-immunostaining of LC3B and LysoTracker (a fluorescent probe for acidic compartments such as lysosomes) showed that LC3B puncta co-localized with LysoTracker-positive vesicles (Supplementary Fig. 6A). Together, these data indicated the G9-induced autophagy was increased in NPC in response to CTL contact, potentially as a mechanism leading to impaired ability of NPC cells to undergo necrotic cell death.

### Pharmacological blockade of G9-induced autophagy upregulated necrotic cell death

**Fig. 6:**
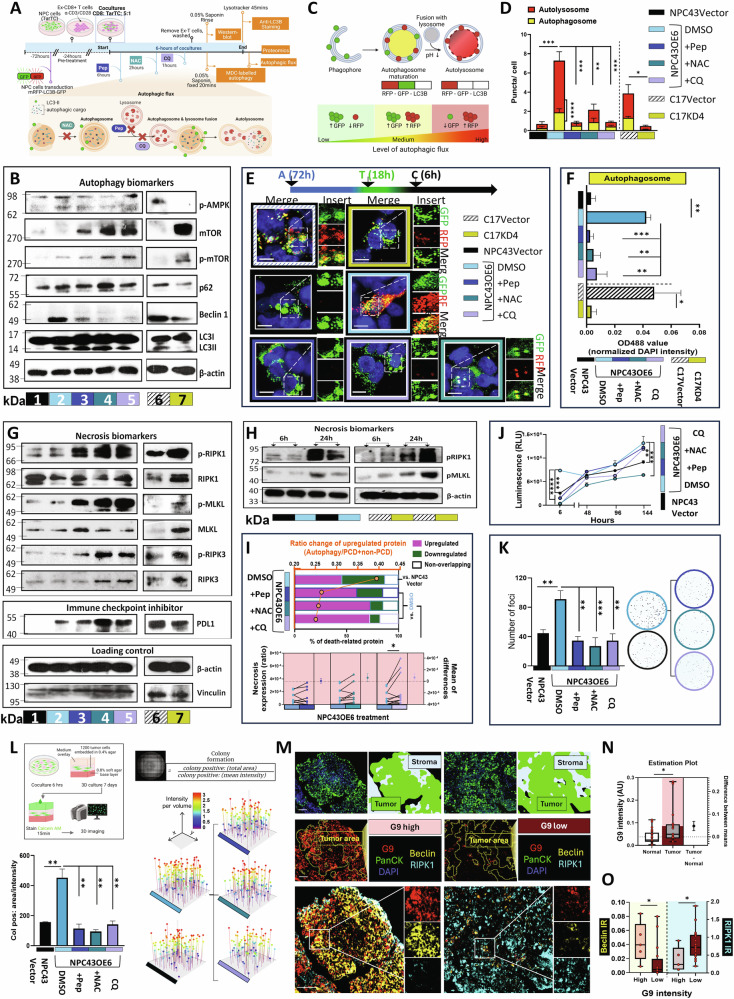
G9 control on the necrotic and autophagic machinery is conserved in NPC patients. **A** Experimental setup: antiCD3/28 activated pre-expanded CD8 + T cells (Ex-CD8 + T cells) cocultured with TarTC. NPC cells with or without transduced with tandem RFP-GFP-LC3B and then independently treated with specific autophagy inhibitors: N-acetylcysteine = NAC (6 uM); chloroquine = CQ (60 uM), pepstatin A = pep (10ug/ml), at indicated time point. Autophagic processes were analyzed through different assays. **B** Western blot analysis on autophagy biomarkers at 6 h cocultures. **C** RFP-GFP-LC3B dual reporter assay to analyze the blockade of autophagy flux. **D** Quantification of autophagosomes and autolysosomes in cell soma. Total number of puncta per region of interest was normalized by number of nuclei. 3 region-of-interest were counted per condition. **E** Representative images of G9-knockdowned or overexpressed NPC tumor cells transfected with the tandem RFP-GFP-LC3B plasmids, following by coculturing with Ex-CD8 + T cells with or without autophagy inhibitors. Cells were then fixed with 4% PFA followed by confocal microscopy. The right parts are enlarged from the boxed areas in the left parts. Autophagosomes denote yellow punta and autolysosomes red punta. **F** Autophagosomes in cocultured TarTC. CTL cocultured with TarTC for 6 h was stained with autophagy-specific green dye and analyzed by microplate reader. Data presented as green signal normalized with blue DAPI signal. **G** Western blot analysis on biomarkers for necrosis and immune checkpoint inhibitor at 6 h cocultures. **H** Western blot analysis on biomarkers for necrosis at 6 and 24 h cocultures. **I** The stacked bar chart represents the percentage (%) of death-related proteins that were upregulated (pink), and downregulated (green) and proteins not overlapped in each treatment group (white). The secondary y-axis (up) shows the ratio (orange dots connected by a line) of proteins from the dataset that map to the autophagy pathway divided by the total number of proteins that map to the programmed cell death (PCD; apoptosis) and non-PCD (necrosis) pathway. Quantitative Gartner-Aldman plot (right) of expression ratio changes of proteins detected in necrosis pathway (GO0070266). **J** Viability assay, **K** colony formation assay, and **L** soft agar colony formation assay (left bar plot: area intensity; right lollipop graphical plot: volume intensity) were employed to determine the tumorigenicity in the presence of indicated autophagy inhibitors. **M** Representative image of the tumor regions, which were selected for G9+, RIPK1+, and Beclin+ cell quantification. Normal tissues (*n* = 5; region-of-interest ROI *n* = 34), tumor tissues (*n* = 9; ROI = 21). Scale bar: 50 μm. **N** Estimation plot on G9 intensity and (**O**) bar plot on immunoreactivity on RIPK1 and Beclin in tumors with high G9 and low G9 expression. *P < 0.05, **P < 0.01 and ***P < 0.001 (one-way ANOVA for (**D**, **F**, **J**, **K**, and **L**) and two-tailed unpaired Student’s *t*-test for (**I**))

An increase in autophagosomes does not necessarily mean that there is an increase in autophagy, as it could also represent a blockade in the process. To further explore the potential impact of autophagy resulting from the interaction between CTL and TarTC, we utilized three pharmacological inhibitors: N-acetylcysteine (NAC), which can scavenge reactive oxygen species (ROS) and inhibit autophagy; chloroquine (CQ) and pepstatin A (pep), which both interfere with lysosomal function of the autophagic process (Fig. 6A). Rapamycin, known for inducing autophagy, served as the positive control in our study. Given the robust autophagy induction observed at 6 h with rapamycin [45], we proceeded with a 6-hour co-culture for further experiments.

We observed upregulated levels of autophagy markers, beclin, LC3BII/I and p62 in GOF group (NPC43OE6) when compared to vector control (Fig. 6B, left blot, lane 2 vs. 1), whereas LOF group (C17KD4) showed the opposite effect (Fig. 6B, right blot, lane 7 vs. 6). Interestingly, upon treatment with above-mentioned inhibitors, G9-induced beclin expression markedly declined, but not the protein levels of LC3-II/I and p62 (Fig. 6B, left blot, lanes 3, 4, 5 vs. lane 2). Both lipidated LC3-II and lipidated p62 protein are selectively degraded during autophagy. An increased accumulation of both of these proteins points toward a deficiency of lysosomal-dependent degradation of the autophagosome, and thus their contents are retained, and accumulation of the proteins is expected [46]. Indeed, we observed when NAC was added along with the cocultures, the levels of LC3II/I and p62 were higher as compared those effects observed by the addition of lysosome blockers (CQ and Pep).

Because AMPK signaling plays an important role in the regulation of cellular autophagy, especially during the autophagosome elongation stage [47, 48], we next investigated the association between AMPK and G9-associated cancer cell death in response to CTL. We found when cocultured CTL with G9-deficient TarTC cells, the activated form of AMPK (Fig. 6B left blot, lane 1 vs. 2; right blot, lane 6 vs. 7) was lower. When autophagy was pharmacologically inhibited, the induced levels of activated AMPK were slightly reduced in G9-overexpressing TarTC (left blot; lanes 3, 4, 5 vs. lane 2). Furthermore, such reduction of AMPK was negatively correlated with p-mTOR expression. (Fig. 6B, left blot; lanes 3, 4, 5 vs. lane 2). As before, knocking down or overexpressing G9 in NPC cells exhibited similar protein expression of the autophagy signaling cascade in the absence of CTL, indicating that the upregulated autophagy in TarTC is CTL-associated (Supplementary Fig. 6B).

To further verify whether the elevated autophagic activation by G9 is preceded by increased autophagy induction or inhibition of autophagosome and lysosome fusion, we studied the “autophagic flux”. This activity can be morphologically traced with an mRFP-GFP-LC3 tandem construct, where autophagosomes and autolysosomes are labeled with yellow (RFP+/GFP+) and red (GFP-/RFP+) signals, respectively (Fig. 6C). The results for the cocultured TarTC expressed with high level of G9 showed an increased number of both yellow and red puncta as compared than control group, indicating that there is an ongoing autophagic flux (Fig. 6D, E). After treatment with autophagosome formation inhibitor (Pep) and an autophagosome-lysosome fusion inhibitor (CQ), we found G9-induced autophagic flux was drastically reversed in the cocultured G9-expressing TarTC. It is also worth noting that NAC alone was not potent enough to reduce the autophagosome-lysosome fusion as CQ and Pep, indicating that autophagic machinery cannot be reversed by simply reducing the intracellular ROS. Nevertheless, the addition of all of the three autophagy inhibitors could completely suppress G9-induced autophagic vesicles in response to CTL (Fig. 6F), as detected by reduced MDC green signal. As before, the increases in the autophagic activities were unlikely to be associated with intrinsic G9 expression in tumors, as evidenced by the similar MDC levels between G9-depleted/overexpressed and vehicle-control NPC cells in the absence of CTL (Supplementary Fig. 6C).

Although factors other than ROS contribute to the G9-induced autophagy, the level of intracellular ROS can partially determine the fate of the cell. It has been reported that low levels of ROS can induce programmed-cell death (PCD) while accumulation of high levels promotes necrosis or can lead PCD-committed cells toward necrotic-like destruction [49]. We have proven that knockdown of G9 increased the generation of ROS in the previous section, thus, it is reasonable to hypothesize that the upregulation of G9 in NPC cells is involved in impeding necrotic tumor cell death. As expected, G9 deficient TarTC (i.e., C17KD4) when cocultured with CD8 + CTL, had a higher expression of receptor-interacting protein kinase-1 (RIPK1), activities of RIPK1 partner, RIP3 (RIPK3) and mixed lineage kinase domain-like (MLKL) when compared to the control vector cells (Fig. 6G, right blot, lane 7 vs. 6).

As shown in Fig. 6G, no significant changes in necrotic-related biomarkers expression were observed in G9-overexpressed TarTC during a 6 h coculures (left blot; lanes 1 vs. lane 2), possibly indicating a delayed onset of necrosis at 12 h after coculture (see Fig. 4J). Following pharmacological treatment, there was an upregulation of RIPK1/RIPK3/MLKL expression on the cocultured G9 overexpressed TarTC (Fig. 6G, left blot; lanes 3, 4, 5 vs. lane 2), while no such effect was observed in the cocultured control vector TarTC (supplementary Fig. 6D). These results imply that G9-induced autophagy may limit necrosis, yet it may not completely elucidate G9’s role in regulating damage caused by CTLs in TarTC. Subsequently, to validate whether G9 expression regulates CTL-induced damage in NPC, we carried out similar coculture experiments with an extended time point of 24 h. Western blot analysis revealed a time-dependent effect of necrosis activation under G9 expression (Fig. 6H), with a more pronounced expression of necrosis-related markers at 24 h compared to the 6 h time point. This activation of necrosis was significantly reduced in G9-overexpressing TarTC. Conversely, a reverse trend was observed in the LOF system, with a remarkedly induction of necrosis in co-cultured G9-deficient TarTC (C17KD4) at 24 h. Taken together, these results suggest that autophagy is instinctively induced prior to necrosis, and the necrotic status of TarTC depends on tumoral-G9 expression.

Of note, another immune checkpoint, PD-L1 has been reported to be intrinsically upregulated in response to autophagy in gastric cancer [50]. We found that the expression of PD-L1 was likely associated with the level of G9 and shared similar expression profiles with slight upregulation upon the addition of autophagy inhibitors (Fig. 6G, left blot, lane 2 vs. 1 and right blot, lane 7 vs. 6). Nevertheless, our results indicate that G9 regulate the response towards CTL-mediated killing at least in part via promoting the occurrence of autophagy, and thus fail to kill NPC cells via reduced necrosis. This statement was validated via the MS analyses and confirmed that the level of autophagy was higher than the PCD and non-PCD in G9-overexpressing TarTC, where the latter process attributed to necrosis was significantly reduced upon autophagy blockade (Fig. 6I).

### Cell-associated, rather than cell-derived, G9 cooperates with CTL to trigger autophagy-related cell death disruption

It is intriguing to note that while solid tumor cells typically do not secrete G9 under normal conditions [51], the interaction with T cells can stimulate G9 secretion in other solid tumor cells, likely facilitated by autophagy and subsequent lysosomal secretion [52]. Given this context, we aimed to investigate whether the autophagy induction observed in our experimental setup during the interactions between NPC and CTL could be reduced by neutralizing the soluble form of G9. To address this, we treated the co-cultures with various doses of G9 inhibitors. G9 neutralizing antibodies had no effect on G9-induced tumor cell death where there no restoration of autophagy in the GOF and LOF system, as well as necrosis (supplementary Fig. 6E). Therefore, we posit that the cell-associated membranous form of G9, rather than the cell-derived soluble form, plays a pivotal role in maintaining the autophagy status of tumor cells.

### Cell-associated G9 interacts with CTL promote tumor growth by autophagy induction

We explored the potential of tumor growth promotion resulting from G9-induced autophagy during CTL–TarTC interaction. We conducted CellTiterGlo and colony formation assays. The proliferation of co-cultured NPCOE6 induced by G9 was notably decreased in the presence of autophagy inhibitors (Fig. 6J). Similarly, we observed a decrease in the number of colonies formed by co-cultured NPCOE6 upon inhibition (Fig. 6K). Anchorage-independent growth is a critical characteristic of cellular transformation and serves as a reliable in vitro method for detecting malignant cell transformation [53]. Therefore, we investigated the impact of G9-induced autophagy on the tumorigenic activity of NPC43OE6 using a soft agar colony formation assay (Fig. 6L). NPCOE6 cells were grown in soft agar for 7 days, and the area and volume of colonies were quantified using Imaris software. The overexpression of G9 led to a significant increase in anchorage-independent growth, which was diminished upon autophagy inhibition. The reduced tumorigenicity was associated with an increase in necrosis, and necrotic cell death could be restored after autophagy inhibition (Supplementary Fig. 6F).

### The reduction of necrotic cell death by G9-activated autophagy is clinically relevant

The data presented thus far indicated an unreported G9-mediated escape from CTL cytotoxicity via upregulation of autophagy to reduce necrotic cell death.

To validate these findings, we performed multiplexed staining on human tumor tissues and analyzed the results using an established quantitative approach to evaluate immunoreactivity (IR) within the tumor cells. Using a cell intensity-based measurement, the median [range] staining intensity (in arbitrary unit, AU) of G9, obtained from at least two region-of-interests (ROIs) from same patients, was significantly higher in tumoral regions (0.049 [0.016–0.282]) than normal regions (0.024 [0.003–0.113]) using PanCK-guided cell segmentation (QuPath’s built-in algorithms; Fig. 6M, N). The tumor samples were divided into two groups based on the mean value for G9 intensity in tumor cells (0.086), resulting in G9 low and G9 high groups. To calculate IR based on RIPK1 and Beclin expression, which is the number of Beclin/RIPK1-positive staining cells (in tumor region) divided by the total number of tumor cells (DAPI), multiplied by staining intensity, was used (Supplementary Fig. 7A). In Fig. 6O, we found that the G9 high group exhibited high Beclin IR (0.040 [0.009–0.084]) and low RIPK1 IR (0.237 [0.096–0.905]). Conversely, the G9 low group showed the opposite trend (Beclin IR: 0.004 [0–0.080]; RIPK IR: 0.717 [0.192–1.878]). We therefore propose that the autophagic status of target cells plays a key role in the resistance to CTL-mediated lysis in NPC, particularly in tumors enriched with G9 expression.

To robustly confirm the above findings, malignant epithelial cells were stratified into four categories based on UCell score of necrotic cell-death pathways and proteolytic pathway gene signatures (Supplementary Fig. 7B). High rate of both necrotic cell-death and proteolysis score, implying a strong necrotic response of cell, could be detected in malignant cells with lower autophagy score and G9 gene expression (Supplementary Fig. 7C). We next examine the association between CTL activities (based on GzB expression) and the levels of autophagy and necrosis in tumor tissues from NPC patients based on the GSE150430 database.

To associate T-cell subsets with cytotoxicity, tumor-infiltrating CTL clusters that were identified based on expression levels of 33 genes related to cytotoxicity (including *IFNG*, *PRF1*, *GZMA*, and *GZMB*) within CD8+ clusters were further used for patient stratification. As indicated in Supplementary Fig. 7D, patients were stratified into the GzB-high (*n* = 5) and GzB-low (*n* = 6) groups based on the level of GzB-expressing tumor-infiltrating CTLs (median value of average expression as the cut-off point). We therefore performed a differential Pearson correlation analysis and compared on necrotic and autophagic related gene patterns in GzB-high versus GzB-low group (Supplementary Fig. 7E). The results of this analysis revealed that two positive associations were conserved between the two population groups, between MAP1LC3B/RIPK1 and BECN1/MTOR. Two distinct directional changes were observed in GzB-high population. These changes included a negative association between RIPK1/LGALS9 (G9) and BECN1/ MAP1LC3B (Spearman correlation coefficients of −0.78 and −0.89, respectively; both *p* < 0.05), which was either lost or become positive association in the GzB-low patient group. The former correlation supports the above hypothesis that patients with more “effective” (GzB-high) CTL are well correlated with necroptosis-related events (RIPK1) and were inversely correlated with the expression of G9. The latter correlation also confirms our speculation that patients with “ineffective CTL” (GzB-low) may have more autophagic-related genes. Of note, the detection of MAP1LC3B does not distinguish between LC3-1 and LC3-II which may not directly reflect autophagic activities in NPC specimens. However, the strong positive association in MTOR/RIPK3 in the GzB-high population, which was lost in the other group, indicates an interconnection among autophagy, necroptosis, and CTL effector functions in NPC patients.

Taken together, our analysis renders a picture of alternations in associations between autophagic and necrotic levels in NPC tumors upon exposure to CTL, which seem to be influenced by the presence of G9.

### Targeting autophagy and G9 potentiates response to immunotherapies and effectively suppresses tumor growth in vivo

Because we show that G9 directs autophagic/necrotic signaling upon engagement with CTL in the current study, we selected ACT, ICB or autophagic blockade for combination therapy with G9 in three in vivo mice models.

**Fig. 7:**
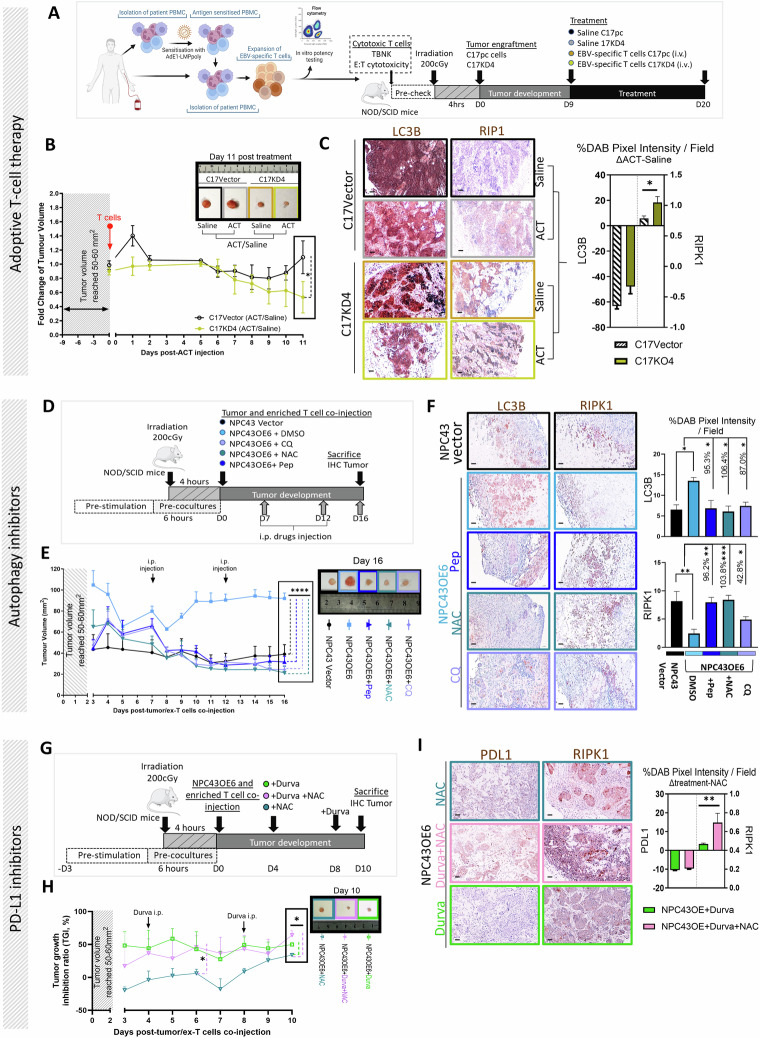
Enhanced suppression of tumor growth in response to immunotherapies through targeting autophagy and downregulation of G9. **A** Experimental setup: Process for generating EBV-specific T cells using AdE1-LMPoly for adoptive transfer into mice engrafted with HLA-matched G9-knockdown or vector tumor cells. The phenotypic (TBNK assay)/functional (cytotoxicity) characteristics of T cells were assessed before transfer. Day 9 (D9) post tumor cell inoculation, mice were adoptive transferred with EBV-specific T cells (ACT) intravenously (i.v.). *n* = 5 mice per group. Control mice received saline. **B** Representative images of tumors at the last day of the experiment and tumor development over time. Fold change in tumor volume treatment/control group. **C** Representative images (left) and quantitative data (right) of immunohistochemical staining. Delta change pixel intensities (treatment-control). **D** Experimental group: antiCD3/28 activated pre-expanded CD8 + T cells (Ex- T cells) cocultured with NPC cells with indicated autophagy inhibitors before co-injection. **E** Representative images of tumors and tumor development over time. P was calculated relative to NPC43OE6. **F** Representative images (left) and quantitative data (right) of immunohistochemical staining in tumors. **G** Experimental group: antiCD3/28 activated pre-expanded CD8 + T cells (Ex- T cells) cocultured with NPC cells before co-injection with or without durvalumab. **H** Representative images of tumors and tumor development over time. *P* was calculated relative to NPC43OE6 + NAC. **I** Representative images (left) and quantitative data (right) of immunohistochemical staining. All quantitative analysis of staining was calculated from 3 region-of-interest counted per slides; scale bars = 50 μm; 20x magnification). *P < 0.05, **P < 0.01, and ****P < 0.0001 (Mann–Whitney test for (**C**, **I**), two-way ANOVA for (**F**)). Tumor growth was compared by mixed-effects analysis followed by Bonferroni’s multiple comparisons test *n* = 3–4 mice per condition

We first hypothesized that G9 depletion would improve the therapeutic effect of allogeneic HLA-matched EBVSTs in our model. To address this hypothesis, we generated AdE1-LMPoly-T-cell products comprised with 95.4% CD3 + T cells, of which 29.7% were CD8^+^ T cells while 68.8% were CD4^+^ T cells (median; Supplementary Fig. 8A). These characteristic immune subpopulations are consistent with the findings of our in vitro data (Supplementary Fig. 3A), indicating that T cells under REP protocol are enriched with CD8+ subpopulations. Notably, this EBVSTs product not only retained functional EBV antigen specificity (Supplementary Fig. 8B), but also exerted high in vitro cytolytic activities towards G9-knockdown NPC cells when compared to control cells (Supplementary Fig. 8C). Mice engrafted with C17KO4 or vector control tumor cells were treated with allogenic EBVSTs as indicated (Fig. 7A). As expected, ACT with EBVSTs treatment was effective in reducing tumor size of G9-depleted mice when compared to control vector cells (Fig. 7B) and showed that the depletion of G9 indeed increased the necrosis (RIPK1) and decreased autophagy (LC3B) by 3.1- and 1.32-fold, respectively (Fig. 7C).

Consistent with this line, we further hypothesize that inhibiting G9-induced autophagy in response to CTL exposure could reduce tumor growth. For that, we imitate the above in vitro mechanical finding to pre-coculture ex-CD8 + T cells with tumor cells for the initiation of autophagic process in tumor cells before mice engraftment (Fig. 7D). As shown in Fig. 7E autophagy inhibitors-treated mice bearing G9-overexpressed tumors showed a delayed onset of tumor growth compared to the vehicle treatment group. Additionally, tumor size was significantly reduced upon autophagy inhibition compared to untreated mice, reaching levels similar to or even lower than basal levels in mice not injected with G9-overexpressing cells (i.e., NPC43Vector). This suggested that cell-intrinsic autophagy may impact tumor growth. A similar trend was observed in the immunostaining for RIPK1 and Beclin, where increased expression of necrotic markers in tumor tissue was noted under controlled autophagy activation compared to control mice (Fig. 7F). These findings support the notion that G9-induced autophagy within cells can suppress tumor cell death, thereby promoting unhindered tumor growth.

ICB by PD-1 or PD-L1 antibodies can reactivate T-cells [54]. We hypothesized that the autophagy induced by G9 in response to CTL could impact the efficacy of ICB (Fig. 7G). A slightly stronger inhibitory effect on tumor size was observed upon treatment in combination with autophagy inhibitor and durvalumab (a PD-L1 inhibitor) as compared to monotherapy with durvalumab (Fig. 7H). The dampening effect coincided with reduction of autophagy-mediated necrosis (Fig. 7I). Thus, immunosuppressive TME mediated by autophagy activation promotes tumor progression in vivo.

These results demonstrate that reducing G9 expression on tumor cells, particularly under conditions of limited autophagy, restores CTL-mediated lysis and enhances the response to IMT in necrosis-refractory tumors.

**Fig. 8:**
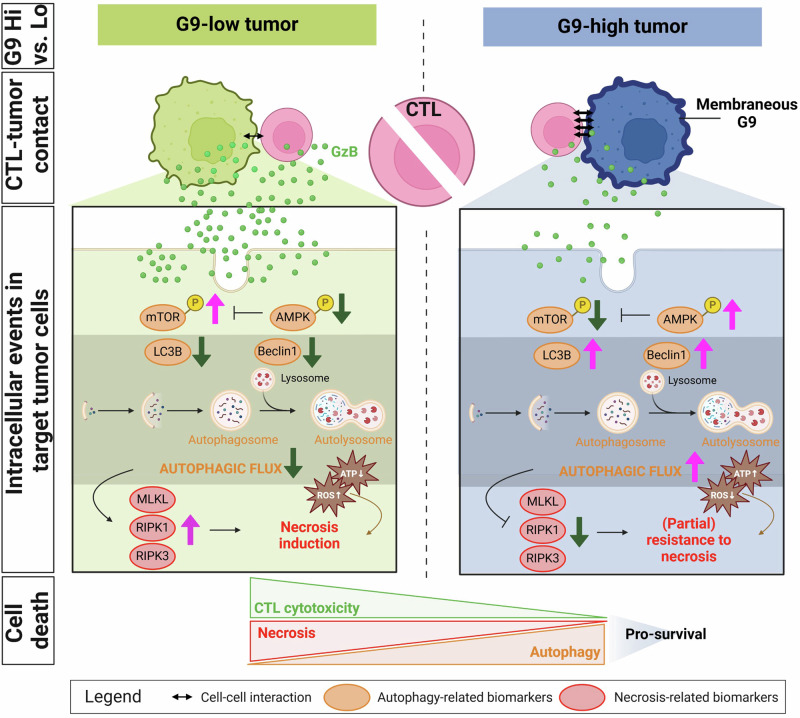
G9 overexpression promotes resistance to CTL-mediated cell death in tumors Image summarizing the main study findings. When cytotoxic T cells (CTLs) bind to G9 protein overexpressed on the surface of tumor cells, it leads to a reduction in granzyme B (GzB) release from CTLs. This binding also causes an activation of AMPK and inhibition of mTOR. These changes result in an increase in autophagy flux where Beclin and LC3B expression are upregulated. As a result, the tumor cells become resistant to necrotic cell death (MLKL, RIPK1, RIPK3). Autophagy and necrotic cell death are interconnected and may share common underlying molecular pathways involving CTL-mediated killing

Overall, our findings suggest that while cell-associated G9 does not completely undermine the effector functions of CTLs, the autophagic status of tumors is significantly heightened due to the interaction between CTLs and NPC-associated membranous G9. This leads to resistance against CTL-mediated lysis and impairs tumor damage. We propose that cell-associated G9 primes cells for autophagy and operates as an intrinsic resistance mechanism in tumor cells (Fig. 8).

## Discussion

Despite the presence of CTLs infiltrating the TME in cancer patients or those undergoing ACT, the mechanisms underpinning the defective killing effect on tumor cells remain uncertain. In our study, we found that most NPC cells expressed a membrane-bound form of G9, and a significant amount of CD8+ cells were located within 15 µm of these G9-positive tumor cells. This close proximity suggests that CD8 + T cells may exert their function through direct contact with tumor cells, indicating the possible existence of functional niches in this region. These niches may contain cytotoxic granules such as GzB, which are known to induce TarTC death. Interestingly, when CTLs interacted with G9-overexpressing NPC TarTC, we observed a decrease in the production of GzB by the CTLs, while the activation of GzB in TarTC was not completely inhibited. This interaction instead led to disruption of the tumor cell death machinery, characterized by an increase in autophagy and a decrease in necrosis. These findings strongly suggest that G9 plays a crucial role in regulating the damage caused by CTLs in NPC. Importantly, inhibition of G9 expression is sufficient to counteract resistance to cell death and to increase sensitivity of tumor cells to ACT or IMT. Therefore, we have uncovered a potent immuno-oncogenic property of G9 in cancer, characterized by its ability to promote immune evasion, tumor survival, and resistance of the tumor to immune-mediated cell death.

We observed Tim3, which is known to interact with G9, was relatively low on T-cell infiltrates compared to other counteracting receptors. Furthermore, Tim3 expression level was not strongly correlated with the expression of G9 in NPC patient specimens. This suggests that G9 may facilitate T-cell function independently of Tim3 [55]. Our spatial staining revealed a correlation between clinical survival outcome and the spatial density of CD8 + T cells, as well as the level of G9 expression in the tumor subregion. We observed that a high density of CD8^+^ T cells infiltrating with low expression of G9 in tumor cells was associated with improved patient survival in our cohort. However, in contrast, even when there were high levels of CD8^+^ cells infiltrating the G9-high region, patients exhibited poorer survival outcomes. This spatial feature, along with the close proximity CTLs to tumor cells, was consistent with the density of CTL-derived GzB-expressing CTLs in the tumor mass. Therefore, we provide the first indication that, in addition to density of immune infiltrates, their spatial context based on the proximity of CTLs to G9-expressing tumor cells is a crucial factor in creating an “effective” functional TME.

Gs are known for organizing plasma membrane components through glycan-mediated interactions. Among them, G9 stands out for its dual nature, acting as a “double-edged sword” depending on where it is located within cells. Intracellular G9 can stimulate the expression of pro-inflammatory cytokines in monocytes [56] and drive actin polymerization in dendritic cells [57], while extracellular G9 can induce monocyte death. Similarly, cytoplasmic G3 has been reported to protect against apoptosis by interacting with the mitochondrial membrane to maintain its integrity and prevent the release of cytochrome c, whereas extracellular G3 triggers T-cell death directly [58]. This highlights how the localization of Gs can lead to distinct functions. Very little is known regarding the function of membranous G9, although it has been implicated in impaired T-cell phenotypes [59]. Our findings introduce a novel concept where membranous G9 on tumor cells interacts with CTLs, reducing the susceptibility of TarTC to CTL-mediated lysis. Disrupting CTL interactions with G9-expressing tumor cells may therefore improve CTL-mediated cell death. Lactose or its derivatives, along with functionally equivalent compounds, may hold promise in this regard. Recent research has linked the upregulation of G9 by anthracyclines to tumor immune escape, with a combination therapy involving Adriamycin and anti-G9 treatments showing potential in cancer therapy [60].

We note that membrane localization of G9 could offer a new therapeutic target, and that modulation on the transport of G9 from the cytosol to the cell surface may impact its function. We propose that intracellular G9 is translocated to the plasma membrane to replenish the pool of membrane-bound G9, thereby enhancing resistance to CTL-mediated tumor cell death. Post-translational modifications, such as O-GlcNAcylation, has found to regulate the translocation of proteins. We, therefore, posit that, rather than targeting G9 with single-epitope antibodies, designing molecules that target enzymes involved in O-GlcNAcylation and G9 in PanCK-positive tumor cells could be more effective. This approach allows for specific targeting of G9 antigens expressed in tumor cells while minimizing disruption to cellular homeostasis. Novel strategies utilizing protein-specific O-GlcNAcylation targeting chimeras have emerged [61], offering potential for targeted therapies in G9-highly presenting NPC. This approach may reduce “on-target/off-tumor” side effects while effectively targeting G9 antigens.

To evaluate the impact of CTL-tumor pairs, we studied the status of TarTC on the selection of cell death. To date, the interlink between autophagy and necroptosis is currently not well understood, with conflicting findings and controversies regarding their interconnection. Our data now support a simultaneous decrease in necroptosis markers and an increase in autophagy-related proteins driven by CTL-tumor conjugations under conditions of high G9 expression. We also reported statistically significant negative association between RIPK1 and Beclin expression in NPC tissues with high levels G9 expression, confirming the clinical relevance of the pro-survival function of autophagy in tumors is restricted to necrosis.

Our result supports the idea that autophagy can prevent necroptosis, as shown by others [62] and that this effect can be reversed by relieving mTOR signaling inhibition [63], highlighting the protective role of autophagy in promoting cell survival against necroptosis. However, some studies have suggested that autophagy promotes necroptosis [64, 65], where the generation of ROS triggered by autophagy is considered as one of the initiators of necroptosis [66]. On the other hand, autophagy has also been reported to be induced by necroptosis [67]. Therefore, understanding the directional modulation between cellular autophagy and necroptosis can fine-tune the effectiveness of CTL-mediated killing upon detection of immunosuppressive membrane proteins on tumor cells, thereby sustaining an “effective” cancer immunity. Moreover, comparative analyses of G9 expression in NPC patient samples with varying degrees of CTL infiltration could offer clinical correlations that strengthen the evidence supporting the involvement of G9 in modulating CTL-induced damage.

Our study establishes the presence of G9 on the plasma membrane of TarTC as a key factor in defining cancer immunity. By assessing G9 surface expression, we can discern the interaction between TarTC and CTLs, leading to autophagy induction over sublethal necrosis. Interestingly, a recent finding by Schlichtner et al. [52] has shown increased G9 secretion in response to their interactions with T cells in several cancers, including breast cancer cells, Wilms tumor cells, kidney rhabdoid tumor cells, and glioblastoma cells. This secretion is triggered by the translocation of G9 to the cell surface via proteolytic shedding or autophagy followed by lysosomal secretion using Tim3 as a protein carrier, as G9 lacks a secretion sequence. Our experimental data from pharmacological inhibition using a G9 neutralizing antibody revealed that blocking G9 failed to restore necrosis and reduce autophagy, suggesting that cell-cell interactions, rather than secreted G9, dictate the choice of cell-death pathways in TarTC. In the context of NPC, our previous findings showed that more than 60% of NPC cases exhibited a scarcity of T cells within tumor areas categorized as “immune-excluded” profiles [68]. Building on this, we now reveal that Tim3-expressing cells within a distance of 15 μm from the effector site are also scarce in NPC tissues. The scarcity of Tim3 may suggest inadequate G9 secretion during T cell-malignant cell interactions, which could lead to the maintenance of an autophagic status in TarTC through G9 that is not rejuvenated by G9 neutralizing antibody blockade.

Distinct staining patterns of G9 in cervical and vulvar squamous cell carcinomas, with robust expression in both the nucleus and cytoplasm despite membrane staining have been reported [69]. This suggests that G9 may play varied roles in shaping different tumor immune microenvironments, functioning as an “independent entity”. It is essential to study G9 in different cancers as it exhibits distinct functions. It is worth noting that the translocation of G9 from the cytosol to the membrane on T cells post-activation is associated with impaired phenotypes [59]. This raises questions about the role of G9 upregulation on T cells in disease progression versus its potential as a protective host mechanism to prevent hyperimmune activation-induced collateral damage in cancer contexts. Understanding the relative contributions of G9 in T cell dysfunction and autophagy promotion in TarTC within the complex ecosystem is crucial. Future studies should focus on exploring strategies to prevent G9-induced autophagy disruption of TarTC death while simultaneously reducing G9 upregulation on T cells, which could potentially enhance the effects of immune checkpoint blockade.

In conclusion, our data suggests that the interaction between CTL and the cell-surface G9 protein on TarTC is crucial in triggering cancer cell death and enhancing their sensitivity to IMT. These findings highlight the potential for targeted IMT or a combination of complementary treatment modalities to disrupt inhibitory cell-cell contacts that hinder immune effector-cell collaboration, thereby restoring efficacy of immune-based therapies.

## Patients and methods

Descriptions of cell lines, primary human cells/samples, expanded methods, and additional information can be found in the Supplementary Information. The HLA types of these cell lines are listed in Supplementary Table 1. A summary of the EBV antigen specificity (HLA restriction) of transferred T-cells is shown in Supplementary Table 2.

### Creation of stable cell lines

Overexpression of G9 in NPC43 cells was performed by transfection of G9 construct in pReceiver expression vector (GeneCopoeia) by lipofectamine 3000. Stable clones were obtained by hygromycin selection (30 µg/ml) for 2 weeks and maintained in medium containing 30 µg/ml hygromycin. Knockdown of G9 performed using Gal-9 shRNA targeting human LGALS9 gene and a negative control shRNA (GeneCopoeia). Briefly, 293 T cells were transfected using Lipofectamine 3000 with recombinant lentiviral particles. Viral supernatant was collected at 48 h after transfection and filtered for subsequent experiments. Cells were infected with viral supernatant containing polybrene for 48 h and selected by incubation with puromycin (2 μg/ml) for 2 weeks. Loss or gain of targeted proteins was confirmed by Western blot.

### Palmitoylated and its control cell lines generation

The generation of palmitoylated cell line was conducted with methods previously reported [70]. Descriptions of palmitoylation are given in the online supplementary information.

### Coculture assays

Tumor cells were seeded into culture dishes (24-well for FAC analysis and immunocytochemistry, ICC, and 6-well for Western blot) at 30% confluency, and grown into a ∼70% confluent monolayer 18 h later. The PBMC pre-stimulated with anti-CD3/CD28 (Miltenyi Biotec) for 24 h were pelleted by centrifugation at 300 × *g*, resuspended in the RPMI medium, laid on top of the monolayer tumor cells (cell number of PBMC/tumor cells = 5:1) and cocultured for 3 days. For contact-independent experiments, PBMC was added to a 0.4 µm pore-sized Transwell insert placed on the wells. For FAC analysis, the non-adherent PBMC were gently rinsed away by two washes with PBS for subsequent staining. For Western blot and ICC, PBMC was pre-stimulated for 3 days and cocultured with tumor cells for 6 h. Where indicated, three drugs were added subsequently at different time points before collection: 6 h for 10ug/ml Pepstatin, 6 uM N-acetyl-l-cysteine (NAC) for 2 h or 1 h for 60 uM Chloroquine (all from MedChemExpress). In some experiments, coculture systems were incubated with lactose (MedChemExpress) and anti-galectin-9 antibody, neutralizing (MERCK) for 6 h.

### Cell-cell interacting force study

Isolated CD8 + T cells were pre-stimulated with anti-CD3/CD28 (Miltenyi Biotec) for 72 h. Stimulated cells were cocultured with tumor cells in suspension for either 24 or 72 h before taken out for force study. Cells were placed on a microscopic slide to identify interacting tumor-CD8 + T cells. CD8 + T cells were trapped in optical tweezer (m-Trap, LUMICKS) to be detached from tumor cells and the interacting force was recorded. The 1064-nm trapping laser beam was focused inside the sample chamber by a 60× water immersion objective with a 1.2 numerical aperture (N.A.) to trap cells. The optical trapping force measurements were calibrated by the built-in force calibration system in the optical tweezer.

### Tumor cell-adhesion assay

Tumor cells were prelabelled with PKH67 green fluorescent Cell Linker and pre-stimulated sorted Ex-T cells were prelabelled with PKH26 red fluorescent Cell Linker (both from Invitrogen). Both cell groups were then co-cultured for 1 h at 10:1 CD8:tumor cell ratio in Falcon round bottom polypropylene tubes. 2% PFA fixation was added to the mixture prior to centrifugation. Cells were then analysed by flow cytometry or deposited onto glass slides via Cytospin apparatus using a centrifuge set to 350 × *g*. The cells were fixed onto the glass slide with a drop of ice-cold methanol. Once dry, the sample was analyzed via Confocal LSM900 microscope.

### Cell viability, ATP, and cell death assay

Tumor cells collected from cocultures were stained with anti-Annexin V-FITC antibody or PI (Invitrogen) in Annexin binding buffer) and incubated at RT for 20 min in the dark according to the manufacturer’s guidelines. After washing the cells, their staining was evaluated by ACEA Novocyte flow cytometry, which was analysed using FlowJo software. For cell viability and ATP assay, cells were measured using CellTiterGlo (Promega) at indicated time point according to the supplier’s instructions. Briefly, equal volume of CellTiterGlo reagent was added to the well-containing mono/cocultures with and without PBMC/expanded T cells in medium and incubated for 45 min at RT on a shaker. The cell suspension was then transferred to a black 96-well clear flat bottom plate and the relative luminescence units (RLU) were measured using a microplate reader. To compare tumor with and without PBMC/expanded T cells, and to compensate for PBMC expanded T cells number in this assay, we measured PBMC/expanded T cells alone as control and subtracted the relative RLU value from the tumor RLU value with PBMC for each day. For real-time cell death experiments, PBMC (effector cells) were pre-activated as mentioned above and cocultured with pre-plated tumor target cells at a ratio of 5:1 (effector:target ratio) into a 96-well opaque plate. Next cells were loaded with reagents provided in RealTime-Glo™ Annexin V Apoptosis and Necrosis Assay (Promega) according to manufacturer’s instruction. Target cell death was measured in a CLARIOstar Plate Reader using bottom reading function over 48 h at 37 °C. To normalize the sample data from positive control, data were processed as below: (positive control – sample)/ positive control. The ratio between death in sample and positive control is yielded, where lower the ratio indicates higher cell death. Tumor cells treated with 40 μM cisplatin were used as positive controls.

### Cell proliferation assay

Tumor cells (1 × 10^5^) were plated in a 96-well plate. The next day, PBMC (5 × 10^5^) was labeled with CFSE dye (carboxyfluorescein succinimidyl ester, 1:1000, eBioscience) at 37 °C for 8 min. RPMI with 10% FBS was added to terminate the reaction. Anti-CD3/CD28 were then added to stimulate T-cell proliferation. PBMC were collected on day 3 and the proliferation rate was assessed by the progressive dilution of CFSE dye. Post-acquisition analysis was performed using FlowJo software (BD Biosciences).

### Live/dead staining

Tumor cells were labeled with LIVE/DEAD™ Cell Imaging Kit (488/570) (Invitrogen) after removing supernatant from coculture system. Staining was conducted in FluoroBrite DMEM (Gibco) in RT for 15 min in dark according to manufacturer guidelines. Images were acquired via ImageXpress Micro Confocal High-Content Imaging System (Molecular Devices) and live to dead ratio was analyzed by MetaXpress Software Live/Dead Application Module.

### SYTOX green staining

24 h after tumor cells were plated, cells were labeled with CellTracker™ Deep Red dye in serum-free medium for 30 min at 37 °C. Upon completion of 6 h coculturing, tumor cells were stained with SYTOX™ Green Nucleic Acid Stain (Invitrogen) in HBSS for 15 min in dark. Images were acquired by ImageXpress Micro Confocal High-Content Imaging System (Molecular Devices) and percentage of necrotic cells was calculated using MetaXpress Software.

### Cytotoxicity assay

Target cells were labeled with PKH67, seeded in a 96-well plate at 10^5^ cells/well, and coincubated with anti-CD3/CD28 preactivated expanded T cells at the indicated effector to target cell (E:T) ratios and cultured for 72 h. Propidium iodide (PI; BD Biosciences) was then used to assess cell death and evaluated by flow cytometry. Lysis of target tumor cells was calculated as the percentage of PI+ cells among gated PKH67+ population. The percentage of specific lysis was then calculated using the following equation: % specific lysis = 100 × (% tumor cell death − % basal cell death)/100 − % basal cell death.

### Detection of mitochondrial membrane potential (ΔΨm) and measurement of intracellular reactive oxygen species (ROS)

The mitochondrial membrane potential was assessed with JC-1. JC-1 accumulates as J- aggregates inside the mitochondria in healthy cells, showing higher ΔΨm and emits red fluorescence at the wavelength of 590 nm. As membrane potential decreases, JC-1 becomes J-monomers, which shows a cytosolic green fluorescence. After coculture, target tumor cells were trypsinized, washed twice with ice-cold PBS at 400 g, each for 5 min. Cells were then suspended in 1 ml of PBS containing JC-1 (2 μM) and anti-CD45-AF700 for 15 min at 37 °C in the dark. The staining solution was removed by centrifugation and cells were washed twice by PBS. Dissipation in ΔΨm was measured by calculating the ratio the J-aggregates/monomers ratio (revealed by the red/green fluorescence; i.e., 590/530 nm) by examining sample on an FL-1 versus FL-2 dot plot with NovoCyte Quanteon flow cytometer. For positive control, cells were incubated with 10 μM CCCP for 10 min to disrupt the membrane potential.

To measure ROS, CellROX Green (Invitrogen) was added to each well-containing tumor cells at a final concentration of 5 μM according to the manufacturer’s instructions before coculturing with PBMC/expanded T cells. Paired controls with no T cells added were included for each condition. For detecting ROS in tumor cells, samples were incubated with anti-EpCAM conjugated with PE for 15 min in the dark. Samples were analyzed on a NovoCyte flow cytometer. Data were gated post-acquisition to include only single cells, and the corresponding fluorescence histograms and median fluorescence intensities (MFI) were extracted using FlowJo software (v. 10.1).

### Granzyme B activities and loading

Granzyme B (GzB) activity was measured using the GranToxiLux-PLUS kit (Oncoimmunin), according to the manufacturer’s instructions. Briefly, target cells were identified by labeling with a target fluorescent probe (TLF-4) and with a nuclear fluorescent labeling probe (NFL1), to exclude cells that had died before the start of the assay. Effector (PBMC) and target cells were coincubated at a ratio of 5:1 in the presence of a FITC-conjugated GzB substrate for 90 min. Samples were analyzed in a flow cytometer set to distinguish effector (non-fluorescent) and the target (TLF4 + NLF-) cells. Target cells were then selected and dot plots against the GzB activity (FITC) were studied to determine the percentage of target cells with GzB activity (TLF4 + FITC). For GzB loading, human recombinant GzB (Enzo Life Sciences, BML-SE238-5000) was introduced into tumor cells (1.5 × 10^5^ cells) using sublytic doses of pore-forming protein streptolysin-O (SO) (MedChemExpress). Briefly, tumor cells plated onto chamber slides. 24 h later, adherent tumor cells were washed in Hank’s Balanced Salt Solution (HBSS), followed by the addition of 100 μl of HBSS containing 0.5% FBS and 100 nM SLP. After 30 min of incubation, 10 µg recombinant pre-labeled GzB were added and incubated for 6 hours. The cells were then washed and stained with 100 nM of LysoTracker (ThermoFisher) in 0.1% Tween20 in PBS for 30 mins. Cells were then fixed by 4% PFA for 20 min and images were taken with a 40x objective on LSM900 confocal microscope (Olympus). Fluorescence intensity was measured was analyzed in Fiji (ImageJ; NIH).

### Intracellular cytokine assay

For intracellular GzB, Ki67, IFNγ, and TNFα staining, PBMCs were stimulated with anti-CD3/CD28 antibodies and co-cultured with tumor cells for 5 days. Brefeldin A (1 µg/mL; Sigma-Aldrich) and monensin (1:1000; Biolegend) were added during the last 6 h of co-culture. Cells were washed, surface stained in PBS supplemented with 2% bovine serum albumin and 0.1% sodium azide. (4 °C for 30 min) and then fixed with Cytofix/Cytoperm, followed by fluorophore-conjugated anti-mouse antibodies: Ki67-BV711, TNFα-BV650, granzyme B-PE, IFNγ-BV421. For degranulation, cells were stained with CD107a on the day of co-culture. All antibodies were purchased from BD Biosciences (Supplementary Table 3). Cells were processed on a NovoCyte Quanteon flow cytometer and the results were analyzed using FlowJo software. Non-viable cells were excluded from the analysis based on forward and side scatter profiles, and with 7-AAD dead cell staining. For the ACT product, T cells were stimulated with a custom EBV peptide pool or individual HLA-matched epitopes, then cultured for 4 h in the presence of GolgiPlug and GolgiStop (BD Bioscience). Cells were washed and stained with anti-CD8-PerCP-Cy5.5 and anti-CD4-Pacific Blue, fixed and permeabilized with Cytofix/Cytoperm, washed again and stained with anti-IFNγ-AF700. Cells were washed, then resuspended in PBS, and acquired using flow cytometer.

### CCK-8 adhesion assay

After 6 h coculturing of CD8 + T cell with tumor cells in 96-well plate, it was gently tapped twice, following by gently removing the unbound T cells. The cell density was measured by Cell Counting Kit-8 (CCK8; MedChemExpress) following the manufacturer’s instructions, with incubation time 1 h at 37 °C. Optical density (OD) was measured at 450 nm.

### 2D colony formation assay and 3D soft agar colony formation assay

Tumor cells in 6-well plates for 6 h coculture with drug added with concentration stated before were fixed, stained using 1% crystal violet, and counted by Fiji (ImageJ; NIH). For 3D soft agar colony formation assay, 96-well black-sided, clear-bottom plates (Nunc) were prepared with a 40 µl base layer of 0.8% soft agar. Soft agar 0.8% was made by melting agarose in the cell-specific growth medium, filter-sterilizing through a 0.22 µm filter. After the base layer solidified, tumor cells cocultured for 6 h were trypsinized and counted. 1200 cells were then mixed with an equal volume of 0.8% soft agar and added to each well. Plates were incubated at room temperature for 20 min to allow soft agar to solidify. A 100 µl medium overlay was added to the wells, and plates were then incubated overnight at 37 °C. After 7 days of incubation, Calcein AM dye (ThermoFisher) was added to stain live cells. Image was acquired by LSM800. 3D lollipop plot was constructed by Plotly package in R, using 3D scatter plot function.

### Western blotting

Phospho- and total-protein lysates were prepared with RIPA lysis buffer, with freshly added protease and phosphatase inhibitor cocktail (both from Thermo Fisher Scientific). For the separation of PBMC from the monolayer tumor cells, culture medium was removed and the remaining PBMC was repeatedly flushed off with PBS; the monolayer tumor cells were rinsed with cold PBMC and immediately lysed on the culture dish. Protein concentrations were measured by the bicinchoninic acid protein assay kit (Pierce). Equal amounts of protein were solubilized in Laemmli buffer, boiled for 5 min, and then resolved on an 11% SDS-PAGE, transferred to nitrocellulose membranes, and blocked with 3% milk or 5% BSA in TBST was overnight at 4 °C. Membranes were probed with primary at 4 °C overnight and then with HRP-linked secondary antibodies and visualized with ECL substrates (MedChemExpress). The dilution, clone, and catalog number of the antibodies used are listed in Supplementary Table 4.

### Antibody array

Human autophagy array (Raybiotech) was used to detect cell factors in conditioned media from cells under indicated conditions and evaluated using the human cytokine array according to the manufacturer’s guidelines.

### Fluorescence-based autophagosome detection

Fluorescence-based autophagosome was detected by the following two methods: autophagic vacuoles and autophagic flux in live cells were selectively labeled with an Autophagy Detection Kit (cat. no. ab139484; Abcam) immediately after 6-hour coculturing and measured with a fluorescent CLARIOstar plate reader according to manufacturer’s instruction. Relative readings normalized by DAPI staining were calculated. The reading from cocultured system was subtracted by the values from tumor cell cultured alone. In another method, tumor cells grown on 8-wells chamber slides (ibidi) and reached 50–70% confluences at the time of infection with Premo™ Autophagy Tandem Sensor RFP-GFP-LC3B Kit (Invitrogen). Plated tumor cells were incubated with BacMam 2.0 reagent (20 particles per cell (PPC)) for 18 h. Coculture was preformed two days post-transduction. After removing supernatant, cells were incubated with 0.05% saponin for 20 min at 4 °C. DAPI was stained before imaging on ImageXpress Micro Confocal High-Content Imaging System (Molecular Devices). Data quantification was conducted in Fiji (ImageJ; NIH) using the Spots colocalization (ComDet) plugin.

### Proteomic analysis

Phenol-red and serum-free medium was added to tumor cells for 24 h post-coculturing and condition medium was collected for protein concentration by Amicon® Ultra Centrifugal Filter. 30 μg of protein was diluted with TFEA buffer in 1:1 volumetric ratio. Protein was denatured under 60 °C for 2 hr. Samples were then reduced with dithiothreitol (DTT) in 5 mM, alkylated with 25 mM iodoacetamide (IAA) in dark and enzymatically digested with trypsin at 37 °C for 16 h, and quenched with 5% formic acid. The digested samples were desalted using C18 STAGE tips and then dried by SpeedVac (Thermo Savant). Subsequently, the desalted peptides were reconstituted in MS grade water containing 0.1% formic acid and then were analyzed using the Bruker timsTOF Pro mass spectrometer coupled with NanoElute® (Bruker) at LSCCB, InnoHK Center. PEAKS Studio (Version 11, Bioinformatics Solutions Inc.) was employed for the protein identification and relative quantification. UniProt Homo sapiens (Human) proteome database (downloaded on 12th Oct. 2023) was employed for protein identification. To identify any potential contaminant proteins within the samples, a contaminant database provided by Bioinformatics Solutions Inc. was crossed-referenced. Search parameters included fixed modification at Cys (Carbamidomethylation, m/z shift +57.02), variable modifications at Met (oxidation, m/z shift +15.99) and at the protein N-terminus (acetylation, m/z shift +42.01). Identified proteins passing the filtering parameters of FDR < 1.0%, −10 LgP > 15.00, and at least one unique peptide was selected for relative quantitation analysis.

Protein set for death pathways analysis was obtained in Gene Ontology Biological Pathway. Proteins from pathways “regulation of autophagy”, “necroptotic process” and “apoptotic process” were selected for autophagy, necrotic, and apoptotic cell death analysis respectively. Network visualization for cell lysate and condition medium was constructed by GSEA and Cytoscape. Venn diagrams were constructed by ggplot2 package in R.

### Quantitative real-time PCR

Total RNA (500 ng) was extracted with RNeasy mini kit (Qiagen) according to the manufacturer’s instructions. Total RNA (500 ng) was reversed transcribed with Primescript RT (Takara), with the cDNA serving as the template for amplification. Quantitative real-time PCR was subsequently performed using SYBR Green Master Mix (Takara) on a LightCycler480 (Roche). Relative quantification was measured using the Comparative Ct (Threshold Cycle) with the Ct values normalized to GAPDH. Primers used are shown in Supplementary Table 5.

### Immunofluorescence immunocytochemistry

Tumor cells were gelatin‐coated coverslips in 24‐well culture plates and PBMC was added onto at 5:1 ratio. PBMCs were collected following 72 hours of coculture and then washed twice with cold PBS and cytospun. Cells were then fixed in ice-cold methanol/acetone 1:1, blocked, incubated with mouse anti-human CD8 and rabbit anti-human LC3B antibody, followed by anti-mouse or anti-rabbit antibodies conjugated with Alexa555/Alexa488. In some experiments, PBMC was removed from cocultures, adherent tumor cells were rinsed with PBS, followed by lysotracker incubation as described above. Cells were then fixed with cold 4% PFA followed by antibody incubation. Tumor alone cultures were fixed with acetone/method ice-cold 1:1 Slides were then counterstained with DAPI for 5 min and mounted with VECTASHIELD. Immunofluorescent staining was observed and photographed using an LSM900 fluorescence microscope. For tumor cell culture alone, images were recorded via LSM with an Airyscan module. In some of the experiments, sorting of non-tumor cells was performed after coculture was completed in V-bottom 96-well plate. Coculture mixture were collected and stained with EpCAM marker for 20 mins. Cells were then run through BD FACSAria SORP. 8000 cells gated as EpCAM- were sorted on a microscopic slide. The slide was allowed to dried in an oven before proceeding to ICC. All antibodies used are listed in Supplementary Table 6.

### Single cell RNA sequencing (scRNA-seq) and transcriptomic expression analysis

The raw and processed scRNA-seq data are publicly available in Gene Expression Omnibus (GEO) with the accession number GSE150430 [28], GSE150825 [29] and GSE162025 [29]. Additional information is given in the Supplementary Information.

### Animal experiments

All research involving animals complied with protocols approved by the CULATR 5026-19 Committee on Animal Care. Lymphocyte subsets/EBV-specific T cells were collected to assess the phenotype and level of immune function before the infusion. The number of CD3 + CD8+, CD3 + CD4+, CD3–CD16 + CD56+, total CD3+ and CD3–CD19+ cells were detected by the BD Multitest 6-color TBNK Reagent (BD Biosciences) and analysed by Novocyte flow cytometry. The drugs for treatment are shown in Supplementary Table 7.

#### Assessment of tumor regression by human ACT treatment

This experiment is designed to assess if therapeutic efficacy of ACT can be improved in the G9 LOF NPC mice models. ACT treatment was performed as reported previously [71] and described briefly: three 8-week-old female NOD/SCID mice per group were first irradiated with 300 cGY, then inoculated once subcutaneously with 5 × 10^6^ HLA-matched C17 tumor cells (C17Vector orC17KO4) in 100ul of phosphate buffered saline + matrigel (1:1) using a 29-gauge needle under anesthesia. Tumor growth was monitored every day and around day 9, when we expected the tumors to start growing, mice were injected with 10 × 10^6^ of expanded EBV-specific T cells through the tail vein. Tumors from animals were collected for histological examination for LC3B and RIPK1 expression using chromogenic immunohistochemistry.

#### Assessment of tumor regression by autophagy inhibitors

This experiment is designed to assess if autophagy inhibition can “unleash” G9-induced resistant to cancer cell death. PBMC were expanded in vitro in the presence of recombinant IL2, as mentioned above. We assumed that T cells were rapidly-expanded (ex-REP T). Ex-REP T cells were then preactivated in vitro with anti-CD3/CD28 and cocultured with tumor target cells (NPC43 Vector or NPC43OE6) at effector: tumor ratio of 1:1 for 6 hours, where indicated, three different drugs were added subsequently at different time point: 2.5 ug/g Pepstatin, 100 ug/g NAC 60 ug/g Chloroquine (all from MedChemExpress). During the 6-hour incubation, three 8-week-old female NOD/SCID mice per group were irradiated with 300 cGy. 6 h later, the tumor cells + ex T cells were co-injected once subcutaneously in 200ul of phosphate buffered saline + matrigel (1:1) using a 29 gauge needle under anesthesia. Tumor growth was monitored 1–2 day and 2.5 ug/g Pepstatin, 100 ug/g NAC, or 60 ug/g Chloroquine was injected i.p. at days indicated. On day 16, tumors from animals were collected for histological examination for LC3B, CD8, and RIPK1 expression. Staining intensity was quantified as previously described [72].

### Patient samples

The NPC tissue samples used in this study were obtained from 92 patients admitted to Queen Mary Hospital, the University of Hong Kong. Written informed consent was obtained from all participants. Tissues were fixed and subjected to histological examination. Tissue microarray (TMA) were collected from NPC patients recruited in the Queen Mary Hospital. Details for patient number and exclusion criteria were described in the image analysis. Adjacent normal tissues were used as control. Peripheral blood mononuclear cells (PBMCs) were isolated from healthy donors (buffy coats, Hong Kong Red Cross Blood Transfusion Service) for subsequent coculture experiments. Descriptions of Multiplexed immunohistochemistry and digital image acquisition and analysis materials used, cell lines, and primary human cells/samples as well as animals are given in the Supplementary Information.

### Data availability and statistical analysis

#### Raw single cell RNA-seq data

Raw scRNA-seq data were deposited in the CNGB Nucleotide Sequence Archive (accession code: CNP0000428) and the processed gene expression data can be accessed from GSE150430, GSE150825, and GSE162025 database. The single-cell data from this study can be analyzed and visualized via a website portal (db.cngb.org/npcatlas).

#### Statistics

The data, excluding mRNA sequencing data obtained from public datasets were analyzed using GraphPad Prism 10.2.1 software. Mann–Whitney test (two-tailed), Pearson correlation, Chi-square analysis, Kaplan–Meier analysis (Log-rank), paired two-tailed Student’s t-test (for protein expression difference in Proteomics experiments), or unpaired two-tailed Student’s *t*-test (two groups) or two-way ANOVA (more than two groups) were used as appropriate. The data are presented as the means ± SEMs. A *P*-value < 0.05 was considered to indicate statistical significance. Significance was defined as follows: *P < 0.05; **P < 0.01; ***P < 0.001; ns, not significant.

## Supplementary information

Supplementary Figure 1_CMI-2024-0431R

Supplementary Figure 2_CMI-2024-0431R

Supplementary Figure 3_CMI-2024-0431R

Supplementary Figure 4_CMI-2024-0431R

Supplementary Figure 5_CMI-2024-0431R

Supplementary Figure 6_CMI-2024-0431R

Supplementary Figure 7_CMI-2024-0431R

Supplementary Figure 8_CMI-2024-0431R

Supplemetnary Informtaion(highlight)

Supplementary Figure legend(highlight)

Supplementary Table

WB raw

## References

[1] Raab-Traub N. Novel mechanisms of EBV-induced oncogenesis. Curr Opin Virol. 2012;2:453–8. 22858118 10.1016/j.coviro.2012.07.001 PMC4617531

[2] Chua MLK, Wee JTS, Hui EP, Chan ATC. Nasopharyngeal carcinoma. Lancet. 2016;387:1012–24. 26321262 10.1016/S0140-6736(15)00055-0

[3] Smith C, Lee V, Schuessler A, Beagley L, Rehan S, Tsang J, et al. Pre-emptive and therapeutic adoptive immunotherapy for nasopharyngeal carcinoma: Phenotype and effector function of T cells impact on clinical response. Oncoimmunology. 2017;6:e1273311. 28344888 10.1080/2162402X.2016.1273311 PMC5353921

[4] Chia WK, Teo M, Wang WW, Lee B, Ang SF, Tai WM, et al. Adoptive T-cell transfer and chemotherapy in the first-line treatment of metastatic and/or locally recurrent nasopharyngeal carcinoma. Mol Ther. 2014;22:132–9. 24297049 10.1038/mt.2013.242 PMC3978790

[5] Huang J, Fogg M, Wirth LJ, Daley H, Ritz J, Posner MR, et al. Epstein-Barr virus-specific adoptive immunotherapy for recurrent, metastatic nasopharyngeal carcinoma. Cancer. 2017;123:2642–50. 28222215 10.1002/cncr.30541

[6] Haslam A, Prasad V. Estimation of the percentage of US patients with cancer who are eligible for and respond to checkpoint inhibitor immunotherapy drugs. JAMA Netw Open. 2019;2:e192535. 31050774 10.1001/jamanetworkopen.2019.2535 PMC6503493

[7] Golstein P, Griffiths GM. An early history of T cell-mediated cytotoxicity. Nat Rev Immunol. 2018;18:527–35. 29662120 10.1038/s41577-018-0009-3

[8] Hoffmann P, Hofmeister R, Brischwein K, Brandl C, Crommer S, Bargou R, et al. Serial killing of tumor cells by cytotoxic T cells redirected with a CD19-/CD3-bispecific single-chain antibody construct. Int J Cancer. 2005;115:98–104. 15688411 10.1002/ijc.20908

[9] Schmidts A, Maus MV. Making CAR T cells a solid option for solid tumors. Front Immunol. 2018;9:2593. 30467505 10.3389/fimmu.2018.02593 PMC6235951

[10] Breart B, Lemaitre F, Celli S, Bousso P. Two-photon imaging of intratumoral CD8+ T cell cytotoxic activity during adoptive T cell therapy in mice. J Clin Invest. 2008;118:1390–7. 18357341 10.1172/JCI34388 PMC2268880

[11] Boissonnas A, Licata F, Poupel L, Jacquelin S, Fetler L, Krumeich S, et al. CD8+ tumor-infiltrating T cells are trapped in the tumor-dendritic cell network. Neoplasia. 2013;15:85–94. 23359264 10.1593/neo.121572 PMC3556941

[12] Wiedemann A, Depoil D, Faroudi M, Valitutti S. Cytotoxic T lymphocytes kill multiple targets simultaneously via spatiotemporal uncoupling of lytic and stimulatory synapses. Proc Natl Acad Sci USA. 2006;103:10985–90. 16832064 10.1073/pnas.0600651103 PMC1544161

[13] Cummings RD, Liu FT, Vasta GR Galectins. In: Varki A, Cummings RD, Esko JD, Stanley P, Hart GW, Aebi M, et al. editors. Essentials of Glycobiology. 3rd ed. Cold Spring Harbor (NY) 2015. p. 469–80.

[14] Thiemann S, Baum LG. Galectins and immune responses-just how do they do those things they do? Annu Rev Immunol. 2016;34:243–64. 26907217 10.1146/annurev-immunol-041015-055402

[15] Zhang CX, Huang DJ, Baloche V, Zhang L, Xu JX, Li BW, et al. Galectin-9 promotes a suppressive microenvironment in human cancer by enhancing STING degradation. Oncogenesis. 2020;9:65. 32632113 10.1038/s41389-020-00248-0 PMC7338349

[16] Klibi J, Niki T, Riedel A, Pioche-Durieu C, Souquere S, Rubinstein E, et al. Blood diffusion and Th1-suppressive effects of galectin-9-containing exosomes released by Epstein-Barr virus-infected nasopharyngeal carcinoma cells. Blood. 2009;113:1957–66. 19005181 10.1182/blood-2008-02-142596

[17] Merani S, Chen W, Elahi S. The bitter side of sweet: the role of Galectin-9 in immunopathogenesis of viral infections. Rev Med Virol. 2015;25:175–86. 25760439 10.1002/rmv.1832

[18] Sudhakar JN, Lu HH, Chiang HY, Suen CS, Hwang MJ, Wu SY, et al. Lumenal Galectin-9-Lamp2 interaction regulates lysosome and autophagy to prevent pathogenesis in the intestine and pancreas. Nat Commun. 2020;11:4286. 32855403 10.1038/s41467-020-18102-7 PMC7453023

[19] Jia J, Abudu YP, Claude-Taupin A, Gu Y, Kumar S, Choi SW, et al. Galectins control mTOR in response to endomembrane damage. Mol Cell. 2018;70:120–35.e8. 29625033 10.1016/j.molcel.2018.03.009 PMC5911935

[20] Levine B, Mizushima N, Virgin HW. Autophagy in immunity and inflammation. Nature. 2011;469:323–35. 21248839 10.1038/nature09782 PMC3131688

[21] Michaud M, Martins I, Sukkurwala AQ, Adjemian S, Ma Y, Pellegatti P, et al. Autophagy-dependent anticancer immune responses induced by chemotherapeutic agents in mice. Science. 2011;334:1573–7. 22174255 10.1126/science.1208347

[22] Noman MZ, Buart S, Van Pelt J, Richon C, Hasmim M, Leleu N, et al. The cooperative induction of hypoxia-inducible factor-1 alpha and STAT3 during hypoxia induced an impairment of tumor susceptibility to CTL-mediated cell lysis. J Immunol. 2009;182:3510–21. 19265129 10.4049/jimmunol.0800854

[23] Akalay I, Janji B, Hasmim M, Noman MZ, Thiery JP, Mami-Chouaib F, et al. EMT impairs breast carcinoma cell susceptibility to CTL-mediated lysis through autophagy induction. Autophagy. 2013;9:1104–6. 23635487 10.4161/auto.24728 PMC3722321

[24] Akalay I, Janji B, Hasmim M, Noman MZ, Andre F, De Cremoux P, et al. Epithelial-to-mesenchymal transition and autophagy induction in breast carcinoma promote escape from T-cell-mediated lysis. Cancer Res. 2013;73:2418–27. 23436798 10.1158/0008-5472.CAN-12-2432

[25] Noman MZ, Janji B, Kaminska B, Van Moer K, Pierson S, Przanowski P, et al. Blocking hypoxia-induced autophagy in tumors restores cytotoxic T-cell activity and promotes regression. Cancer Res. 2011;71:5976–86. 21810913 10.1158/0008-5472.CAN-11-1094

[26] Pioche-Durieu C, Keryer C, Souquere S, Bosq J, Faigle W, Loew D, et al. In nasopharyngeal carcinoma cells, Epstein-Barr virus LMP1 interacts with galectin 9 in membrane raft elements resistant to simvastatin. J Virol. 2005;79:13326–37. 16227255 10.1128/JVI.79.21.13326-13337.2005 PMC1262583

[27] Huang YK, Wang M, Sun Y, Di Costanzo N, Mitchell C, Achuthan A, et al. Macrophage spatial heterogeneity in gastric cancer defined by multiplex immunohistochemistry. Nat Commun. 2019;10:3928. 31477692 10.1038/s41467-019-11788-4 PMC6718690

[28] Chen HY, Wu YF, Chou FC, Wu YH, Yeh LT, Lin KI, et al. Intracellular Galectin-9 enhances proximal TCR signaling and potentiates autoimmune diseases. J Immunol. 2020;204:1158–72. 31969388 10.4049/jimmunol.1901114

[29] Gong L, Kwong DL, Dai W, Wu P, Li S, Yan Q, et al. Comprehensive single-cell sequencing reveals the stromal dynamics and tumor-specific characteristics in the microenvironment of nasopharyngeal carcinoma. Nat Commun. 2021;12:1540. 33750785 10.1038/s41467-021-21795-z PMC7943808

[30] Liu Y, He S, Wang XL, Peng W, Chen QY, Chi DM, et al. Tumour heterogeneity and intercellular networks of nasopharyngeal carcinoma at single cell resolution. Nat Commun. 2021;12:741. 33531485 10.1038/s41467-021-21043-4 PMC7854640

[31] Li Y, Feng J, Geng S, Geng S, Wei H, Chen G, et al. The N- and C-terminal carbohydrate recognition domains of galectin-9 contribute differently to its multiple functions in innate immunity and adaptive immunity. Mol Immunol. 2011;48:670–7. 21146220 10.1016/j.molimm.2010.11.011

[32] Hirashima M, Kashio Y, Nishi N, Yamauchi A, Imaizumi TA, Kageshita T, et al. Galectin-9 in physiological and pathological conditions. Glycoconj J. 2002;19:593–600. 14758084 10.1023/B:GLYC.0000014090.63206.2f

[33] Gooden MJ, Wiersma VR, Samplonius DF, Gerssen J, van Ginkel RJ, Nijman HW, et al. Galectin-9 activates and expands human T-helper 1 cells. PLoS One. 2013;8:e65616. 23741502 10.1371/journal.pone.0065616 PMC3669208

[34] Cruz-Guilloty F, Pipkin ME, Djuretic IM, Levanon D, Lotem J, Lichtenheld MG, et al. Runx3 and T-box proteins cooperate to establish the transcriptional program of effector CTLs. J Exp Med. 2009;206:51–9. 19139168 10.1084/jem.20081242 PMC2626671

[35] Pearce EL, Mullen AC, Martins GA, Krawczyk CM, Hutchins AS, Zediak VP, et al. Control of effector CD8+ T cell function by the transcription factor Eomesodermin. Science. 2003;302:1041–3. 14605368 10.1126/science.1090148

[36] Sallusto F, Kremmer E, Palermo B, Hoy A, Ponath P, Qin S, et al. Switch in chemokine receptor expression upon TCR stimulation reveals novel homing potential for recently activated T cells. Eur J Immunol. 1999;29:2037–45. 10382767 10.1002/(SICI)1521-4141(199906)29:06<2037::AID-IMMU2037>3.0.CO;2-V

[37] Betts MR, Brenchley JM, Price DA, De Rosa SC, Douek DC, Roederer M, et al. Sensitive and viable identification of antigen-specific CD8+ T cells by a flow cytometric assay for degranulation. J Immunol Methods. 2003;281:65–78. 14580882 10.1016/s0022-1759(03)00265-5

[38] Cambi A, Beeren I, Joosten B, Fransen JA, Figdor CG. The C-type lectin DC-SIGN internalizes soluble antigens and HIV-1 virions via a clathrin-dependent mechanism. Eur J Immunol. 2009;39:1923–8. 19585517 10.1002/eji.200939351

[39] Bird CH, Sun J, Ung K, Karambalis D, Whisstock JC, Trapani JA, et al. Cationic sites on granzyme B contribute to cytotoxicity by promoting its uptake into target cells. Mol Cell Biol. 2005;25:7854–67. 16107729 10.1128/MCB.25.17.7854-7867.2005 PMC1190293

[40] Khazen R, Muller S, Lafouresse F, Valitutti S, Cussat-Blanc S. Sequential adjustment of cytotoxic T lymphocyte densities improves efficacy in controlling tumor growth. Sci Rep. 2019;9:12308. 31444380 10.1038/s41598-019-48711-2 PMC6707257

[41] Duchen MR. Contributions of mitochondria to animal physiology: from homeostatic sensor to calcium signalling and cell death. J Physiol. 1999;516:1–17. 10066918 10.1111/j.1469-7793.1999.001aa.x PMC2269224

[42] Leist M, Single B, Castoldi AF, Kuhnle S, Nicotera P. Intracellular adenosine triphosphate (ATP) concentration: a switch in the decision between apoptosis and necrosis. J Exp Med. 1997;185:1481–6. 9126928 10.1084/jem.185.8.1481 PMC2196283

[43] Galluzzi L, Vitale I, Aaronson SA, Abrams JM, Adam D, Agostinis P, et al. Molecular mechanisms of cell death: recommendations of the Nomenclature Committee on Cell Death 2018. Cell Death Differ. 2018;25:486–541. 29362479 10.1038/s41418-017-0012-4 PMC5864239

[44] Denny P, Feuermann M, Hill DP, Lovering RC, Plun-Favreau H, Roncaglia P. Exploring autophagy with gene ontology. Autophagy. 2018;14:419–36. 29455577 10.1080/15548627.2017.1415189 PMC5915032

[45] Boland B, Kumar A, Lee S, Platt FM, Wegiel J, Yu WH, et al. Autophagy induction and autophagosome clearance in neurons: relationship to autophagic pathology in Alzheimer’s disease. J Neurosci. 2008;28:6926–37. 18596167 10.1523/JNEUROSCI.0800-08.2008 PMC2676733

[46] Yoshii SR, Mizushima N. Monitoring and measuring autophagy. Int J Mol Sci. 2017;18:1865. 10.3390/ijms18091865 PMC5618514 28846632

[47] Dai SH, Chen T, Li X, Yue KY, Luo P, Yang LK, et al. Sirt3 confers protection against neuronal ischemia by inducing autophagy: Involvement of the AMPK-mTOR pathway. Free Radic Biol Med. 2017;108:345–53. 28396174 10.1016/j.freeradbiomed.2017.04.005

[48] Chang CH, Lee CY, Lu CC, Tsai FJ, Hsu YM, Tsao JW, et al. Resveratrol-induced autophagy and apoptosis in cisplatin-resistant human oral cancer CAR cells: A key role of AMPK and Akt/mTOR signaling. Int J Oncol. 2017;50:873–82. 28197628 10.3892/ijo.2017.3866

[49] Guenal I, Sidoti-de Fraisse C, Gaumer S, Mignotte B. Bcl-2 and Hsp27 act at different levels to suppress programmed cell death. Oncogene. 1997;15:347–60. 9233769 10.1038/sj.onc.1201182

[50] Wang X, Wu WKK, Gao J, Li Z, Dong B, Lin X, et al. Autophagy inhibition enhances PD-L1 expression in gastric cancer. J Exp Clin Cancer Res. 2019;38:140. 30925913 10.1186/s13046-019-1148-5 PMC6440013

[51] Yasinska IM, Sakhnevych SS, Pavlova L, Teo Hansen Selno A, Teuscher Abeleira AM, Benlaouer O, et al. The Tim-3-Galectin-9 Pathway and Its Regulatory Mechanisms in Human Breast Cancer. Front Immunol. 2019;10:1594. 31354733 10.3389/fimmu.2019.01594 PMC6637653

[52] Schlichtner S, Yasinska IM, Lall GS, Berger SM, Ruggiero S, Cholewa D, et al. T lymphocytes induce human cancer cells derived from solid malignant tumors to secrete galectin-9 which facilitates immunosuppression in cooperation with other immune checkpoint proteins. J Immunother Cancer. 2023;11:e005714. 10.1136/jitc-2022-005714 PMC9815087 36599470

[53] Borowicz S, Van Scoyk M, Avasarala S, Karuppusamy Rathinam MK, Tauler J, Bikkavilli RK, et al. The soft agar colony formation assay. J Vis Exp. 2014:e51998. 10.3791/51998 PMC4353381 25408172

[54] De Sousa Linhares A, Battin C, Jutz S, Leitner J, Hafner C, Tobias J, et al. Therapeutic PD-L1 antibodies are more effective than PD-1 antibodies in blocking PD-1/PD-L1 signaling. Sci Rep. 2019;9:11472. 31391510 10.1038/s41598-019-47910-1 PMC6685986

[55] Su EW, Bi S, Kane LP. Galectin-9 regulates T helper cell function independently of Tim-3. Glycobiology. 2011;21:1258–65. 21187321 10.1093/glycob/cwq214 PMC3167474

[56] Matsuura A, Tsukada J, Mizobe T, Higashi T, Mouri F, Tanikawa R, et al. Intracellular galectin-9 activates inflammatory cytokines in monocytes. Genes Cells. 2009;14:511–21. 19335620 10.1111/j.1365-2443.2009.01287.x

[57] Querol Cano L, Tagit O, Dolen Y, van Duffelen A, Dieltjes S, Buschow SI, et al. Intracellular galectin-9 controls dendritic cell function by maintaining plasma membrane rigidity. iScience. 2019;22:240–55. 31786520 10.1016/j.isci.2019.11.019 PMC6906692

[58] Stillman BN, Hsu DK, Pang M, Brewer CF, Johnson P, Liu FT, et al. Galectin-3 and galectin-1 bind distinct cell surface glycoprotein receptors to induce T cell death. J Immunol. 2006;176:778–89. 16393961 10.4049/jimmunol.176.2.778

[59] Okoye I, Xu L, Motamedi M, Parashar P, Walker JW, Elahi S. Galectin-9 expression defines exhausted T cells and impaired cytotoxic NK cells in patients with virus-associated solid tumors. J Immunother Cancer. 2020;8:e001849. 10.1136/jitc-2020-001849 PMC7735134 33310773

[60] Sun X, Wang WJ, Lang J, Yang R, Shen WJ, Sun L, et al. Inhibition of galectin-9 sensitizes tumors to anthracycline treatment via inducing antitumor immunity. Int J Biol Sci. 2023;19:4644–56. 37781042 10.7150/ijbs.84108 PMC10535704

[61] Ma B, Khan KS, Xu T, Xeque Amada J, Guo Z, Huang Y, et al. Targeted protein O-GlcNAcylation using bifunctional small molecules. J Am Chem Soc. 2024;146:9779–89. 10.1021/jacs.3c14380 PMC11009946 38561350

[62] Farkas T, Daugaard M, Jaattela M. Identification of small molecule inhibitors of phosphatidylinositol 3-kinase and autophagy. J Biol Chem. 2011;286:38904–12. 21930714 10.1074/jbc.M111.269134 PMC3234715

[63] Wu YT, Tan HL, Huang Q, Ong CN, Shen HM. Activation of the PI3K-Akt-mTOR signaling pathway promotes necrotic cell death via suppression of autophagy. Autophagy. 2009;5:824–34. 19556857 10.4161/auto.9099

[64] Khan MJ, Rizwan Alam M, Waldeck-Weiermair M, Karsten F, Groschner L, Riederer M, et al. Inhibition of autophagy rescues palmitic acid-induced necroptosis of endothelial cells. J Biol Chem. 2012;287:21110–20. 22556413 10.1074/jbc.M111.319129 PMC3375534

[65] Wang JY, Xia Q, Chu KT, Pan J, Sun LN, Zeng B, et al. Severe global cerebral ischemia-induced programmed necrosis of hippocampal CA1 neurons in rat is prevented by 3-methyladenine: a widely used inhibitor of autophagy. J Neuropathol Exp Neurol. 2011;70:314–22. 21412169 10.1097/NEN.0b013e31821352bd

[66] Schenk B, Fulda S. Reactive oxygen species regulate Smac mimetic/TNFalpha-induced necroptotic signaling and cell death. Oncogene. 2015;34:5796–806. 25867066 10.1038/onc.2015.35

[67] Degterev A, Huang Z, Boyce M, Li Y, Jagtap P, Mizushima N, et al. Chemical inhibitor of nonapoptotic cell death with therapeutic potential for ischemic brain injury. Nat Chem Biol. 2005;1:112–9. 16408008 10.1038/nchembio711

[68] Kam NW, Laczka O, Li X, Wilkinson J, Hung D, Lai SPH, et al. ENOX2 inhibition enhances infiltration of effector memory T-cell and mediates response to chemotherapy in immune-quiescent nasopharyngeal carcinoma. J Adv Res. 2024;56:69–86. 37061217 10.1016/j.jare.2023.04.001 PMC10834794

[69] Curley J, Conaway MR, Chinn Z, Duska L, Stoler M, Mills AM. Looking past PD-L1: expression of immune checkpoint TIM-3 and its ligand galectin-9 in cervical and vulvar squamous neoplasia. Mod Pathol. 2020;33:1182–92. 32139873 10.1038/s41379-019-0433-3

[70] Ana Choi SS, Ko JM, Yu VZ, Ning L, Lung ML. Differentiation-related zinc finger protein 750 suppresses cell growth in esophageal squamous cell carcinoma. Oncol Lett. 2021;22:513. 33986873 10.3892/ol.2021.12774 PMC8114471

[71] Sinha D, Srihari S, Beckett K, Le Texier L, Solomon M, Panikkar A, et al. ‘Off-the-shelf’ allogeneic antigen-specific adoptive T-cell therapy for the treatment of multiple EBV-associated malignancies. J Immunother Cancer. 2021;9:e001608. 10.1136/jitc-2020-001608 PMC7887372 33589524

[72] Crowe AR, Yue W. Semi-quantitative determination of protein expression using immunohistochemistry staining and analysis: an integrated protocol. Bio Protoc. 2019;9:e3465. 10.21769/BioProtoc.3465 PMC6924920 31867411

