# Supplementary figures and images for: Cell-associated galectin 9 interacts with cytotoxic T cells confers resistance to tumor killing in nasopharyngeal carcinoma through autophagy activation
Source: Cell Mol Immunol. 2025 Feb 5;22(3):260–81. doi: 10.1038/s41423-024-01253-8 (PMC11868493; doi:10.1038/s41423-024-01253-8)

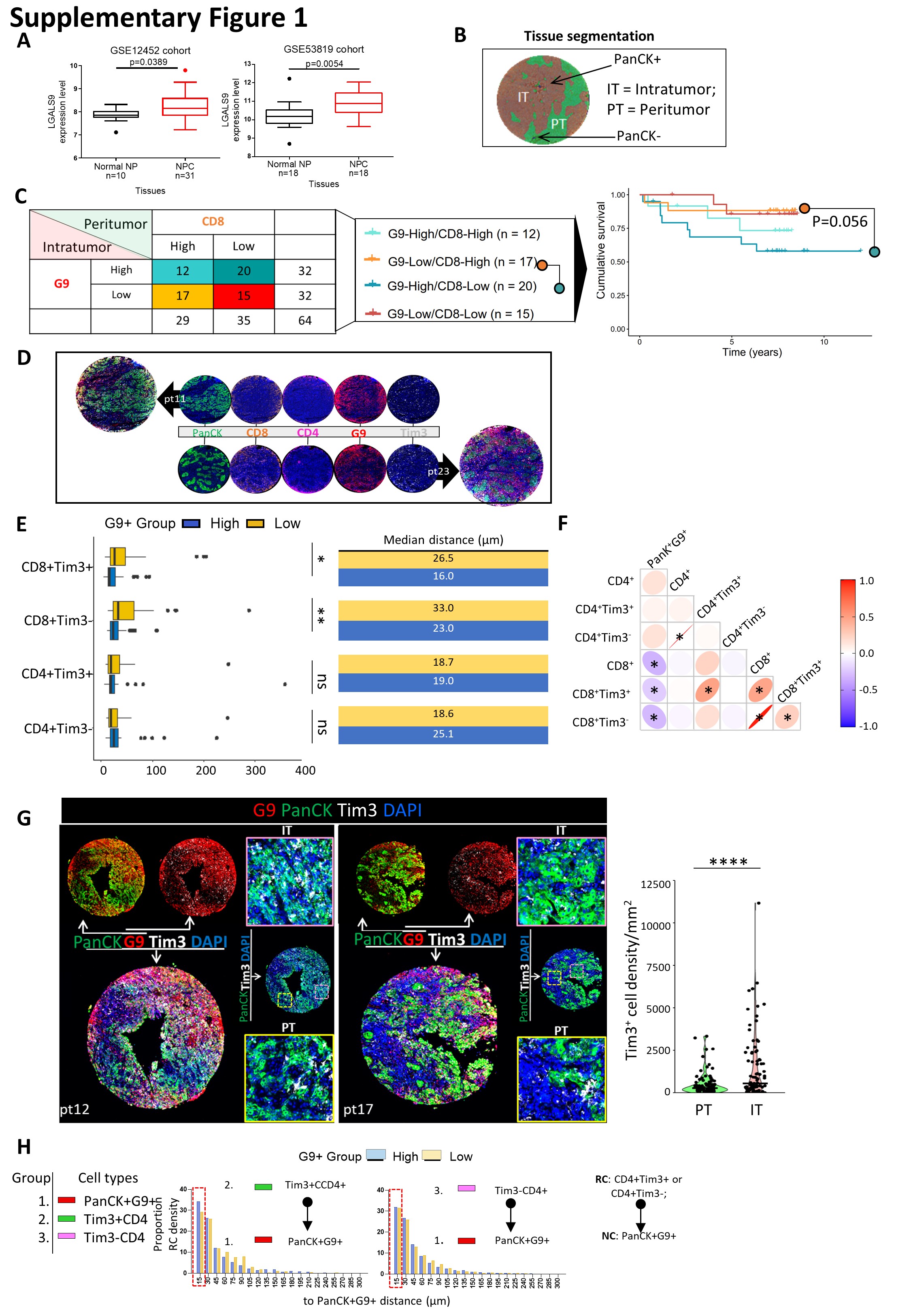

Supplement: Supplementary file 1 — Supplementary Figure 1_CMI-2024-0431R [file 41423_2024_1253_MOESM1_ESM.jpg]

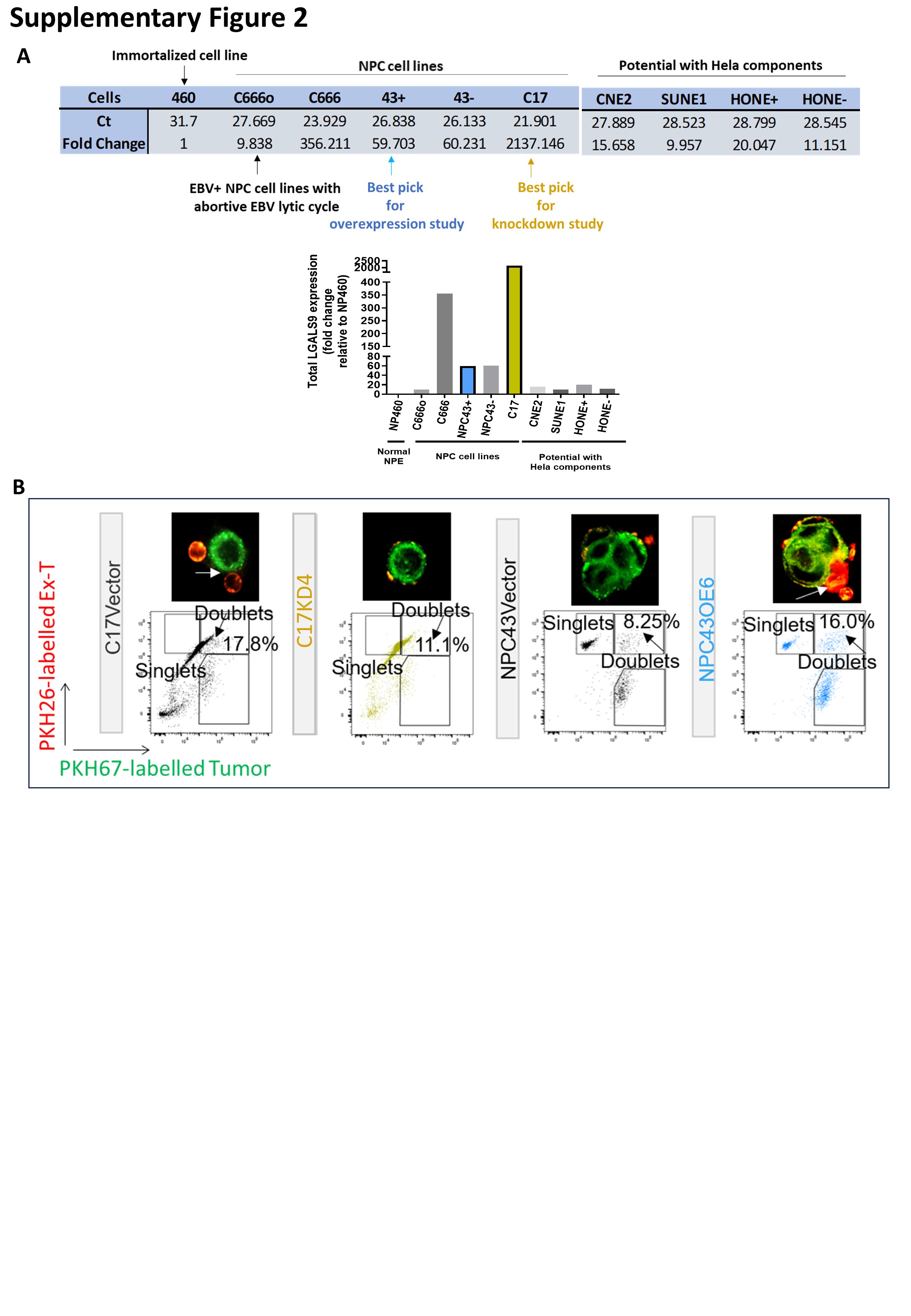

Supplement: Supplementary file 2 — Supplementary Figure 2_CMI-2024-0431R [file 41423_2024_1253_MOESM2_ESM.jpg]

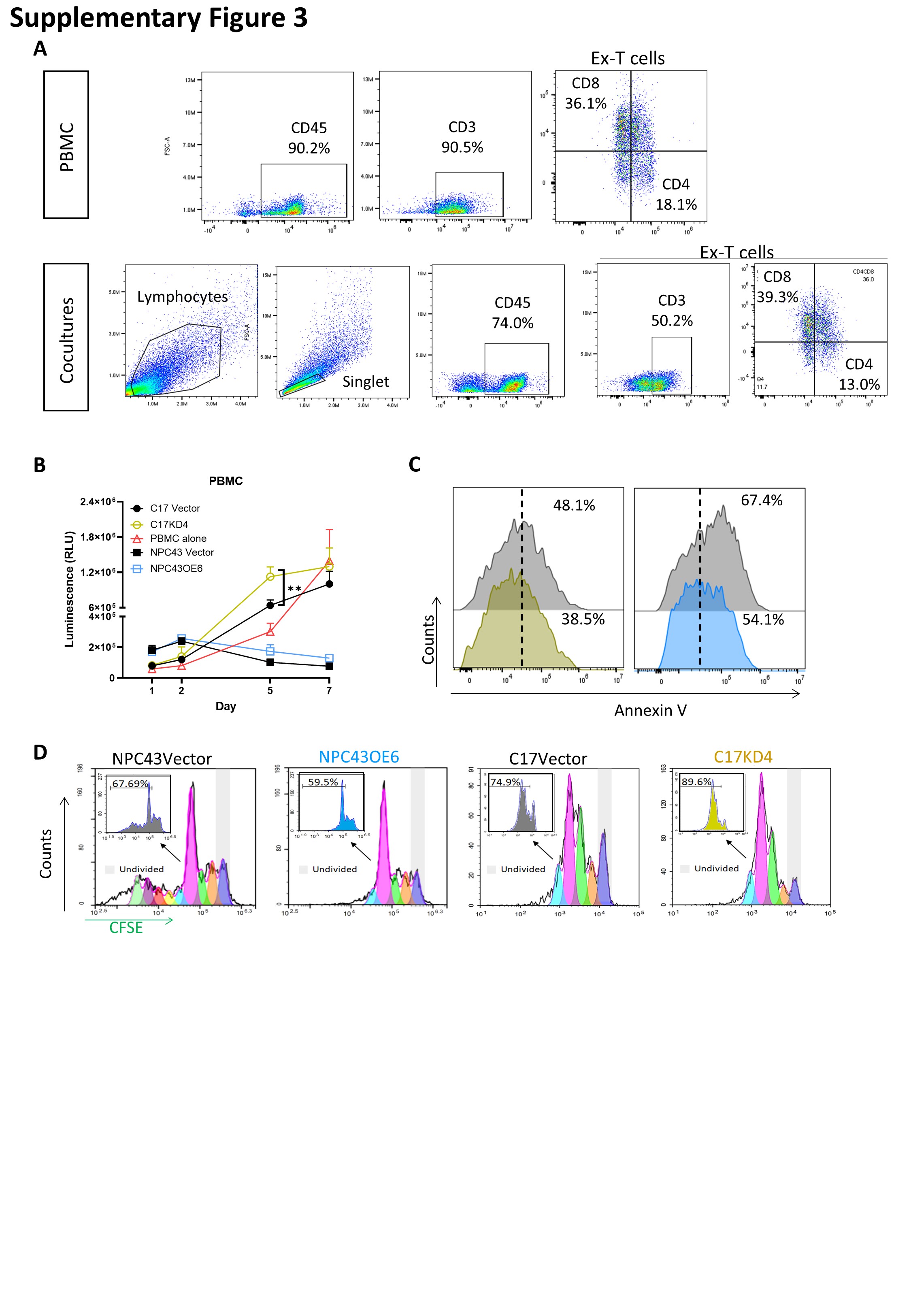

Supplement: Supplementary file 3 — Supplementary Figure 3_CMI-2024-0431R [file 41423_2024_1253_MOESM3_ESM.jpg]

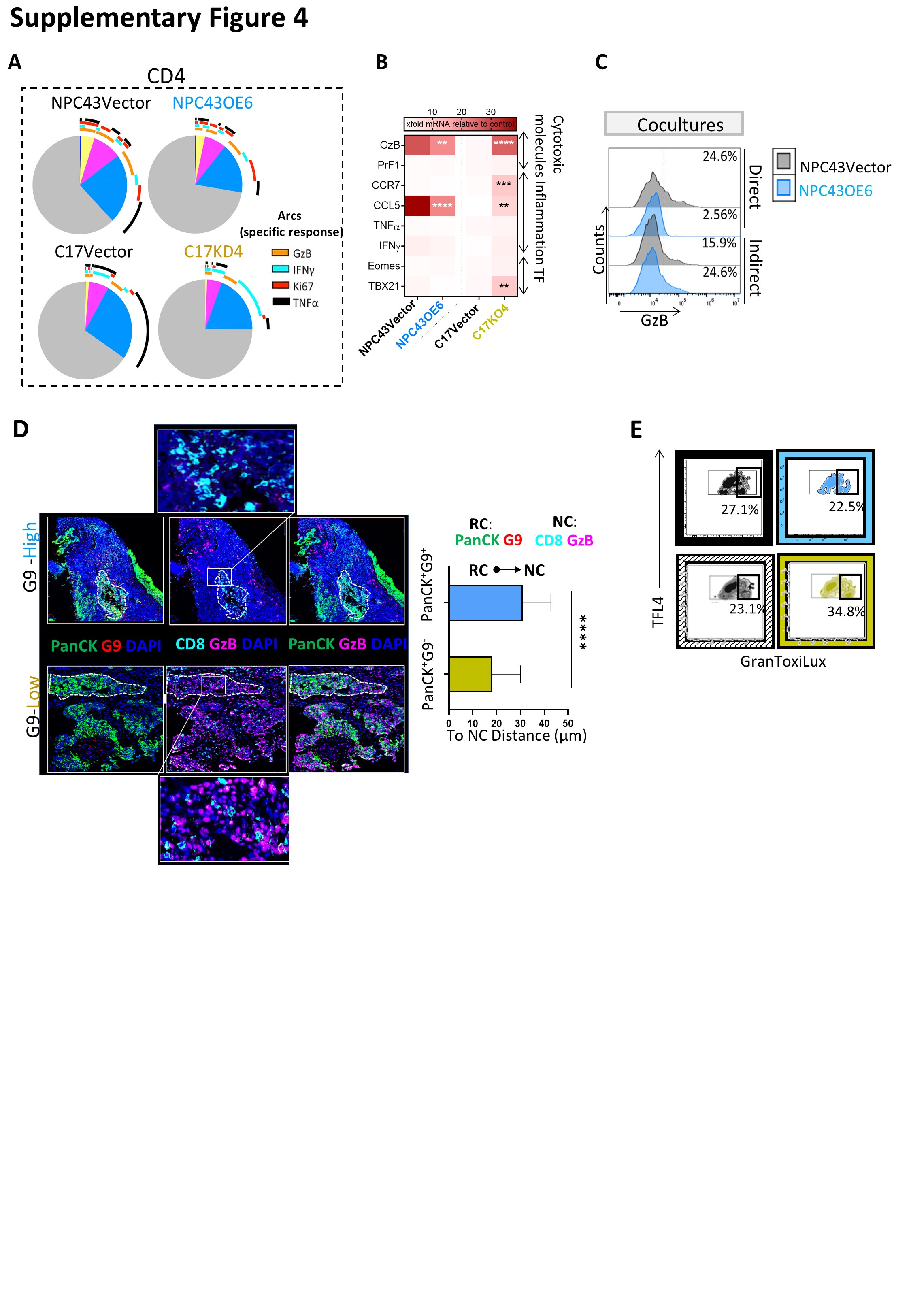

Supplement: Supplementary file 4 — Supplementary Figure 4_CMI-2024-0431R [file 41423_2024_1253_MOESM4_ESM.jpg]

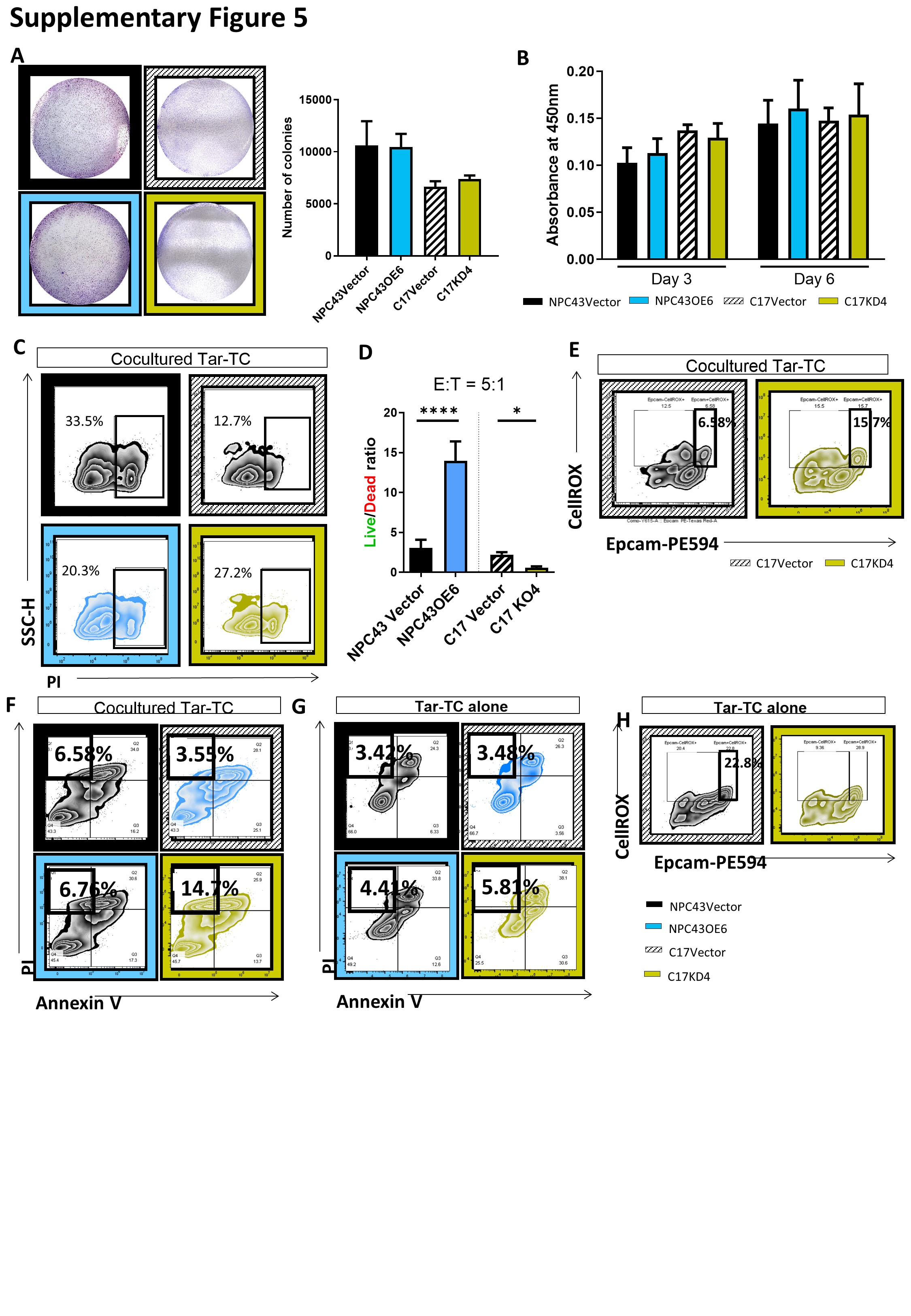

Supplement: Supplementary file 5 — Supplementary Figure 5_CMI-2024-0431R [file 41423_2024_1253_MOESM5_ESM.jpg]

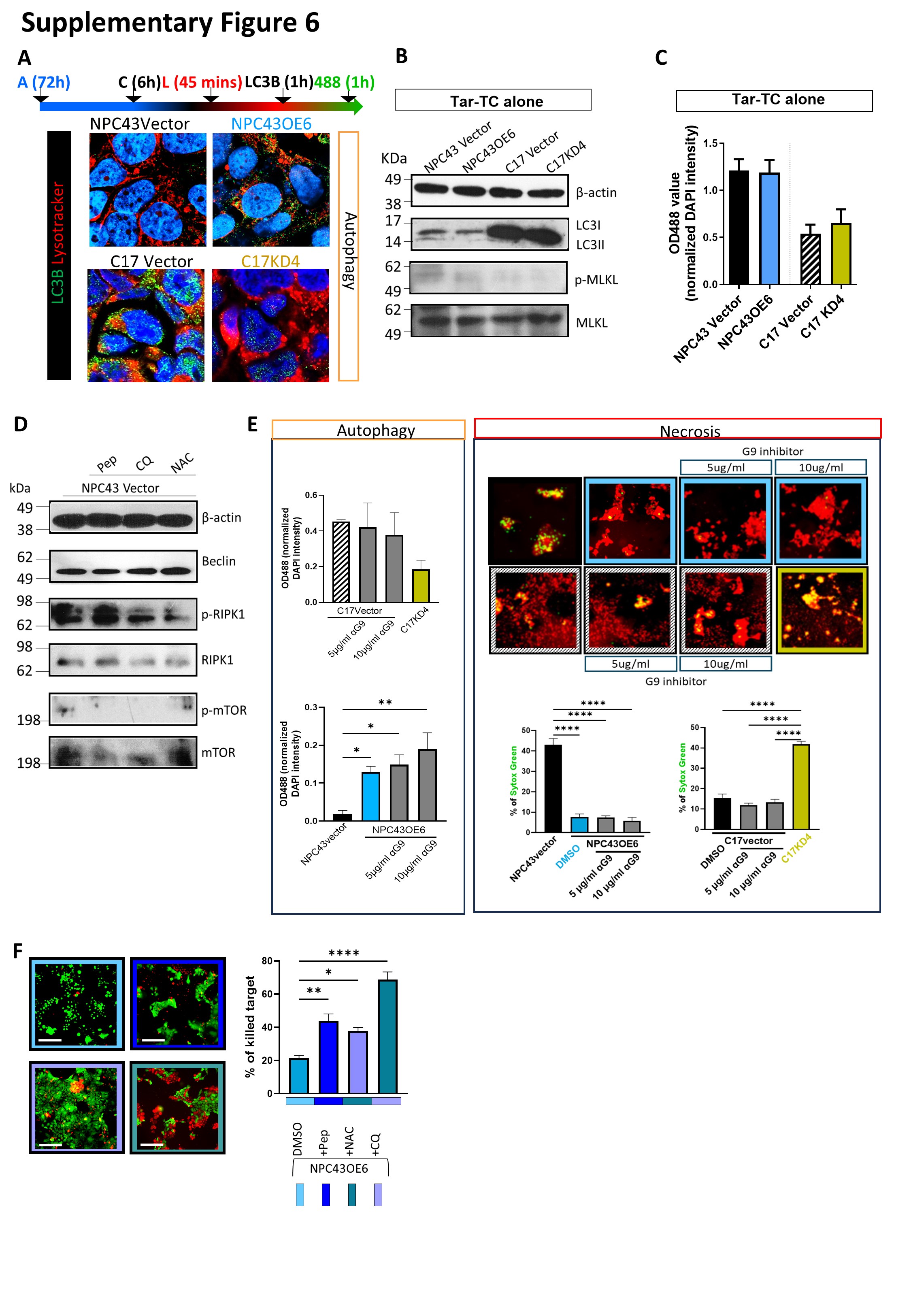

Supplement: Supplementary file 6 — Supplementary Figure 6_CMI-2024-0431R [file 41423_2024_1253_MOESM6_ESM.jpg]

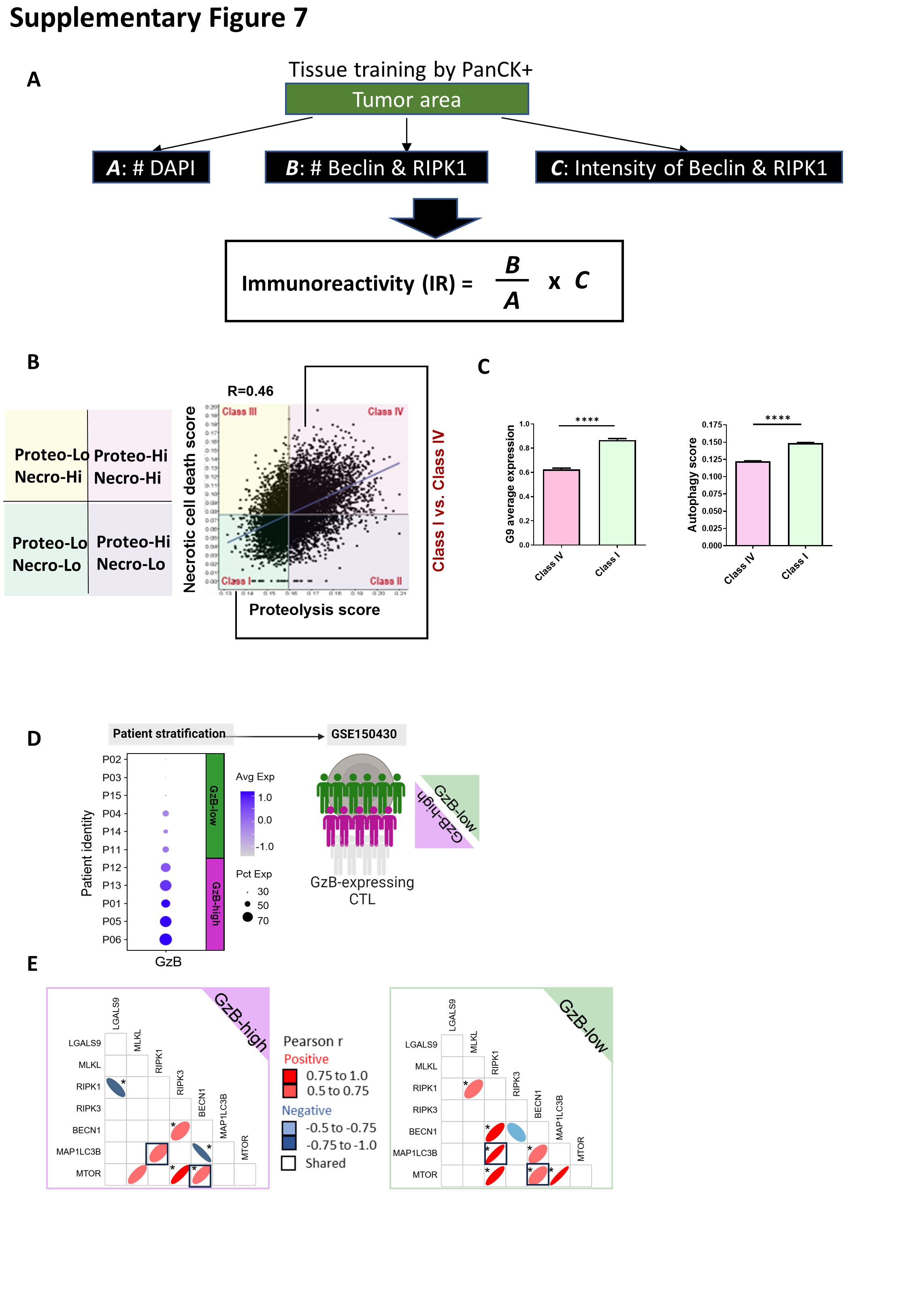

Supplement: Supplementary file 7 — Supplementary Figure 7_CMI-2024-0431R [file 41423_2024_1253_MOESM7_ESM.jpg]

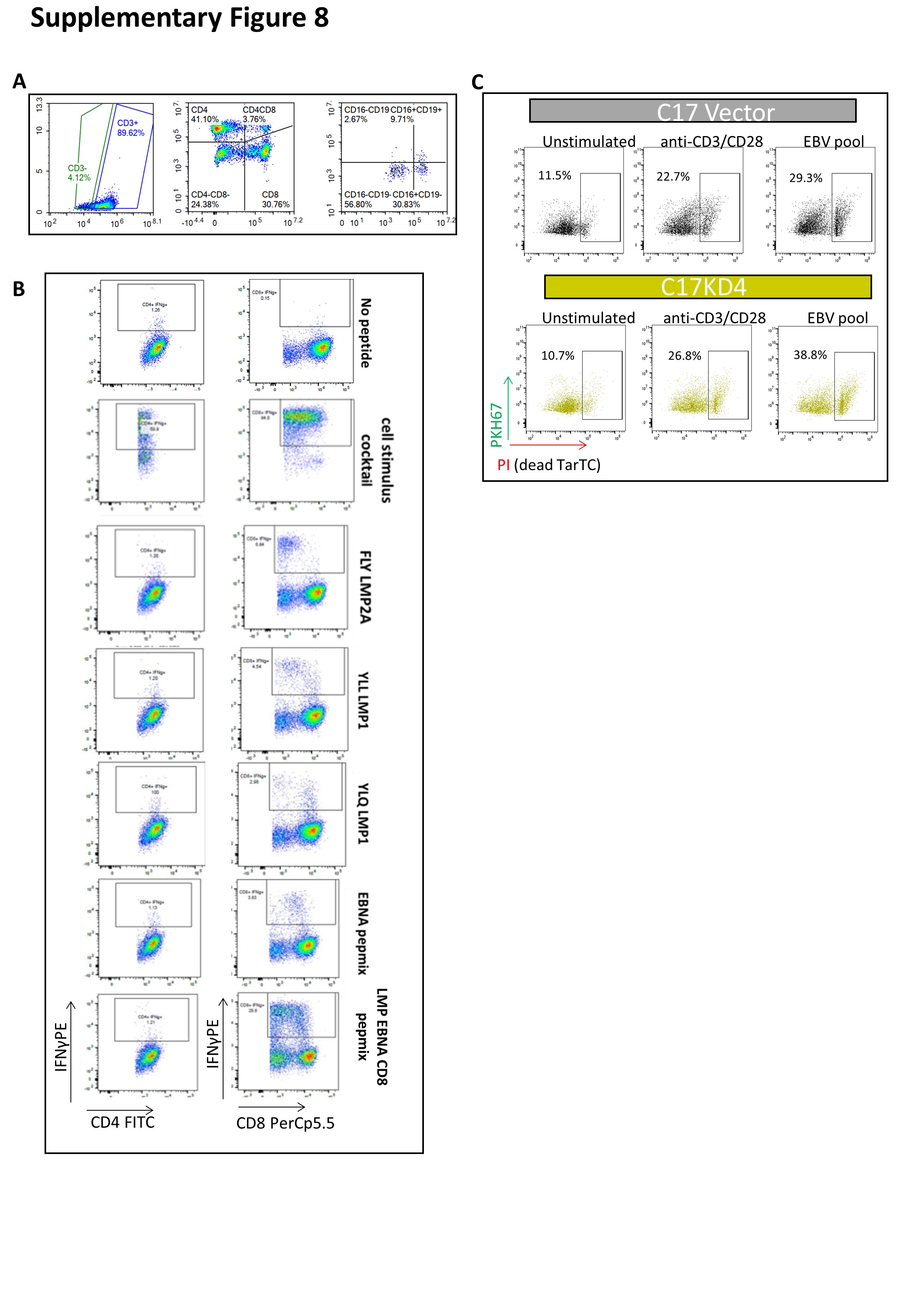

Supplement: Supplementary file 8 — Supplementary Figure 8_CMI-2024-0431R [file 41423_2024_1253_MOESM8_ESM.jpg]

Fig 2C

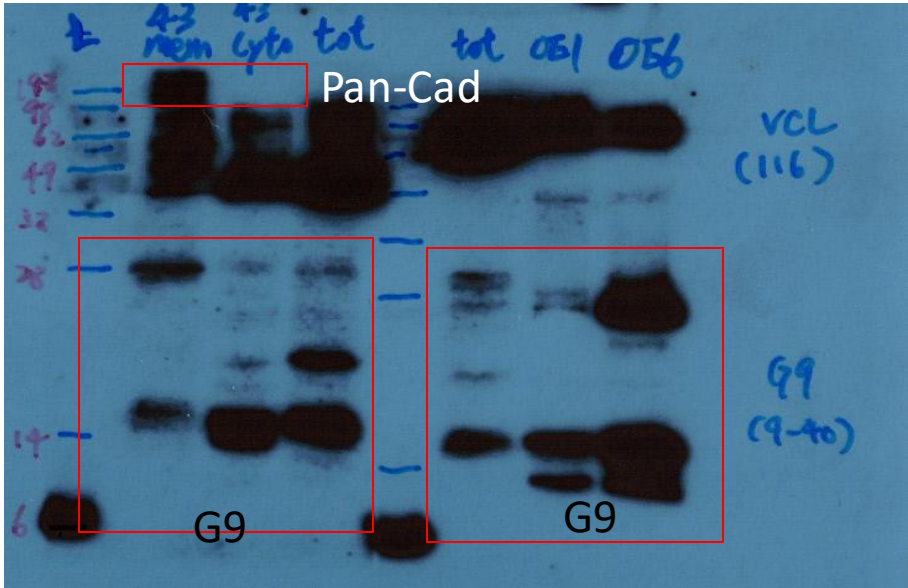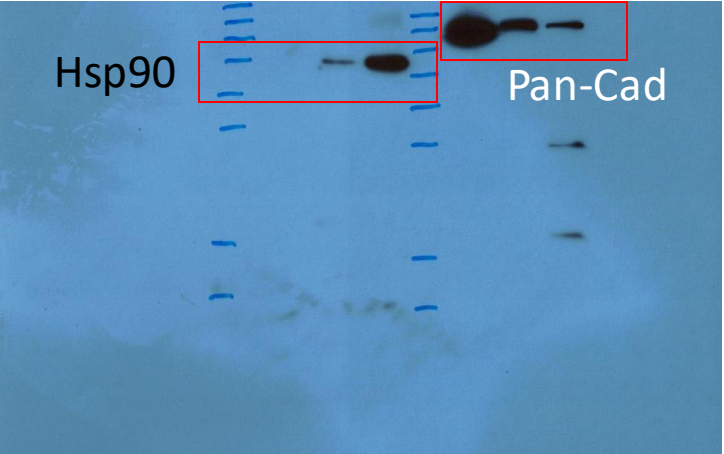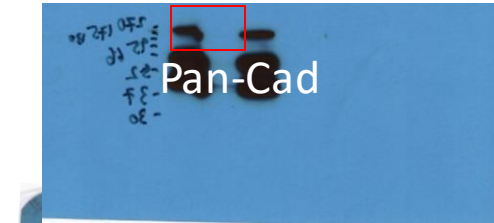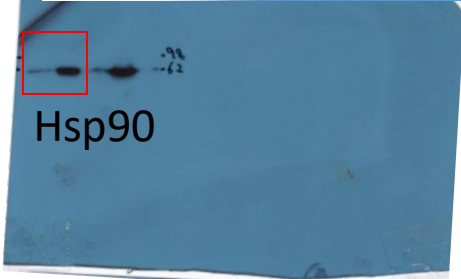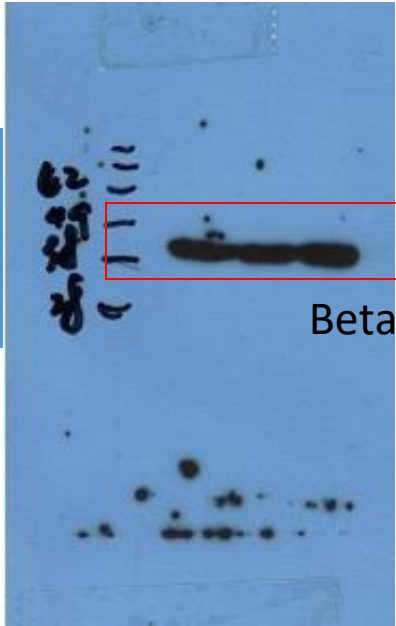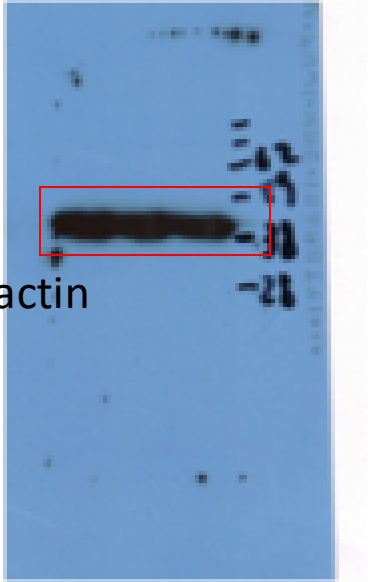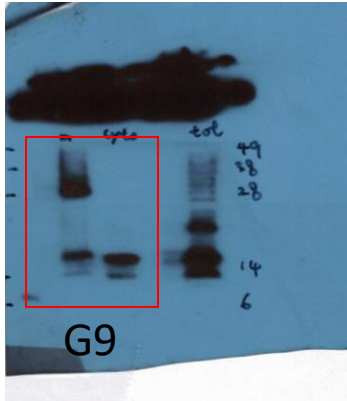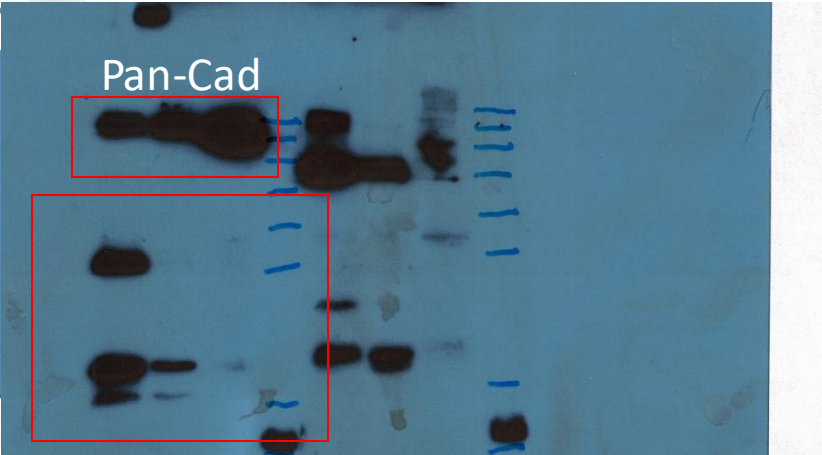

G9

Fig 4H

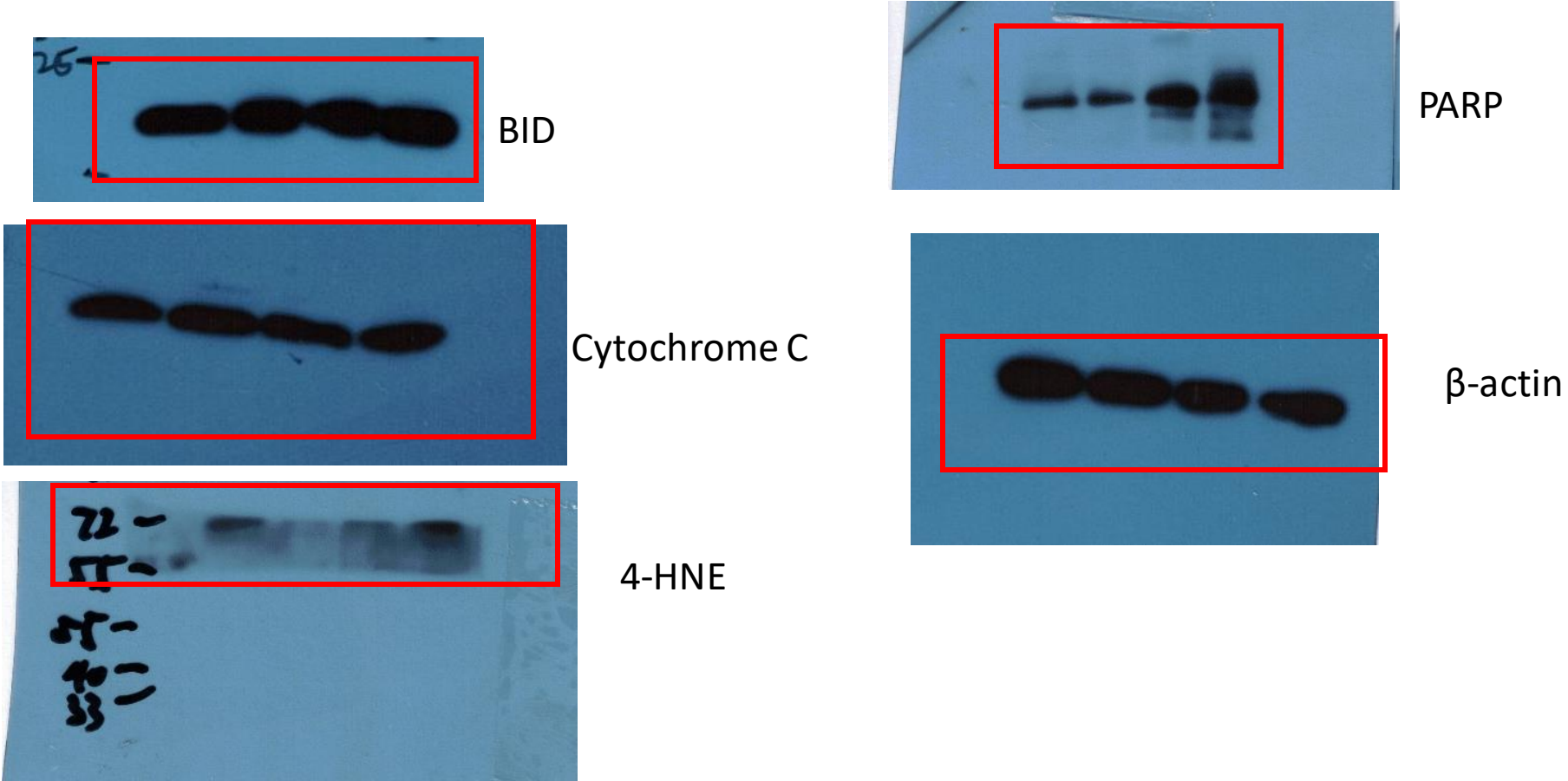

Supp fig 6b

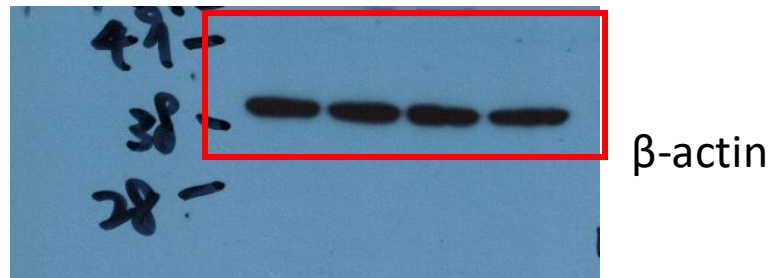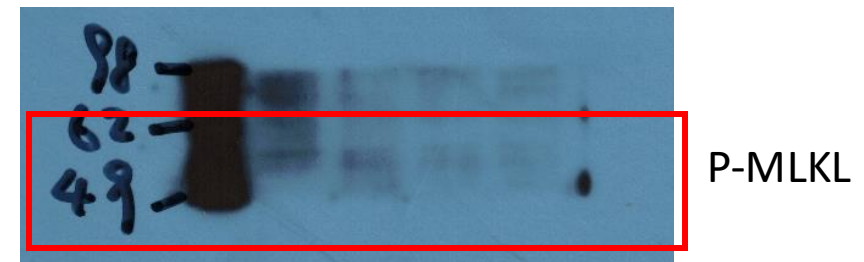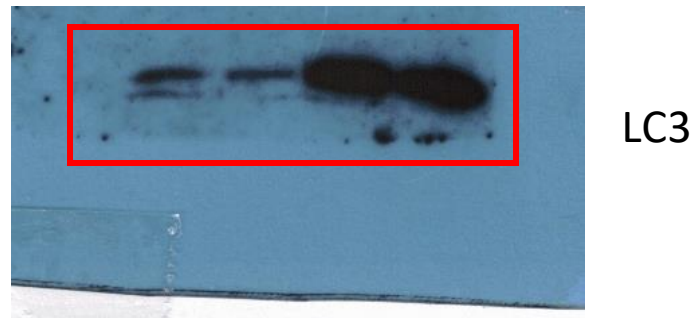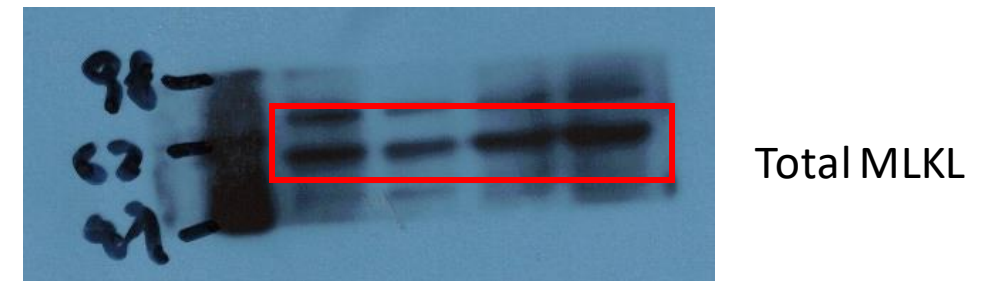

Supp fig 6d

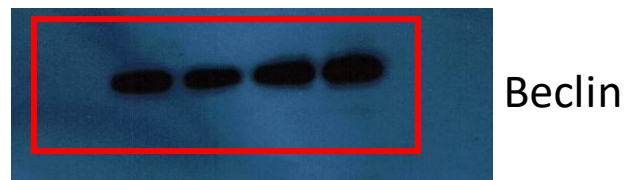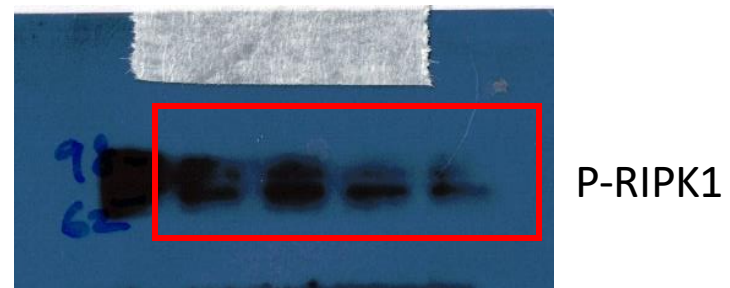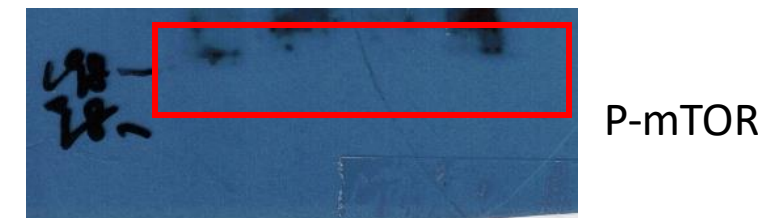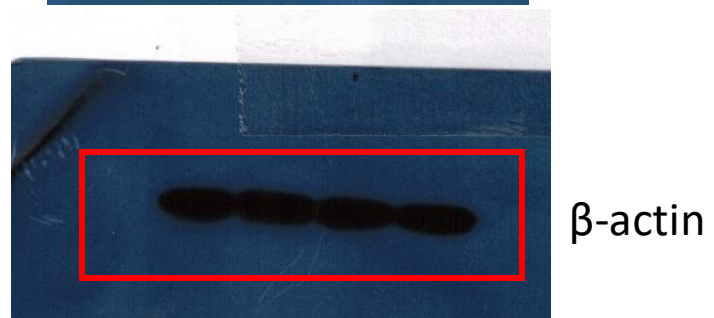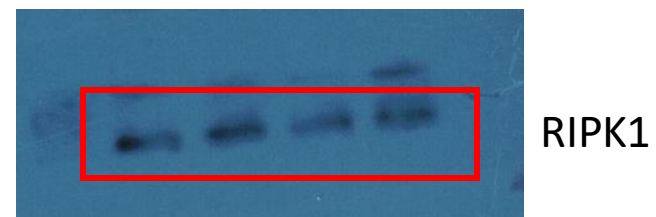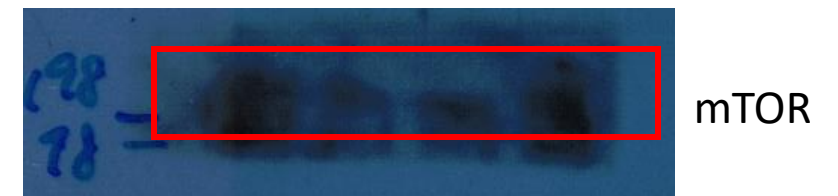

Fig 6b

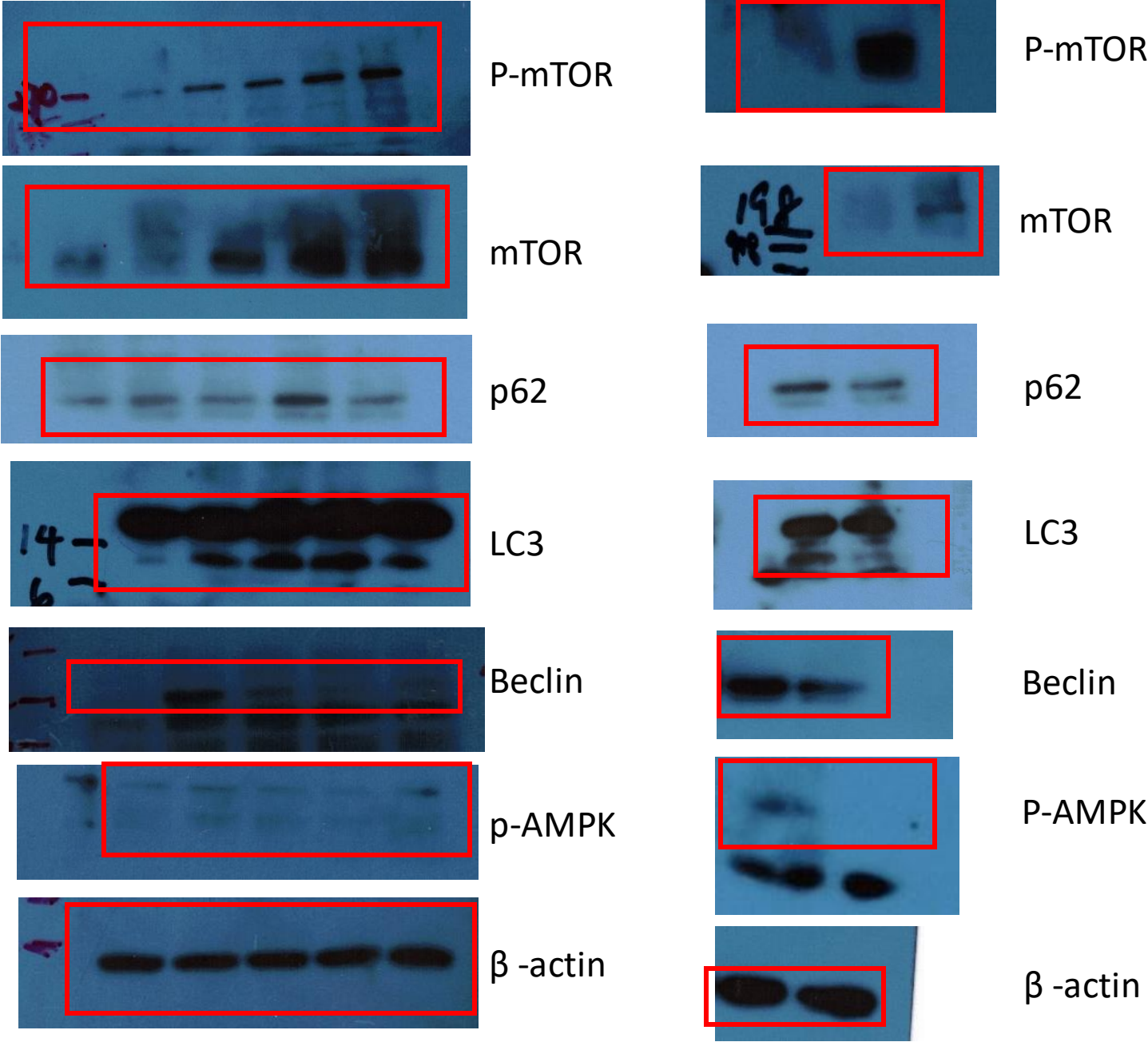

Fig 6g

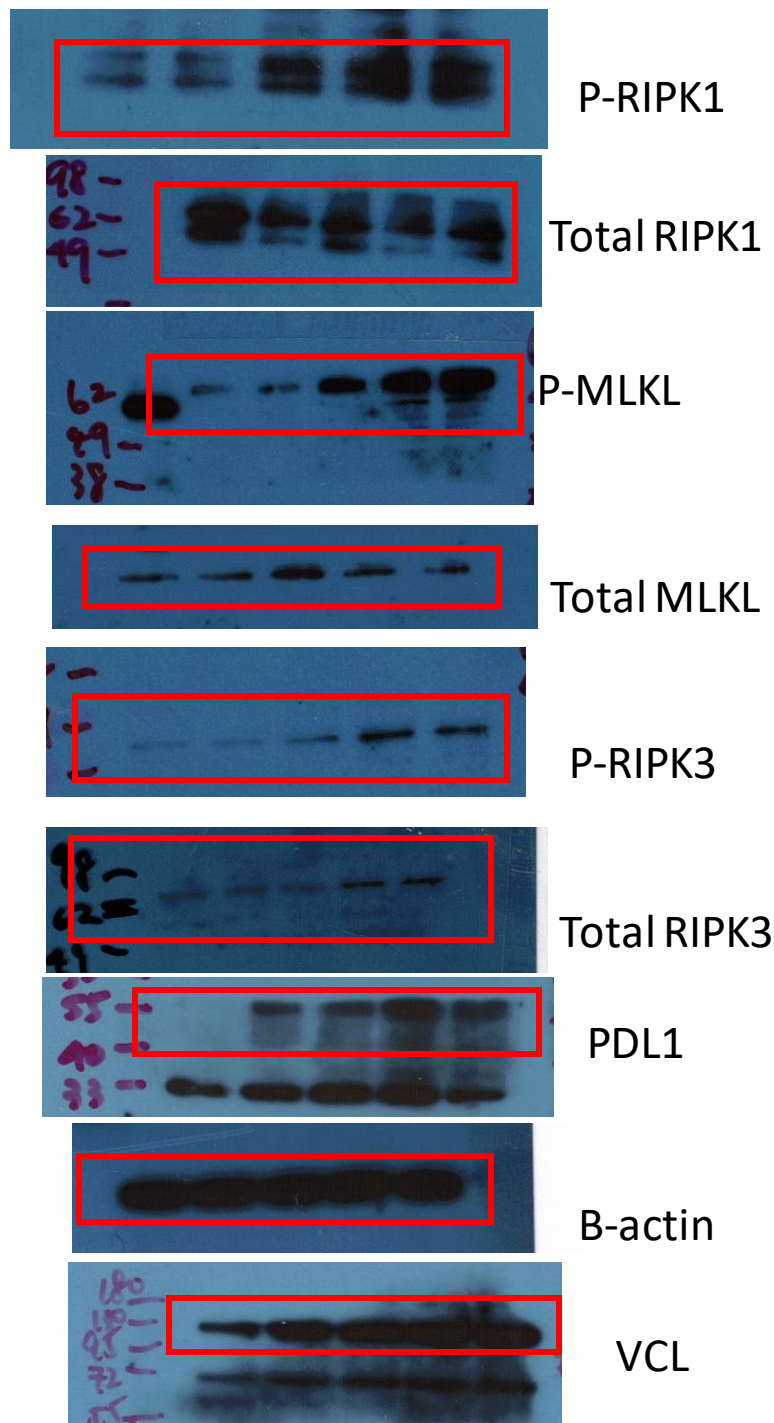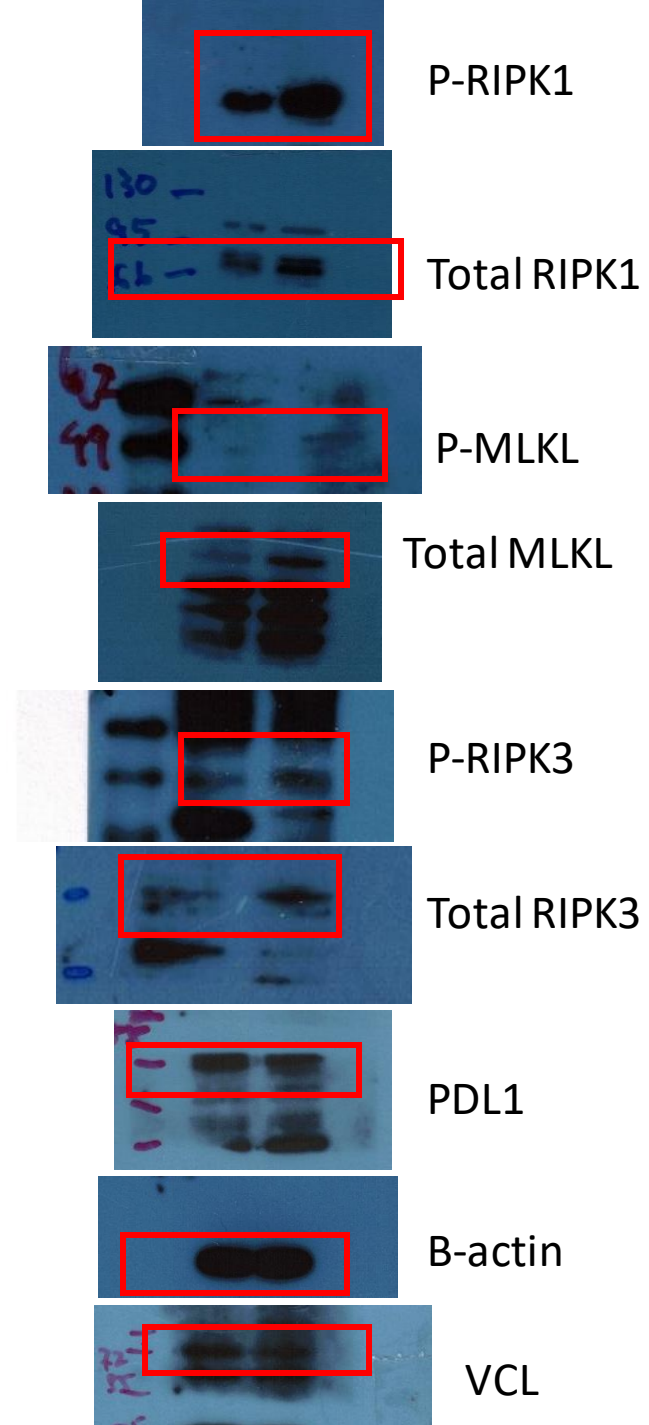

Fig 6H

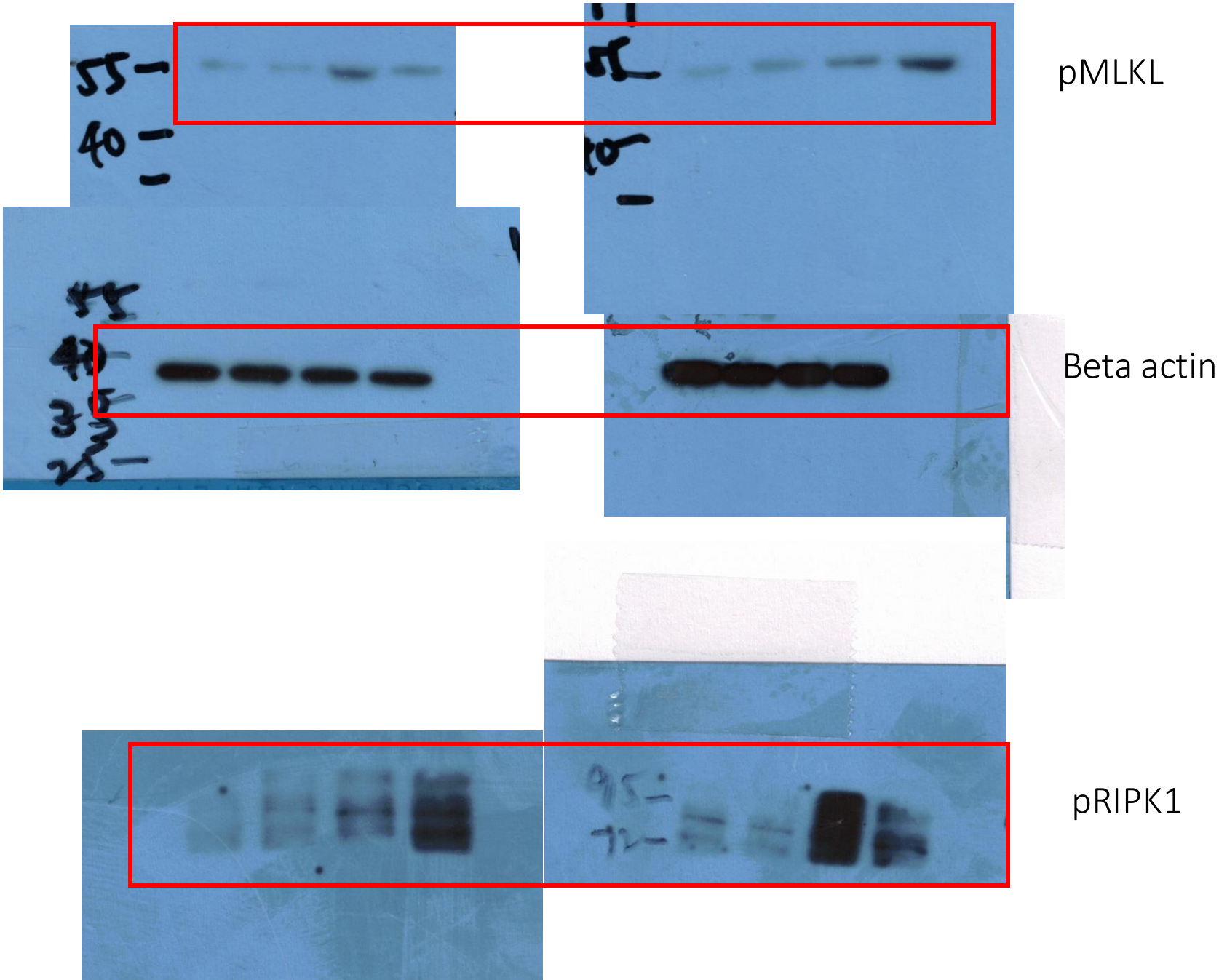

Supplement: Supplementary file 12 — WB raw [file 41423_2024_1253_MOESM12_ESM.pdf]
