# Supplementary material for: Cell-associated galectin 9 interacts with cytotoxic T cells confers resistance to tumor killing in nasopharyngeal carcinoma through autophagy activation
Source: Cell Mol Immunol. 2025 Feb 5;22(3):260–81. doi: 10.1038/s41423-024-01253-8 (PMC11868493; doi:10.1038/s41423-024-01253-8)
Supplement: Supplementary file 9 — Supplemetnary Informtaion(highlight) [file 41423_2024_1253_MOESM9_ESM.docx]

**Supplementary Information**

**Cell lines**

Two NPC cell lines (C17 and NPC43) kindly provided by Professor George Tsao were maintained in RPMI1640 medium (invitrogen) supplemented with 10% FBS, 1% P/S and 4 µM Y27632 ROCK inhibitor (Enzo Life Sciences,. ALX-270-333). All experiments were performed with cells before the 20th passage was reached. The HLA types of these cell lines are listed in Supplementary Table 1. All cell lines were regularly tested for mycoplasma infection and were authenticated.

**Isolation of human peripheral blood mononuclear cells (PBMCs) and CD8+ T cells**

PBMCs were isolated from buffy coats (Hong Kong Red Cross Blood Transfusion Service) of healthy donors by density gradient centrifugation method using Ficoll-Paque, as per manufacture recommendations (GE Healthcare). Briefly, blood samples were mixed with PBS and slowly layered on using Ficoll-Paque. The sample was then centrifuged at 400 g for 25 minutes at room temperature (RT) using slow acceleration and deceleration. The interface containing PBMCs was carefully removed and cells were washed twice with PBS + 2 mM EDTA, followed by cryopreservation in FBS/DMSO (90%/10%) for future analysis. Cryopreserved PBMCs were rapidly thawed and resuspended in RPMI1640 supplemented with 10% heat-inactivated foetal bovine serum (HI-FBS, Invitrogen), 1% Hepes and 1 % Penicillin/Streptomycin (Gibco). On day 3, and every 3–4 days thereafter, the cultures were supplemented with the appropriate growth medium containing 120 IU/mL of recombinant IL-2 (Miltenyi biotec). On day 7, cells were phenotyped and enumerated using Multitest 6-Color TBNK Reagent (BD Biosciences) to ensure enrichment of CD4^+^ and CD8^+^ cells in the cultured cells, before subsequent co-culture studies. For isolation of CD8+ T cells, 200 × 10^6^ PBMCs were incubated with 200 μL of antibody-based CD8 Microbeads (Miltenyi) were diluted to 1000 μL in binding buffer a for 15 min at 4 °C under gentle rotation, according to the manufacturer’s instructions.

**Expansion of LMP-/EBNA1-specific T cells**

The clinical grade AdE1-LMPpoly vector (polyepitope of 16 HLA-restricted LMP1&2 epitopes fused to a truncated gly/ala deleted EBNA1 gene) used in this study has been described previously (REF). After harvesting PBMC from blood, 30% of the PBMC was infected with AdE1-LMPpoly (multiplicity of infection of 10:1), which were then irradiated and co-cultured with the remaining PBMCs for two weeks. Cultures were supplemented with medium containing IL2 as previously described. *In vitro* expanded T-cells were tested for antigen specificity prior to release for mice injection. A summary of the EBV antigen specificity (HLA restriction) of transferred T-cells is shown in Supplementary Table 2.

**Palmitoylated and its control cell lines generation**

Briefly, the pPalmitoyl-mTurquoise2 insert from the pPalmitoyl-mTurquoise2 vector (Addgene, Inc.) was cloned into the lentiviral pLVX-EF1a to generate pLVX-pPalmitoyl-mTurquoise2.  Transcript of LGALS9 (NM_009587) was then amplified by PCR and to replace the mTurquoise2 construct, generating pLVX-pPalmitoyl-LGALS9 (pLVX-pal-LGALS9). Transcript of LGALS9 was also cloned into pLVX-EF1a to generate pLVX-LGALS9 (pLVX-LGALS9) as a wildtype control.  A pLVX-EF1a-GFP (was also utilized as an empty vector control. 293T cells were seeded in preparation for viral production in T25 culture flasks until reaching 50% confluence. The transfection mixture was prepared using 8µl X-tremeGENE™ HP DNA Transfection Reagent (Roche Molecular Systems, Inc.), 2 µg plasmid DNA, 1.5 µg viral packaging vector psPAX2 (Addgene, Inc.) and 0.5 µg viral envelope vector pMD2.G (Addgene, Inc.) in 400 µl serum-free DMEM. The transfection mixture was added dropwise to the HEK293T cells and incubated for 72 h at 37°C. Subsequently, the viral supernatant was collected and stored at −80°C. For lentiviral infection, NPC43 were seeded to achieve a confluency of ~30% at 24 h post-seeding. A 1 ml aliquot of pre-prepared virus expressing GFP, pal-LGALS9 or LGALS9 with 0.6 µl 5 µg/ml polybrene (Sigma-Aldrich; Merck KGaA) was added to cells in tissue culture flasks. The medium was changed at 24 h post-infection for cell recovery. The cells were passaged and check for overexpression status. The verification of the palmitoylated cell line was conducted in LSM800 confocal microscope with Airyscan module. Cross-section intensity of G9 and E-cadherin was done by profiling the signal by Zen 2.6 (blue edition).

**Single cell RNA sequencing (scRNA -seq) and transcriptomic expression analysis**

To evaluate the cell-cell communication of G9 with reported interacting partners, scRNA-seq data was gathered from three cohorts and UMAP was produced to visualize the immune cell types and tumor cell using the R package ‘Seurat’. Cell-cell interaction analysis was conducted by CellChat, a publicly available repository for ligand-receptor analysis. Average expression of T cell marker gene signature was shown as heatmap using the R package ‘ggplot2’. To explore the ligand and reported receptors expression in the three cohorts, average expression value was plotted in form of UMAP plots. To support the relationship among G9, autophagy and necrosis, a scRNA-seq dataset with the GEO accession number GSE150430 was utilized. All malignant cells from NPC patients (n=11) were subset for the correlation between necrotic cell death and proteolysis. Using gene sets in Gene Ontology Biological Pathways as references, necrotic cell death, proteolysis and autophagy scores were obtained by ‘UCell’ package in R. A score calculated by geneset expression level was assigned to every malignant cell. The higher the score is, the more enriched the geneset in the cells. Pearson correlation analysis was performed, with the generation of correlation plot among all 7581 cells between necrotic cell death and proteolysis was generated by ‘ggpubr’ package. Based on the median values of the scores, high/low group was assigned for necrotic cell death or proteolysis respectively. With the grouping, G9 average expression value was plotted in Graphpad Prism. The same grouping was applied for plotting autophagy score.

When indicated, GzB stratification was used to generate correlation plots between G9 expression values, UCell scores of autophagy and necrosis. We first applied Seurat to perform dimension reduction in the cohort and to generate a tSNE plot for identifying and clustering cell types. Average expression of T cell marker genes was shown as heatmap using ggplot2. Seurat was used to visualize GzB average and percentage expression in CD8+ T-1, T-2 and T-3 clusters from NPC patients’ (n=11), in the form of dotplots. Median average expression value was used as cutoff of GzB high (n=6) and low (n=5) groups.

**Animal experiments**

Assessment of tumour regression by combination of autophagy inhibitor and PDL1 blockade

This experiment is designed to assess the combination effect of the classical PDL1 blocker and autophagy inhibitor on G9 GOF NPC mice model. As mentioned above, PBMC were expanded, pre-activated and cocultured with NPC43OE6 at effector: target ratio of 1:1 for 6 hours. 6 μM NAC was added for 2 hours (MedChemExpress). Mice were irradiated and tumor cells + ex T cells were injected under conditioned as described above. On day 4 and day 8, 1mg/kg of Durvalumab (AstraZeneca) was injected intraperitoneally. Tumor growth was monitored from day 3 to day 10. Tumor from animals were collected for histological examination for PDL1 and RIPK1 expression and staining intensity was quantified.

Immunohistochemistry

Sections of 4 μm thickness were cut from formalin-fixed paraffin-embedded (FFPE) tumour tissue blocks. Slides were deparaffinized in xylene, rehydrated, and this was followed by an antigen retrieval step by heating at 95°C for 45 min in citrate buffer (pH6). Endogenous peroxidase was blocked with peroxidase blocking reagent (Dako) followed by a non-specific binding protein block (Dako, X0909). Sections were then incubated with primary antibodies either mouse anti-human CD8, rabbit anti-human LC3B and rabbit anti-human and a species-matched isotype control overnight at 4 °C. Slides were then washed and secondary staining was performed with Dako REAL EnVision Detection System (K5007, Dako) and visualized with diaminobenzidine (DAB) according to the kit’s instructions.

Multiplexed immunohistochemistry

Panel 1: A set of TMA was set to perform the tyramide signal amplification (TSA) IHC staining. Slides were first deparaffinized and rehydrated in serial passage through xylene and alcohol. Antigen retrieval was performed by microwaving the samples for 2 min 20 s with 100% power, followed by 20% power for 15 min and the slide was cooled for 20 min. Then, the sections were incubated with blocking solution, for 15 min at room temperature. Slides were incubated with primary antibodies: G9, CD8, CD4, Tim3 and Pan-cytokeratin for 1 h at room temperature. Multiplexed TSA was visualized using performing 5-plex Opal dyes. All multiplex TSA analyses were performed by repeating staining cycles in series, microwaving in between each cycle and at the end of the multiplex TSA. Slides were then counterstained with DAPI for 5 min and mounted with VECTASHIELD.

Panel 2: Another TSA IHC staining was performed on NPC tumor tissues and surrounding normal tissue collected by method similar to above. The primary antibodies RIPK1, G9 and Pan-cytokeratin were incubated overnight 4°C, while Beclin was incubated 1 hour at room temperature. Multiplexed TSA was visualized using performing 4-plex Opal dyes.

All antibodies used are listed in Table S6.

Digital image acquisition and analysis

TMA sections were digitally scanned at an absolute magnification of × 20 using the Vectra 3.0 Vectra Polaris imaging system (Akoya Biosciences) and analyzed with inForm Tissue Finder software (Akoya Biosciences). Multispectral images were unmixed using the spectral libraries built from images of single-stained slides. 15 TMA cores were initially selected to train machine learning algorithms for tissue segmentation, cell segmentation, and cell phenotyping, which were later applied on the whole TMA cohort. Cell segmentation was based on the nuclear DAPI stain, but assisted using membrane CD8, CD4, Tim3 and G9 (also with cytoplasmic) staining. The software was first trained to segment tissue by manually to segment tumor tissues into carcinoma, the intra- (epithelial), and the peri-(non-epithelial), areas based on tumor marker, pan-cytokeratin (PanCK; clone AE1/AE3; DAKO). Training sessions for tissue segmentation and phenotype recognition were carried out repeatedly until the algorithm reached the level of confidence recommended by the program supplier (at least 90% accuracy) before performing final evaluation. Each scanned image was examined by one observer under the supervision of an experienced pathologist. Exclusion criteria for whole TMA cores of 179 were; 1) loss of large part or whole core, 2) bad staining quality (e.g. weak pan-Cytokeratin stain), 3) lack of tumour or stromal tissue and clinical information. After exclusion, this led to 149 cores. Out of these cores, tumor cores (n=138) from 92 NPC patients and adjacent normal cores (n=11) from an additional 7 NPC patients were remained in the study. Within this cohort, 5 patients were excluded for spatial analysis due to bad quality of staining. After exclusions, 87 patients remained in the study, of which 23 patients were excluded for survival analysis due to lack of clinical information. In total, summary data was collected from 92 patients for tissue mass analysis, 87 patients for spatial analysis and 64 patients for the survival analysis. For patients with data on two TMA cores, an average number of cells per mm^2^ was calculated from the total number of cells divided by the total tissue area. The area of each tissue category, carcinoma, and stroma was evaluated to assess the density of lymphocytes, represented by (number of lymphocytes)/(pixel area mm^2^) in each tumor cores. Tumoral G9 and infiltrating immune cells, as identified by the different markers, were further divided into groups of high and low by the median number of cell densities.

Inter-cellular distance calculation package (Akoya) was used for distance analysis. The tool calculated the distance between two cell nuclei based on their x and y coordinates from the inForm data. The cutoff point is established at 300um, as less than 1% of cells in the tumor tissue were observed to exceed this distance. Each cell of the same phenotype served as a reference point to determine its distance to the nearest cell of a different phenotype. Effective percentage refers to the proportion of the reference cells that are paired with the nearest cells. A distance of 15 µm (nucleus to nucleus) was defined as the estimated direct contact distance between cells.

$$Effective percentage \left( \% \right)=\frac{Number of RC paired with NC}{Total number of RC}$$

For immunoreactivity (IR) within the tumor cells, the tumor region was identified using QuPath's built-in cell segmentation algorithms based on PanCK staining. The total amount of tumor cells in the tumor region was quantified using DAPI. Using a cell intensity-based measurement, the proportion of positive cells phenotypes of interest (PPC) within the total tumor cells equaled the cell count of cell phenotypes of interest divided by total DAPI count. The IR was then calculated by multiplying the PPC by staining intensity.

$$Immunoreactivity \left( \% \right)=\frac{PPC}{Total number of DAPI in tumor region} X staining intensity$$
