# Supplementary material for: Cell-associated galectin 9 interacts with cytotoxic T cells confers resistance to tumor killing in nasopharyngeal carcinoma through autophagy activation
Source: Cell Mol Immunol. 2025 Feb 5;22(3):260–81. doi: 10.1038/s41423-024-01253-8 (PMC11868493; doi:10.1038/s41423-024-01253-8)
Supplement: Supplementary file 10 — Supplementary Figure legend(highlight) [file 41423_2024_1253_MOESM10_ESM.docx]

**Supplementary figure legend**

**Supplementary Figure 1. Expression of G9 and T-cell population in NPC.**

(**A**) Box plot showing transcriptional expression of LGALS9 (G9) in NPC and normal nasopharyngeal (NP) tissues [GEO databases, GSE12452 (left) and GSE53819 (right)]. Data are presented as mean ± SEM. (**B**) Segmentation of the tumor compartment into intratumor (IT; red) and peritumor (PT; green) regions, which were distinguished by PanCK cells. (**C**) Patient stratification (left) Kaplan–Meier overall survival analysis (right) based on PT-associated CD8 T cell and IT-associated G9 expression of cancer patients (median as cut-off). (**D**) Representative staining cores with tumor marker (pan-cytokeratin-5, PanCK; green), CD8 (orange), CD4 (pink), G9 (red), Tim3 (white) and nuclei (DAPI, blue). based on IT-associated CD8 T cell and G9 expression. (**E**) Box plot shows the distance of CD4 and CD8 T-cell populations (Tim3^+^ or Tim3^-^) to G9-high and G9-low-expressing tumor cells. Box and whiskers represent median ± 25–75 percentile. Median distance of each population was shown on the right. (**F**) Correlation matrix between different tumor signatures and immune cell density in the IT zone of NPC tissues. Each ellipse in the table represents a correlation between the two variables. The color indicates the direction of change, red=positive association, blue=negative association. (**G**) Two individual representative TMA cores (left) of the IT and PT regions of tumor tissues were selected for Tim3^+^ cell quantification in 87 samples and quantitative data (right) on Tim3^+^ cell densities as counts/mm^2^ in the IT and PT regions of tumor tissues. (**H**) The histogram represents the proportion of reference cells (RC) within a certain nearest distance (range from 0-300µm) between RC and NC. Shown are pairs of distance analysis from 87 patients where the NC is G9-expressing tumor cells (PanCK+G9+). A direct cell-contact distance within a range from 0-15µm is indicated by dashed red rectangle. *p < 0.05, **p < 0.01, ***p < 0.001 and ****p < 0.0001 (Mann–Whitney test for A, E, G, Kaplan–Meier curves were analyzed using two-tailed log-rank test for C, Pearson correlation for F).

**Supplementary Figure 2. Construction of knockdown (KD) and overexpression (OE) cell lines on G9 expression**

(**A**) Selection of cell lines for KD and OE experiments from a panel of patient-derived NPC cell lines based on basal expression of G9 transcript levels. The Ct. (cycle threshold) for each cell line is indicated. (**B**) Flow cytometric analysis of cell conjugate formation among PKH26-labeled PBMC and PKH67-labeled tumor cells. Representative confocal images of cell conjugates (arrows).

**Supplementary Figure 3. Characterization of T-cell expansion and function after cocultured with tumor cells.**

(**A**) Flow cytometry picture of peripheral blood mononuclear cell expansion of CD3/CD4/CD8 T cells (Ex-T cell) gated from CD45^+^ lymphocytes before (top panel) and after cocultures (bottom panel). The T cell product used for coculture experiments was denoted as rapid-expansion T cells (REP-T cells). (**B**) Viability of PBMC as detected by CellTiter-Glo over 7-day cocultured with tumor cells. (**C**) Annexin V antibody labels in tumor cells after cocultures were detected by flow cytometry. (**D**) Representative FACS analysis of CFSE staining at day 5. Cell generations were identified (different colour) after ex-PBMC cocultured with indicated tumor cells at day 0 (grey shadow). The data were pooled from three (n = 3) independent experiments. **P < 0.01 (two-way ANOVA for B).

**Supplementary Figure 4. T cells are associated with their proximity to tumor cells with G9-dependency**

(**A**) The proportions of each response pattern contributing to the total gated CD4 response after 5-day cocultured with TarTC are shown. Pie charts representing the polyfunctional profile (granzyme B; GzB, Ki67, TNFα or IFNγ or in combination). The size of the pie segment correlates to the frequency of corresponding colour-coded cytokines. The arcs around the circumference represent specific cytokines (GzB in orange, IFNγ in turquoise, Ki67 in red, TNFα in black) produced by the proportion of cells that lie under the arc. Parts of the pie surrounded by multiple arcs represent polyfunctional cells. (**B**) Heatmap display showing relative expression levels of genes expressed by ex-CD8^+^ T cells post cocultured. The scale to the top shows the expression level (as determined by CT value). (**C**) Flow cytometric histograms illustrate granzyme B (GzB) production in ex-T cells after cocultured with TarTC directly or indirectly via a transwell for 3 days. (**D**) Representative staining (left) and quantitative data (right) in NPC tissues expressed with G9-positive (n=2) and G9-negative (n=2) markers. The barplot represent the percentage of reference cells (RC: PanCK+G9-positive or negative) within 50 µm between RC and nearest cell (NC: CD8+GzB+) (right). **(E)** Representative zebra plot of GranToxiLux assay. The data were pooled from three (n = 3) independent experiments. **P < 0.01, ***P < 0.001 and ****P < 0.0001 (two-tailed unpaired Student’s t test for B and E). Data points are shown ± SEM.

**Supplementary Figure 5. G9 expression alteration does not affect tumor cell proliferation but promotes cell death**

**(A)** Cell colony formation assay of G9-overexpressed or knocked-down TarTC upon cocultured with CTL. The number of cell clones was counted under the microscope in 5 randomly selected fields in each well, and four separate experiments were performed for average. (**B**) XTT assay of G9-overexpressed or knocked-down TarTC upon cocultured with CTL. (**C**) Propidium iodide (PI) label was detected by flow cytometry in PKH67-labelled TarTC. Representative results are expressed as zebra plot. (**D**) Bar plot of live/dead ratio and stacked bar chart with means of three independent experiments. (**E**) Representative results are expressed as zebra plot. NPC TarTC cocultured with CTL were analysed on the CellROX staining gated from Epcam^+^ NPC TarTC. (**F**) Flow cytometry analysis of Annexin V and propidium iodide (PI) signals in NPC TarTC cocultured with or without (**G**) CTL. Representative results are expressed as zebra plot. (**H**) Representative results are expressed as zebra plot. NPC alone were analysed on the CellROX staining gated from Epcam^+^ NPC TarTC. The data are presented as the mean ± SEM. Two-tailed unpaired Student’s t test was used. *P < 0.05 and ****P < 0.0001.

**Supplementary Figure 6. Autophagy in NPC cells upon conjugation with CTL**

(**A**) Timeline of the procedure for fluorescence staining. A: antiCD3/28 activation of ex-CD8+ T cells; C: coculture for 6 hours; L: LysoTracker Red staining on NPC TarTC and stained with mouse anti-human LC3B and developed with anti-mouse Alexa488. Nuclei were stained in blue in NPC cells. Representative fluorescence photographs of the colocalization of LC3B (green) and LysoTracker (Red) in cocultured TarTC. (**B**) Western blot on NPC tumor cells for indicated autophagy and necrosis marker. (**C**) Quantification of autophagic vesicles in NPC cells cultured alone. Data presented as green signal normalized with blue DAPI signal. (**D**) Western blot on NPC vector control cells treated with specific autophagy inhibitors: N-acetylcysteine =NAC (6uM); chloroquine = CQ (60uM), pepstatin A = pep (10ug/ml) for indicated autophagic markers. The data were pooled from three (n = 3) independent experiments. Data points are shown ± SEM. (**E**) Autophagosomes (left column) and SYTOX Green stain fluorescence detection of cell necrosis (right column) with or without indicated doses of G9 inhibitors. (**F**) Live/dead staining showed the difference between coculture groups.

**Supplementary Figure 7. Relationship among autophagy, G9 and necrosis in NPC patients**

(**A**) Flow chart for the calculation of immunoreactivity that recapitulates key features of necrosis (RIPK1) and autophagy (Beclin) in tumor regions of NPC patient tissue samples. (**B**) Scatterplot comparing high (Hi) and low (Lo) magnitude of proteolysis (Proteo) and necrosis (Necro) signature score of all malignant cells from NPC patients (n=11) of GSE150430. The quadrant (classes I–IV) are divided by the median value of gene signature score. (**C**) Barplot showing average expression of G9 (left) and autophagy signature score (right) in class I vs. IV. (**D**) Dot plot showing expression of granzyme B (*GzB*) production in CD8^+^ CTLs by patients (n=11; GSE150430). The high (*GzB*-high) and low (*GzB*-low) groups are divided by the median value of average gene expression. (**E**) Computed differences in Pearson correlation (Δr) for indicated genes in high and low groups. Color indicates direction of change, that is, red=positive association, blue=negative association. Only significant associations are shown. The data were pooled from three (n = 3) independent experiments. Data points are shown ± SEM. *P < 0.05 and ****P < 0.0001 (Mann–Whitney test for C and Pearson correlation for E).

**Supplementary Figure 8. Generation of allogeneic EBV-specific T cells using AdE1-LMPoly.**

(**A**) The phenotypic characteristics of AdE1-LMPpoly-stimulated T cells were evaluated through the standard TBNK analysis, to measure the surface expression of CD3 (T cells), CD8 (CD8+ T cells), CD4 (CD4+ T cells), CD16 and CD56 (natural killer cells), and CD19 (B cells). (**B**) T-cell cultures were stimulated with a peptide pool containing EBNA1, LMP1 and LMP2 peptide epitopes and then assessed for intracellular IFN-γ expression. Representative flow cytometry plots show the percentage of CD4+ and CD8+ T cells demonstrating EBV epitope-specific reactivity in the T-cell product (P-HiWo). (**C**) Cytotoxicity assays were performed on PKH67-labelled tumor target cells (C17KD4 or C17Vector control cells) cocultured with anti-CD3/28 activated pre-REP CD8^+^ T cell at indicated effector:target ratio. The percentage of tumor cell lysis was determined by flow cytometry.
