# Supplementary material for: Cell-associated galectin 9 interacts with cytotoxic T cells confers resistance to tumor killing in nasopharyngeal carcinoma through autophagy activation
Source: Cell Mol Immunol. 2025 Feb 5;22(3):260–81. doi: 10.1038/s41423-024-01253-8 (PMC11868493; doi:10.1038/s41423-024-01253-8)
Supplement: Supplementary file 11 — Supplementary Table [file 41423_2024_1253_MOESM11_ESM.docx]

**Supplementary Table 1. EBV-associated cancer cell lines used in the study**

| Cancer cell lines (origin) | HLA typing* | HLA-matched allogeneic EBV-specific T-cell products |
| --- | --- | --- |
| C17 (NPC) | **A*02:01**, 26:01; B*44:02,51:01; C*05:01,14:02 | P-HiWo |
| NPC43 (NPC) | A*11:01, 11:01; B*50:01, 50:01; C*06:02, 06:02 | NA |

*HLA alleles matched between the cancer cell lines and allogeneic T-cell products are underlined. EBV, Epstein-Barr virus; HLA, human leukocyte antigen. NA, not available

**Supplementary Table 2. EBV antigen specificities and HLA restrictions of allogeneic T cells used in the study**

| Allogeneic EBV-specific T-cell product | EBV antigen specificity (HLA restriction) |
| --- | --- |
| P-HiWo | LMP1 (YLLEMLWRL/A*02:01, YLQQNWWTL/A*02:01); LMP2 (FLYALALLL/A*02:01); EBNA1 (FVYGGSKTSL/C*03:04) |

EBNA1, EBV-encoded nuclear antigen 1; EBV, Epstein-Barr virus; HLA, human leukocyte antigen; LMP1, latent membrane protein 1; LMP2, latent membrane protein 2.

**Supplementary Table 3. Flow cytometry antibodies**

|  | |  |  |  |
| --- | --- | --- | --- | --- |
| Specificity | Fluorochrome | Reagent Description | Clone | Catalog Number |
| *In vitro* cocultures | | | | |
| CD3 | Alexa700 | BD Pharmingen™ Alexa Fluor 700 Mouse anti-Human CD3 | UCHT1 | 557943 |
| CD4 | BV510 | BD Horizon™ BV510 Mouse Anti-Human CD4 100 Tests | SK3 (also known as Leu3a) | 562970 |
| CD45 | BV786 | BD Horizon™ BV786 Mouse Anti-Human CD45 | HI30 | 563716 |
| IFNγ | BV421 | BD Horizon™ BV421 Mouse Anti-Human IFNγ | 4SB3 | 564791 |
| CD107a (LAMP-1) | PE-Cy7 | BD Pharmingen™ PE-Cy™7 Mouse Anti-Human CD107a 50 Tests | H4A3 | 561348 |
| CD8 | APC-H7 | BD Pharmingen™ APC-H7 Mouse anti-Human CD8 | SK1 | 560273 |
| Granzyme B | PE | BD Pharmingen™ PE Mouse Anti-Human Granzyme B 100 Tests | GB11 | 561142 |
| Ki-67 | BV711 | BD Horizon™ BV711 Mouse Anti-Human Ki-67 | B56 | 563755 |
| TNFα | BV650 | BD Horizon™ BV650 Mouse Anti-Human TNFα | MAB11 | 563418 |
| CD326 (EpCAM) | PE-CF594 | BD Horizon™ PE-CF594 Mouse Anti-Human CD326 | EBA-1 | 565399 |
| ACT functionality | | | | |
| CD8 | PerCP Cy5.5 | eBioscience | RPA-T8 | 45-0088-42 |
| CD4 | Pacific Blue | BD Biosciences | RPA-T4 | 558116 |
| IFN-γ | AF700 | BD Biosciences | B27 | 557995 |
| 6-color TBNK Reagent | - | BD Biosciences | - | 662967 |

**Supplementary Table 4. Western blot antibodies**

| Primary antibody | Company | Cat no | Dilution |
| --- | --- | --- | --- |
| p-AMPK | ABclonal | AP0883 | 1/1000 |
| mTOR | ABclonal | A11354 | 1/500 |
| p-mTOR | ABclonal | AP0094 | 1/500 |
| Beclin-1 | Abcam | AB62557 | 1/1000 |
| LC3 | ABclonal | A11282 | 1/1000 |
| p-RIPK1 | ABclonal | AP1230 | 1/1000 |
| RIPK1 | ABclonal | A7414 | 1/1000 |
| p-RIP3 | ABclonal | AP1260 | 1/1000 |
| RIPK3 | ABclonal | A5431 | 1/1000 |
| p-MLKL | ABclonal | AP0949 | 1/1000 |
| MLKL | ABclonal | A5579 | 1/1000 |
| MMP9 | proteintech | 10375-2-AP | 1/1000 |
| p62 | ABclonal | A11483 | 1/1000 |
| β-actin | Abcam | ab6276 | 1/5000 |
| Secondary antibody | Company | Cat no | Dilution |
| Anti-rabbit | Cytiva | NA934 | 1/10000 |
| Anti-mouse | Cytiva | NA931 | 1/10000 |

**Supplementary Table 5. Primers list**

| qRT-PCR primers | |
| --- | --- |
| Granzyme B-F | 5′-CCATCCATCCAAGCCTATAATCCTA-3′ |
| Granzyme B-R | 5′-CCTGCACTGTCATCTTCACCT-3′ |
| Eomes-F | 5′-CTGCCCACTACAATGTGTTCG-3′ |
| Eomes-R | 5′-GCGCCTTTGTTATTGGTGAGTTT-3′ |
| Tbx21-F | 5′-GGTTGCGGAGACATGCTGA-3′ |
| Tbx21-R | 5′-GTAGGCGTAGGCTCCAAGG-3′ |
| Ccl5-F | 5′-CCAGCAGTCGTCTTTGTCAC-3′ |
| Ccl5-R | 5′-CTCTGGGTTGGCACACACTT-3′ |
| TNFα-F | 5′-ATGAGCACTGAAAGCATGATCC-3′ |
| TNFα-R | 5′-GAGGGCTGATTAGAGAGAGGTC-3′ |
| IFNγ-F | 5′- GAATTGGAAAGAGGAGAGTGACAG-3′ |
| IFNγ-R | 5′- TAGCTGCTGGCGACAGTTCA-3′ |
| CCR7-F | 5′-GATACCTACCTGCTCAACC -3′ |
| CCR7-R | 5′-AAGTGGACACCGAAGACCC -3′ |
| PRF1-F | 5′- GGCTGGACGTGACTCCTAAG-3′ |
| PRF1-R | 5′- CTGGGTGGAGGCGTTGAAG -3′ |

**Supplementary Table 6. Immunohistochemistry and immunocytochemistry antibodies**

| Primary antibody | Company | Cat no | Dilution |
| --- | --- | --- | --- |
| Beclin-1 | Abcam | AB62557 | 1/100 |
| LC3B | ABclonal | A11282 | 1/100 |
| Caspase-8 | Cell Signalling Technology | 9746s | 1/100 |
| Granzyme B | Abclonal | A2557 | 1/200 |
| E-cadherin | Abcam | ab76055 | 1/300 |
| Galectin-9 | Abcam | ab69630 | 1/100 |
| Pan-Cytokeratin | Dako | M0821 | 1/100 |
| CD4 | Dako | M7310 | 1/50 |
| CD8 | Dako | IS634 | - |
| Tim3 | Cell signalling | 45208S | 1:200 |
| RIPK1 | ABclonal | A7414 | 1:100 |

**Supplementary Table 7. Drug and treatment**

| Drug | Company | Cat no |
| --- | --- | --- |
| Pepstain-A | MedChemExpress | HY-P0018 |
| N-acetyl-l-cysteine (NAC) | MedChemExpress | HY-B0215 |
| Chloroquine | MedChemExpress | HY-17589A |
| Durvalamab | AstraZeneca | MEDI4736 |
| Lactose | MedChemExpress | HY-B2123 |
| Anti-Galectin-9 Antibody, Neutralizing | MERCK | MABT834 |
